# Annotated Checklist of Poroid Hymenochaetoid Fungi in Central Asia: Taxonomic Diversity, Ecological Roles, and Potential Distribution Patterns

**DOI:** 10.3390/jof11010037

**Published:** 2025-01-05

**Authors:** Yusufjon Gafforov, Manzura Yarasheva, Xue-Wei Wang, Milena Rašeta, Yelena Rakhimova, Lyazzat Kyzmetova, Kanaim Bavlankulova, Sylvie Rapior, Jia-Jia Chen, Ewald Langer, Burkhon Munnavarov, Zafar Aslonov, Bobozoda Bakokhoja, Li-Wei Zhou

**Affiliations:** 1 Central Asian Center of Development Studies, New Uzbekistan University, Tashkent 100007, Uzbekistan; 2 Mycology Laboratory, Institute of Botany, Academy of Sciences of Republic of Uzbekistan, Tashkent 100125, Uzbekistan; 3 State Key Laboratory of Mycology, Institute of Microbiology, Chinese Academy of Sciences, Beijing 100101, China; xuewei_wang1995@im.ac.cn; 4 Microbiology Laboratory, Navruz International Corp. LLC., Salar Settlement, 111219 Kibray, Uzbekistan; manzuryarasheva@gmail.com; 5 University of Chinese Academy of Sciences, Beijing 100049, China; 6 Department of Chemistry, Biochemistry and Environmental Protection, Faculty of Sciences, University of Novi Sad, 21000 Novi Sad, Serbia; milena.raseta@dh.uns.ac.rs; 7 Mycology and Algology Laboratory, Institute of Botany and Phytointroduction, Almaty 050040, Kazakhstan; evrakhim@mail.ru (Y.R.); lyzka79@mail.ru (L.K.); 8 Laboratory of Mycology and Phytopathology, Institute of Biology, National Academy of Sciences, Bishkek 720071, Kyrgyzstan; bavlankulova.k@list.ru; 9 CEFE, University of Montpellier, CNRS, EPHE, IRD, 15 Avenue Charles Flahault, 34093 Montpellier Cedex 5, France; sylvie.rapior@umontpellier.fr; 10 College of Landscape Architecture, Jiangsu Vocational College of Agriculture and Forestry, Zhenjiang 212400, China; jiajiachen@jsafc.edu.cn; 11 Department of Ecology, University of Kassel, 34132 Kassel, Germany; elanger@uni-kassel.de; 12 Tashkent State Dental Institute, Tashkent 100047, Uzbekistan; munavvarov.burkhon@mail.ru (B.M.); azafarjon@list.ru (Z.A.); 13 Institute of Botany, Plants Phisyology and Genetics, National Academy of Sciences of Tajikistan, Dushanbe 734042, Tajikistan; bako.76@mail.ru

**Keywords:** basidiomycetes, conservation, ecological functions, forest macrofungi diversity, fungal taxonomy, habitat modeling, host preferences, Hymenochaetaceae, wood-inhabiting macrofungi

## Abstract

Central Asia, located at the heart of Eurasia, is renowned for its varied climate and vertical vegetative distribution, which support diverse biomes and position it as a global biodiversity hotspot. Despite this ecological richness, Central Asia’s fungal diversity, particularly wood-inhabiting macrofungi, remains largely unexplored. This study investigates the diversity, ecological roles, and potential distribution of poroid Hymenochaetoid fungi in the region. By conducting field surveys, collecting basidiomes, and reviewing the literature and herbarium records from five Central Asian countries, we compiled a comprehensive checklist of these fungi. In total, 43 Hymenochaetoid species belonging to 18 genera were identified, with *Inonotus*, *Phellinus*, and *Phylloporia* being the most species-rich. Notably, *Inonotus hispidus* and *Phellinus igniarius* were found to be the most widespread species. These macrofungi play essential ecological roles as saprotrophs and pathogens of various identified host plant families, aiding in lignin degradation and exhibiting diverse enzymatic activities. For the first time, we modelled the potential distribution patterns of Hymenochaetoid fungi in Central Asia, revealing that their distribution is strongly influenced by host plant availability and temperature-related factors. The three most critical variables were host plant density, annual temperature range (Bio7), and mean temperature of the warmest quarter (Bio10). The distribution of suitable habitats is uneven, with highly suitable areas (4.52%) concentrated in the mountainous border regions between Kazakhstan, Kyrgyzstan, Tajikistan, and Uzbekistan. These results underscore the significance of specific environmental conditions for the growth and survival of Hymenochaetoid fungi in this region. Our findings highlight the urgent need for continued mycological and host plant research and expanded conservation initiatives to document and preserve macrofungal and botanical biodiversity in this under-explored area. In light of climate change, the collected mycological and botanical data provide a valuable reference for promoting forest health management globally.

## 1. Introduction

Central Asia, covering from the Caspian Sea to Mongolia and western China and from Afghanistan and Iran to Russia, is known for its rich biodiversity and diverse geography. The Central Asian Mountains, a biodiversity hotspot [1], include the Pamir, Tian Shan, and Karakoram ranges, crossing Afghanistan, China, Kazakhstan, Kyrgyzstan, Tajikistan, Turkmenistan, Uzbekistan, Pakistan, and India. The region features towering mountains, vast deserts like Kara-Kum and Taklimakan, and expansive steppes. It belongs to the Palearctic Ecozone, forming the Eurasian steppe extending into Eastern Europe [2]. The Central Asian Mountains Hotspot, covering 860,000 km^2^ across parts of Kazakhstan, Kyrgyzstan, Tajikistan, Uzbekistan, China, Afghanistan, and Turkmenistan, is dominated by rocky, sparsely vegetated ranges reaching up to 7495 m. Key biomes include grasslands, deserts, shrublands, and coniferous forests [1,3,4].

The fungal diversity and fungus-like organisms in Central Asia remain poorly documented, despite the region’s vascular plant flora being estimated at approximately 10,000 species [3,4]. Globally, the fungi-to-plant species ratio is estimated at six to one [5], with studies conducted in regions of high plant endemism, such as the tropics and subtropics, frequently revealing higher ratios and numerous endemic fungal taxa [6,7,8,9,10,11]. However, knowledge regarding wood-inhabiting basidiomycetes and related fungus-like organisms in Central Asia is still highly fragmented [12,13,14,15,16,17,18,19]. The number of described fungal species in mountainous areas is significantly lower than that of plants, suggesting that a substantial number of fungal taxa remain undescribed. As a recognized biodiversity hotspot, Central Asia’s mountainous ecosystems require extensive field investigations to enhance understanding of fungal diversity. Yet, logistical challenges in accessing remote regions, combined with the region’s high endemism, have hindered taxonomic advancements. Current data indicate the presence of more than 150 wood-inhabiting basidiomycete species in Uzbekistan [15]; however, similar data for other Central Asian countries remain scant or entirely absent. The diversity of the Hymenochaetaceae, a family of wood-inhabiting basidiomycetes, is particularly understudied in this region [15,17,18,19], although new species and records have been sporadically reported from Uzbekistan, Kazakhstan, and Kyrgyzstan [15,17,18,19,20,21,22,23,24,25]. Preliminary surveys in the Western Tian Shan and Pamir Mountains indicate these areas likely harbor numerous endemic and novel fungal taxa [26,27,28,29,30,31,32,33,34]. The region’s high levels of plant endemism and diversity further support the expectation of undocumented fungal species. Central Asia’s flora, comprising approximately 10,000 vascular plant species, including a range of shrubs, annuals, perennials, and trees, has been extensively studied for its ecological and economic significance [3,35,36]. However, comprehensive studies on its associated fungal diversity remain critically lacking, highlighting the need for focused research efforts to elucidate the mycological wealth of this unique region [15,16].

Hymenochaetoid taxa are among the largest and most widely distributed fungal groups within the Basidiomycota, comprising macrofungi with diverse fruiting body forms. Molecular studies have established that the family Hymenochaetaceae constitutes a distinct phylogenetic entity, referred to as the “Hymenochaetoid clade” [37], providing critical evidence for its delineation [38]. According to Kirk et al. [39], this family initially included 27 genera and 487 species. However, recent molecular investigations have revealed that the Hymenochaetoid clade now encompasses 15 families, comprising a total of 65 genera and approximately 1300 species [40,41]. Within the Hymenochaetaceae, wood-decaying basidiomycetes play an essential ecological role as both saprotrophs and parasites. Owing to their enzymatic capabilities, many species efficiently degrade lignin, with some known to infest hardwoods and conifers, resulting in wood heart rot, cankers, and root diseases [42,43,44]. Beyond their ecological significance, several Hymenochaetoid species have been traditionally used to treat various human ailments. Their bioactive properties include antiallergic, antidiabetic, anti-inflammatory, antimicrobial, antioxidant, and hepatoprotective effects, among others [16,45,46,47,48,49,50,51].

This study aims to investigate the diversity, distribution, host preferences, and ecological roles of poroid Hymenochaetaceae species originating from Central Asia. Additionally, it will present a comprehensive checklist of Hymenochaetoid basidiomycetes, document new records from the study area, discuss the species currently recognized in the region, and assess their potential distribution patterns.

## 2. Materials and Methods

### 2.1. Study Area: Vegetation and Climate Characteristics of Central Asia

Central Asia encompasses diverse vegetation biomes, including temperate grasslands, savannas, deserts, xeric shrublands, and coniferous forests. It is a center of origin and diversity for globally significant crops such as apples, apricots, pomegranates, pears, and currants, with wild ancestors contributing vital genetic resources [52]. Despite its mountainous terrain, forest cover is limited, with desert and semi-desert regions dominated by *Haloxylon* (Amaranthaceae) and various shrubs. In moist mountainous areas, key species include junipers (*Juniperus* spp.), Asian spruce (*Picea schrenkiana*), Siberian fir (*Abies sibirica*), and walnuts (*Juglans regia*), alongside various fruit-bearing trees and shrubs [3]. Major rivers such as the Amu Darya and Syr Darya and water bodies like the Aral Sea and Lake Balkhash form part of the vast endorheic basin that includes the Caspian Sea. The region’s climate is strongly continental, characterized by arid to semi-arid conditions, low precipitation, and significant seasonal temperature variations. Summers in lowland areas are intensely hot, often exceeding 40 °C, with desert regions of Uzbekistan and Turkmenistan surpassing 45–50 °C. Winters range from mild in the south, averaging 5–10 °C, to severe in the north, where temperatures drop below −20 °C, reaching extremes of −30 °C to −40 °C in northern Kazakhstan during cold spells [53,54]. Precipitation is sparse, ranging from 0–5 mm in the dry summer months to 20–40 mm in autumn, peaking at 50–70 mm in spring, and then decreasing to 10–20 mm in early summer. These patterns highlight the arid nature of the region, with precipitation mainly concentrated in spring and autumn [53,54,55].

### 2.2. Collection and Preservation of Specimens

This study is based on fresh basidiomes of Hymenochaetoid fungi collected during field surveys conducted in urban and mountainous areas of the study region. Additional data on fungi from other Central Asian countries were obtained from the TAAM Herbarium (Estonian University of Life Sciences, Tartu) via the web-based biodiversity information platform PlutoF [56,57], the TASM Herbarium (Academy of Sciences of Uzbekistan, Tashkent), and the Mycology Herbarium (Institute of Biology, National Academy of Sciences, Bishkek). Supplementary sources included local libraries, scientific articles, research reports, books, and personal collections. Fungal specimens were examined in the mycological herbaria of research institutes to determine their taxonomic composition, referencing various books and monographs. After morphological examination, the collected samples were deposited in relevant herbaria. Fungarium acronyms used in this paper conform to the Index Herbariorum [58]. Scientific names of fungi and their hosts were verified for potential synonyms using Index Fungorum [59], Wang et al. [40], Wu et al. [60], Zhou et al. [61], and Plants of the World Online [62].

### 2.3. Data Compilation and Analysis

A current checklist of poroid Hymenochaetoid species was compiled through an exhaustive evaluation of the literature, herbaria, herbarium databases, GBIF, unpublished expert information, and a targeted search for species not yet documented in Central Asia but known to occur in Kazakhstan (KZ), Kyrgyzstan (KR), Tajikistan (TJ), Turkmenistan (TR), and Uzbekistan (UZ). Additionally, scientific search engines and online inventories were screened. Data were obtained from papers, monographs, and books written in local languages Kazakh, Kyrgyz, and Uzbek as well as in Russian and English, from indexed and non-indexed journals using online literature databases such as Google, Google Scholar, PubMed, ResearchGate, Scopus, Semantic Scholar, Web of Science, and ScienceDirect Navigator. Local library sources and personal materials were also used, with a focus on Hymenochaetoid species. As a result, 94 published articles were identified, from which relevant studies on the diversity, geographical distribution, ecology, and taxonomy of medicinal Hymenochaetoid fungi were selected. Furthermore, the reference lists of the selected literature were analyzed to gather more comprehensive and accurate information.

### 2.4. Species Occurrence and Habitat Suitability Modeling

#### 2.4.1. Species Occurrence Data

The occurrence records of poroid Hymenochaetoid fungi were extracted from the GBIF (Global Biodiversity Information Facility) [63] and supplemented with analysis of the literature sources and various herbarium of five countries in Central Asia (e.g., [14,15,16,17,18,19,20,21,22,23,24,25,33], which resulted in a total of 762 records (Table S1). Using Google Earth [64], latitude and longitude data were collected for the literature source distribution points of Hymenochaetoid fungi where coordinates were not initially available. When compiling the annotated species checklist for this paper, for the sake of conciseness, all occurrence records taken in Kazakhstan, Kyrgyzstan, Tajikistan, Turkmenistan, and Uzbekistan are listed in Table S1.

#### 2.4.2. Environmental Variables

A total of 19 current bioclimatic indicators and corresponding altitude data were downloaded from WorldClim version 2.1 database [65]. These environmental variables from the climate data for 1970–2000 at a spatial resolution of 30″ (approximately 1 km^2^) [65] were used for predicting the current geographic distribution of Hymenochaetoid fungi in Central Asia.

Due to the close relationship between Hymenochaetoid fungi and their host plant, the host plant was also considered as one of the most important covariates restricting the growth of Hymenochaetoid fungi. Therefore, the distribution of each host plant genus associated with Hymenochaetoid fungi (Table S1) was retrieved from the Global Biodiversity Information Facility [63] as one of the variables. The number of host plants on each coordinate was converted to raster data by ArcGIS v.10.8 [66] at a spatial resolution of 30” (approximately 1 km^2^) for predicting the current geographic distribution of Hymenochaetoid fungi in Central Asia (Figure S1).

#### 2.4.3. MaxEnt Model Optimization and Potential Habitat Evaluations

**Figure 1:**
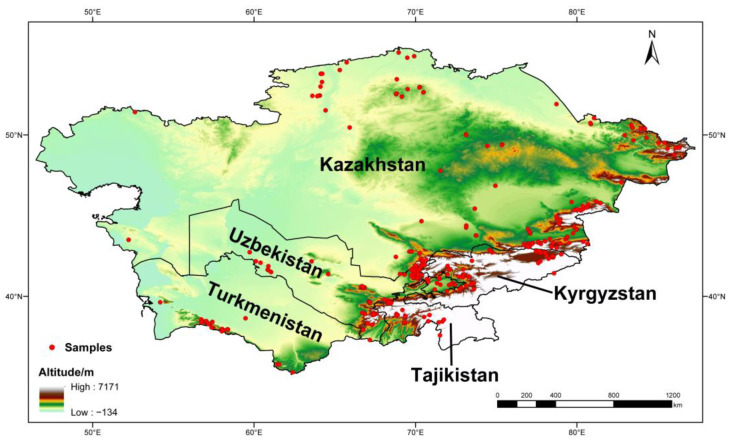
Known geographic distribution of Hymenochaetoid fungi indicated by the red points in Central Asia.

To avoid data redundancy of spatial autocorrelation, if sampling locations of occurrence records were distributed within 10 km, these records were treated as replications. After keeping only one record from replications, 412 of 762 occurrence records were filtered for predicting the geographic distribution of poroid Hymenochaetoid fungi in Central Asia (Figure 1).

Previous studies indicated that a serious multicollinearity problem exists among various bioclimatic variables [67,68]. Thus, to avoid over-fitting induced by the multicollinearity of variables, the Pearson correlation coefficient (r) analysis method was used to judge the correlation between primary environmental variables. When r > 0.8, two environmental variables were considered to be autocorrelated, and the one with higher contribution value was retained for further analyses based on the results of the pre-experiments (Figure S2). The contributions of environmental variables were judged by the jackknife cutting method. Eventually, 9 out of 21 environmental variables, viz., Bio2, Bio3, Bio7, Bio10, Bio12, Bio15, Bio17, Bio19, and host plant, were selected as the modeling factors.

The MaxEnt modeling was optimized by the R package “ENMeval v.2.0.4” [69]. Six regularization multiplier (RM) parameters were set from 0.5 to 3 with an interval of 0.5. The feature parameters linear (L), quadratic (Q), hinge (H), product (P), and threshold (T) are available in the MaxEnt modeling [70], and six feature combinations (FC), viz., L, LQ, H, LQH, LQHP, and LQHPT, were selected. A total of 36 parameter combinations from the above two kinds of parameters were tested with the function “ENMevaluate”. The fit and complexity of the modelings resulting from the 36 parameter combinations were evaluated by the Akaike information criterion correction (AICc) [71,72]. The parameter combination with a “delta AICc” value equal to zero was selected as the optimal parameter to predict the distribution. Of the 412 filtered records, 75% were randomly selected as training data, while the remaining 25% were used as testing data. The maximum number of iterations was set as 1000 to allow more time for convergence (threshold: 0.00001). To minimize the uncertainty associated with the random sampling and reduce the errors in the results, the process was repeated 10 times to generate an averaged result for subsequent analyses. A raster map of distribution probability was produced by MaxEnt, and the value of each grid cell indicated the occurrence probability as a floating-point format ranging from 0 to 1. The Jenks’ natural breaks method was used to divide the potential habitat into four levels following Zhao et al. [73]. The accuracy of the predicted geographic distribution was verified by the values of the area under receiver operator characteristic curve [74]. In theory, the model is considered to perform well when the value of AUC is more than 0.8 and excellently when the value is more than 0.9 [75]. The final map was generated by ArcGIS v.10.8 [66] based on the final prediction results from MaxEnt.

## 3. Results and Discussion

### 3.1. Annotated Checklist of Poroid Hymenochaetoid Fungi in Central Asia

**Figure 2:**
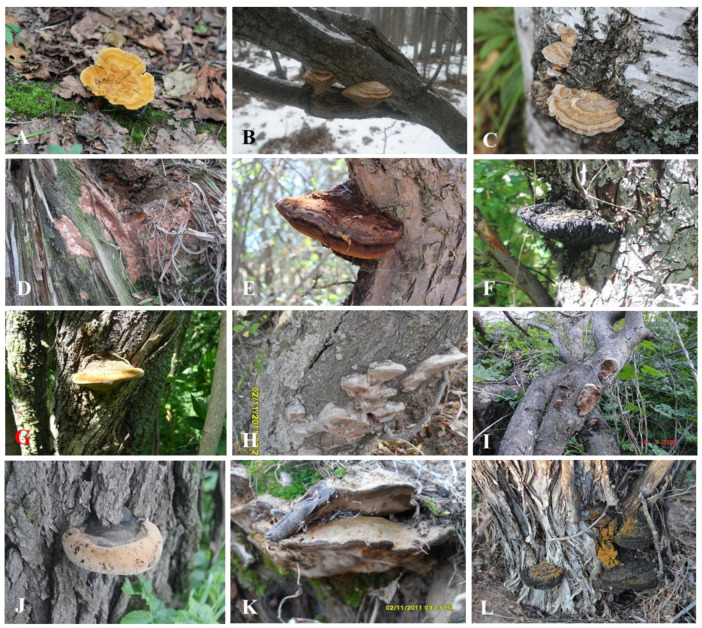
(A) *Coltricia perennis*; (B) *Fomitiporia hippophaeicola*; (C) *Inocutis rheades*; (D) *Coniferiporia uzbekistanensis*; (E) *Inonotus hispidus*; (F) *Inonotus obliquus*; (G) *Phellinopsis conchata*; (H) *Phellinus pomaceus*; (I) unidentified specimen; (J) *Phellinus igniarius*; (K) *Phylloporia yuchengii*; (L) *Sanghuangporus lonicerinus*. All photos credited to Yelena Rakhimova, Kanaim Bavlankulova, and Yusufjon Gafforov.

The annotated checklist of 43 species of wood-inhabiting poroid Hymenochaetoid fungi is arranged alphabetically by genera and species. The currency sign (¤) indicates new records to countries of Central Asia. Short notes are provided for some taxa. Photos of basidiomata in situ are shown for some species (Figure 2).

All abbreviations used in these localities are as follows: Five countries: Kazakhstan—KZ; Kyrgyzstan—KR; Tajikistan—TJ; Turkmenistan—TR; Uzbekistan—UZ; Provinces of five countries: KZ: Abai Province—ABP; Almaty Province—ALP; Aqmola Province—AQP; Atyrau Province—ATP; Kostanay Province—KTP; Karagandy Province—KRP; Zhambyl Province—ZMP; Turkestan Province—TKP; West Kazakhstan Province—WKP; Jetisu Province—JTP; East Kazakhstan Province—EKP; North Kazakhstan Province—NKP; Pavlodar Province—PVP, Big Almaty gorge—BAG, Small Almaty gorge—SAG. KR: Issyk-Kul Province—IKP; Jalal-Abad Province—JAP; Chuy Province—CYP. TJ: Gorno-Badakhshan Autonomous Province—GBP; Khatlon Province—KLP. UZ: Tashkent Province—TSP; Jizzakh Province—JZP; Republic of Karakalpakstan—RKK; Qashqadaryo Province—QDR; Namangan Province—NMP; Andijan Province—ADP; Surkhandaryo Province—SRP; Navoiy Province—NVP; Samarkand Province—SMP; Fergna Province—FRP; Syrdaryo Province—SDP. TR: Balkan Province—BKP; Ahal Province—AHP; Nature reserves: Ugam-Chatkal State Nature National Park—UCNNP; Chatkal Biosphere Reserve—CBR; Zarafshan State Reserve—ZSR; Hissar State Nature Reserve—HSNR; Zaamin National Park—ZNP; Zomin Mountain-Forest State Reserve—ZFSP; Surkhan State Reserve—SSR; Lower-Amudarya Biosphere Reserve—LBR; Nurata State Reserve—NSR; Issyk Kul Biosphere Reserve—IBR; Sary-Chelek Nature Reserve—SCNR; nature areas: Pamir-Alay Mountain System—PAMS; Western Tien Shan Mountains—WTSM; Tien Shan Mountains—TSM; Central Tien Shan Mountains—CTSM; Trans-Ili Alatau—TLA; Dzungarian Alatau—DGA; Pamir Mountains—PMts; Chatkal Mountain Range—CMR; Kurama Mountain Range—KMR; Ugam Mountain Range—UMR; Pskem Mountain Range—PMR; Hissar Mountain Range—HMR; Turkestan Mountain Range—TMR; Kopet-Dagh Mountains—KDM; Fergana Range—FRG; Nurata Range—NRG; Forestry—FY; River—RVR; Gorge—GRG; District—DT; Village—VLG; Valley—VLY; Range—RD; River—RVR; 

Kingdom: Fungi

Phylum: Basidiomycota

Class: Agaricomycetes

Order: Hymenochaetales

Family: Hymenochaetaceae

***GENUS: COLTRICIA***

***C. perennis*** (L.) Murrill.—on dried conifer tree: KZ, KTP, Lake Borovskiy, 1 Aug., 1946 [22], Borovskiy FY, 30 Jul. 1960 [22], Arakarayskiy FY, Krasniy gordon, 25 Aug. 1960 [22], Amankaragayskiy FY, 17 Jul. 1953 [22], *ibid*., 26 Aug. 1960 [22], *ibid*., 31 Aug. 1960 [22], Presnogorkovki VLG, 21 Aug. 1960 [22], *ibid.*, 21 Aug. 1960 [22]; AQP, Sandiktavskiy FY, 6 Jul. 1953 [22]; KRP, Karakalinska, 23 Jul. 1953 [22], South Janavul, 9 Jul. 1954 [22], Besaba VLG, 12 Jul. 1954 [22].

***GENUS***: ***CONIFERIPORIA***

***C. uzbekistanensis*** L.W. Zhou, Xue W. Wang & Gafforov—on *Juniperus seravschanica* Kom. (Cupressaceae) Kitam., UZ: JZP, Zaamin DT, ZNP, ZFSP, TMR, PAMS, 9 Sep. 2016 [33], *ibid*., 9 Sep. 2016, YG1018; *ibid*., 10 Sep. 2017, YG1105.

***C. weirii*** (Murrill) L.W. Zhou & Y.C. Dai—on *Juniperus seravschanica*, TJ: Varzob DT, PAMS, Hissar Range, Kondora VLY, Kvak, 27 May 1978, TAAM097356.

***GENUS***: ***FOMITIPORIA***

***F. hartigii*** (Allesch. & Schnabl) Fiasson & Niemelä [≡*Phellinus hartigii* (Allesch. & Schnabl) Pat.]—on *Abies sibirica* Ledeb. (Pinaceae), KZ: WKP, Liningorskiy FY, GRG Sakmarix, 30 Jul. 1947 [22], *ibid.* 2 Aug. 1947 [22], Liningorskiy FY, Ulbinka Mts., 17 Jul. 1961, [22].

***F. hippophaeicola*** (H. Jahn) Fiasson and Niemelä [≡*Phellinus hippophaeicola* H. Jahn].—on *Betula tianschanica* Rupr. (Betulaceae): KR: IKP, IBR, CTSM, 2021 [22].—on *Hippophae rhamnoides* L. (Elaeagnaceae), KZ: ALP, Usik RVR, Usikskaya FY, 30 Mar. 1943 [22], Karasay DT, TLA, BAG, Alma-Arasan resort, 20 May 1942 [22], SAG, Kazak RVR, 5 Sep. 1950 [22]; KR: JAP, FRG, TSM, 2023 [18]; IKP, Issikul DT., IBR, 2012 [76]. TJ: GBP, Pamir, Vantsh, Gudshevash, Vanj, 8 Jun. 1971, TAAM097356, *ibid*., 8 Jun. 1979, TAAM097358, Lyuli-Harvi, Peter the First Range of PMts., 18 Jun. 1979, TAAM115069; UZ: TSP, UCNNP, WTSM, 26 Apr. 1989 [15], Parkent DT, Nivich and Qiziljar VLGs, Bashkyzylsay, CBR, CMR, WTSM, 3 Sep. 1989 [15]; SMP, ZSR, Zarafshan VLY, PMts., 3 Oct. 1989 [15].

***Note***: New record for Tajikistan.

***F. punctata*** (P. Karst.) Murrill [≡ *Phellinus punctatus* (Fr.) Pil.]—on *Betula* sp. (Betulaceae): UZ: JZP, NSR, NRG, PAMS, 21 Jul. 1989 [15].—on *Crataegus* sp. (Rosaceae): UZ: TSP, Parkent DT, CBR, CMR, WTSM, 7 Aug. 1989 [15].—on *Crataegus azarolus* var. *pontica* (K. Koch) K.I. Chr. (Rosaceae): UZ: TSP, Parkent DT, UCNNP, WTSM, 17 Apr. 1988 [15].—on *Crataegus songarica* K. Koch (Rosaceae): KZ: ALP, Lepsinka, DGA, 8 Jun. 1944 [22], *ibid*., 12 Jun. 1944 [22]; JTP, Aksu DT, Mountain range, DGA [19].—on *Crataegus sanguinea* Pall. (Rosaceae): KZ: EKP, Aul, 15 Sep. 1943 [22].—on *Populus* sp. (Salicaceae): UZ: SMP, ZSR, Zarafshan VLY, PMts., 16 Apr. 1987 [15]; JZP, Zaamin DT, ZNP in the south and southeast of the TMR, 9 Jul. 1987 [15]; RKK, LBR, 21 Mar. 1987 [15].—on *Salix* sp. (Salicaceae): KZ: EKP, Belyagachskiy FY, 1 Sep. 1943 [22], *ibid*., 10 Sep. 1943 [22].—on *Salix wilhelmsiana* M. Bieb. (Salicaceae): KZ: ALP, Uygur DT, Charyn, 8 Oct. 1941 [22].—on *Ulmus* sp. (Ulmaceae): UZ: TSP, UCNNP, WTSM, 12 May 1988 [15]; QDP, HSNR in northwestern of HMR, PAMS, 14 Aug. 1987 [15].

***F. robusta*** (P. Karst) Fiasson & Niemela [≡ *Phellinus robustus* (P. Karst.) Bondartsev et Galzin].—on *Atraphaxis pyrifolia* Bunge (Polygonaceae): TJ: Mountain, (Schwartzman, [22] as *Phellinus robustus* f. *atrophaxidis* Bondartsev).—on *Castanea* sp. (Fagaceae): UZ: JZP, NSR, NRG, PAMS, 19 May 1987 [15]; QDR, HSNR in northwestern of Hissar Range, PAMS, 4 Mar. 1988 [15].—on *Hippophae rhamnoides* (Elaeagnaceae): KZ: ALP, Karasay DT, BAG, 2023 [19]; KR: TSM, [25], JAP, FRG, TSM, 2021 [22]; Uzgen DT, Tugay forest, Mirzaki VLG, 25 Aug. 1999 [77]; CYP, Bishkent city, Botanical Garden 2022, [18]; IKP, IBR, 2012 [17], *ibit*. 2021 [22].—on *Juglans regia* L. (Juglandaceae): UZ: NMP, Turaqurgon DT, Kuymazor VLG, Pop and Chust foothills, Apr. 2001 [15].—on *Morus alba* L. (Moraceae): UZ: NMP, Turaqurgon DT, Kuymazor VLG, Pop and Chust foothills, 2 Apr. 2001 [15].—on *Picea schrenkiana* Fisch. & C.A. Mey. (Pinaceae): KR: IKP, IBR, CTSM, 2021 [22].—on *Pistacia* sp. (Anacardiaceae): UZ: TSP, Parkent DT, CBR, CMR, WTSM, 7 Apr. 198 [15]; QDR, Yakkaobod VLG, Yakkabog FY, 7 Apr. 1988 [15].—on *Populus* sp. (Salicaceae): UZ: JZP, Zaamin DT, ZNP, ZFSP in the south and southeast of the TMR, 19 Aug. 1988 [15].—on *Quercus* sp. (Fagaceae): UZ: TSP, UCNNP, WTSM, 24 Apr. 1987 [15], SMP, ZSR, Zarafshan VLY, PMts., 14 Mar. 1987, [15].—on *Salix wilhelmsiana* (Salicaceae): KZ: ALP, Uygur DT, Charyn, 15 Oct. 1941 [22], *ibid*., 2023 [19].—on *Spiraea* sp. (Rosaceae): UZ: TSP, UCNNP, WTSM [15].—on *Spiraea crenata* L. (Rosaceae): KZ: KTP, Semiozyorniy DT, 10 May 1946 (Schwartzman, [22] as *Phellinus robustus* f. *spiraeae* Bondartsev), WKP, Buxtarminskoy sea, 20 Jul. 1961 (Schwartzman, [22] as *Phellinus robustus* f. *spiraeae* Bondartsev).—on *Spiraea hypericifolia* L. (Rosaceae): KZ: KRP, Zhanaarka DT, Aktau, 8 Aug. 1958 (Schwartzman, [22] as *Phellinus robustus* f. *spiraeae* Bondartsev); AQP, Kokchatau, 29 Jun. 1957 [22]; WKP, Katon-Karasayskiy FY, Enbek VLG, 20 Jun. 1961 (Schwartzman, [22] as *Phellinus robustus* f. *spiraeae* Bondartsev).—on trunk of angiosperm wood: KR: FRG, TSM [25].

***Note***: New record for Tajikistan.

***GENUS***: ***FULVIFOMES***

***F. kravtzevii*** (Schwarzman) Y.C. Dai & F. Wu [≡*Phellinus kravtzevii* Schwarzman]—on *Calligonum aphyllum* (Pall.) Gürke (Polygonaceae): KZ: ALP, Moiynkum Desert, 1940–1945 [22]; ZMP, Chu RVR, Koskudydkaya FY, [22], Karachardinskaya FY, 10 Oct. 1942, [22].

***F. rimosus*** (Berk.) Fiasson and Niemelä [≡*Phellinus rimosus* (Berk.) Pilát]—on *Pistacia vera* L. (Anacardiaceae): KZ: TKP, RVR Bolshoy Borolday, 23 Aug. 1960 [22]; KR: JAP, Arslanbob, FRG, TSM, Aug. 1937 [18], Bazar-Korgon DT, Karabulak VLG, Tooskol-Atinskiy FY, 6 Aug. 1998 [77] FRG, TSM, 2023 [18]; UZ: TP, UCNNP, WTSM, 30 Jul. 1963 [15].—on *Quercus* sp. (Fagaceae): UZ: TSP, 30 Jul. 1985 [15]; RKK, LBR, 6 Jul. 1985 [15]; QDR, HSNR in northwestern of HMR, PAMS, 30 Jul. 1985 [15].—on *Salix* sp. (Salicaceae): UZ: TSP, Parkent DT, CBR, CMR, WTSM, 25 Apr. 1986 [15]; SMP, ZSR, Zarafshan VLY, PMts., 12 Jul. 1985 [15].—on *Populus* sp. (Salicaceae): UZ: JZP, Zaamin DT, ZNP, ZFSP in the south and southeast of the TMR, 19 Aug. 1989 [15]; QDR, HSNR in northwestern of HMR, 10 Apr. 1989 [15].

***F. robiniae*** (Murrill) Murrill. [≡*Phellinus robiniae* (Murrill) A. Ames]—on *Pistacia vera* L. (Anacardiaceae): TJ: Varzob DT, Kondora VLY, 5 Apr.1977, TAAM096741, KLP, RVR Vakhsh, Nurak, 25 Apr. 1980, TAAM208173, TAAM102813; TR: Mary DT, Kolodets Akarcheshme, 21 May 1972, TAAM055757, TAAM055772, *ibid*., 22 May 1972, TAAM055771, TAAM055773, TAAM055769, KDM, Akarcheshme spring, 22 Apr. 1972, TAAM055755, TAAM055760, TAAM055761, TAAM055762, TAAM055763, TAAM055764, Mary DT, Kuhska, 24 Apr. 1972, TAAM055819, TAAM055818, TAAM208172, TAAM055828, Ahal DT, Kopet-Dagh, Kushka, 24 Apr. 1972, TAAM055822, Mary DT, KDM, by RVR Kushka, 24 Apr. 1972, TAAM055821, Kolodets Akar-Cheshme, Badgyz Nature Reserve, 20 Apr. 1972, TAAM055799, *ibid*., 21 Apr. 1972, TAAM208174, TAAM055758, TAAM055765, TAAM055768, TAAM055767, TAAM055766, TAAM055770, TAAM208171, TAAM055756, *ibid*., 22 Apr. 1972, TAAM055754, *ibid*., 24 Apr. 1972, TAAM055827, TAAM055759, Penkha-Cheshme, 7 Apr. 1971, TAAM079464.

***Note***: New record for Tajikistan and Turkmenistan.

***GENUS***: ***FUSCOPORIA***

***F. contigua*** (Pers.) G. Cunn. [≡*Phellinus contiguus* (Pers.) Pat.]—on *Acacia* sp. (Fabaceae): UZ: TSP, Parkent DT, CBR, CMR, WTSM, 27 Aug. 1987 [15], *ibid*., 6 Sep. 1987 [15].—on *Alnus* sp. (Betulaceae): UZ: JZP, NSR, NRG, PAMS, 16 Aug. 1988 [15]; QDP, HSNR in northwestern of HMR, PAMS, 23 Sep. 1987 [15].—on *Hippophae rhamnoides* (Elaeagnaceae): UZ: SMP, ZSR, Zarafshan VLY, PMts., 19 Sep. 1986 [15].—on *Populus tremula* L. (Salicaceae): KZ: ALP, SAG, TLA, 17 Aug. 1948 [22].—on *Ulmus* sp. (Ulmaceae): UZ: JZP, Zaamin DT, ZNP, ZFSP in the south and southeast of the TMR, 19 Sep. 1988 [15]; PKK, LBR, 30 Aug. 1989 [15].

***F. ferruginosa*** (Schrad.) Murrill [≡*Phellinus ferruginosus* (Schrad.) Pat.]—on *Crataegus sanguinea* Pall. (Rosaceae): KZ: EKP, Baradulskiy DT, Krasnoauliskaya country-house, 20 Sep. 1943 (Schwartzman, 1964).—on *Frangula alnus* Mill. (Rhamnaceae): KZ: EKP, Baradulskiy DT, Krasnoauliskaya country-house, 10 Sep. 1943 [22].—on *Juglans regia* (Juglandaceae): TJ: Varzob DT, Kondora VLY S-slope, HMR, PAMS, 25 May 1978, TAAM097241.—on *Salix caprea* L. (Salicaceae): KZ: EKP, Katan-Karagay, Urilya VLG, 28 Jul. 1961 [22].—on *Salix caprea* L. (Salicaceae): KZ: EKP, Krasnoauliskaya dacha, 10 Sep. 1943 [22].—on *Salix turczaninowii* Laksch. (Salicaceae): KZ: EKP, Katan-Karagay, Urilya VLG, 28 Jul. 1961 [22].

***Note***: New record for Tajikistan.

***F. torulosa*** (Pers.) T. Wagner and M. Fisch. [≡*Phellinus torulosus* (Pers.) Bourdot et Galzin]—on *Betula tianschanica* (Betulaceae): UZ: ADP, Garden and Parks of Andijan city, Sep.–Oct. 1999–2003 [15].—on *Celtis caucasica* Willd. (Cannabaceae): KZ: TKP, Boroldaytau, 12 Aug. 1959 [22].—on *Crataegus chlorocarpa* Lenné & K. Koch (Rosaceae): KZ: WKP, Ust-yazavoy VLG, 19 Jul. 1959 [22].—on *Fraxinus sogdiana* Bunge (Oleaceae): KZ: ALP, Talgar DT, TLA, Talgar GRG, [19], Talgorskiy ushelya, 20 Sep. 1935 [22], Uygur DT, Charyn, 5 Oct. 1941 [22], *ibid*., 15 Oct. 1941 [22], *ibid*., 12 Oct. 1941 [22], *ibid*., 15 Mar. 1943 [22].—on *Malus* sp.- (Rosaceae): KR: JAP, FRG, TSM, 2023 [18].—on *Malus domestica* (Suckow) Borkh. (Rosaceae): KZ: JTP, Eskeldi DT, TLA [19], Tekeli city, 11 Sep. 1960 [22]. KR: JAP, Bazar-Korgon DT, FRG, Yarodar GRG, Apsanbap-Atinskiy FY, 18 Sep. 1998 [77].—on *Malus sieversii* (Ledeb.) M. Roem. (Rosaceae): KZ: JTP, Eskeldi DT, TLA (Rakhimova et al. 2023).—on *Morus nigra* L. (Moraceae): UZ: ADP, Garden and Parks, Sep.–Oct. 1999–2003 [15].—on *Prunus* sp. (Rosaceae): KR: JAP, FRG, TSM, 2023 [18].—on *Prunus cerasifera* Ehrh. (Rosaceae): KR: JAP, Bazar-Korgon DT, FRG, Yarodar GRG, Apsanbap-Atinskiy FY, 18 Sep. 1998 [77].—on *Pyrus communis* L. (Rosaceae): UZ: ADP, Garden and Parks, Sep.–Oct. 1999–2003 [15].—on *Quercus* sp. (Fagaceae): UZ: Tashkent Botanical Garden, Jun. 1986 [15], *ibid*., Sep. 1987 [15].—on *Salix babylonica* L. (Salicaceae): UZ: ADP, Garden and Parks of Andijan city, Sep.–Oct. 1999–2003 [15].—on *Salix wilhelmsiana* (Salicaceae): KZ: LP, Uygur DT, Charyn [19].

***GENUS***: ***HIRSCHIOPORUS***

***H. abietinus*** (Pers. ex J.F. Gmel.) Donk [≡ *Trichaptum* abietinum (Pers. ex J.F. Gmel.) Ryvarden]—on *Abies sibirica* Ledeb. (Pinaceae): KZ: WKP, Pidder FY, Sakmarixa GRG, 5 Jul. 1947 [22], *ibid*., 12 Jul. 1947 [22], Juravlixinskaya FY, 15 May 1953 [22], Tolstuxa VLG, 1 Jul. 1951 [22], Lininogorskiy FY, 20 Jul. 1953 [22]; UZ: TSP, UCNNP, WTSM, 29 Aug. 1958, TAAM009360 [15].—on *Picea schrenkiana* Fisch. & Mey. (Pinaceae): KZ: ALP, Almaty, Medeo, 2 May 1984, TAAM105672, SAG, GRG Ksil, TLA, [19], SAG, RVR Baytereyke, TLA, 7 Oct. 1945 [22], RVR SAG, 17 Aug. 1948 [22], *ibid*., 18 Aug. 1948 [22], GRG, Orlinye, 17 Sep. 1957 [22], DGA, RVR Big Baskan, 27 May 1944 [22]; KR: IKP, Tien-Schan interior, Terskei Alatoo Mountains, Tschon-Kyzyl-Suu, 3 Jun. 1971, TAAM065067, *ibid*., 5 Jun. 1971, TAAM065125, 6 Jun. 1971, TAAM065165, TAAM065174, TAAM065185, Terskey Alatau Mts., Tossor RVR, 10 Jun. 1971, TAAM065247, Tianschan interior, Montes Terskei Alatau, 6 Jun. 1971, TAAM205758.—on *Pinus* sp. (Pinaceae): UZ: QDP, HSNR in northwestern of HMR, PAMS, 9 Jul. 1989 [15]; TSP, Parkent DT, CBR, CMR, WTSM, 17 Aug. 1990 [15], *ibit*., 12 Mar. 1988 [15]; JZP, NSR, NRG, PAMS, 15 Aug. 1988 [15].—on *Pinus sylvestris* L. (Pinaceae): KZ: AQP, Makinsk, Bulandskiy FY, 10 Jul. 1941 [22], Sandiktavskiy FY, 27 Jul. 1954 [22], KTP, Semozernoy FY, 12 Oct. 1944, [22]; KR: IKP, Tien-Shan, Terskey Alatau Mts., Tepleklyuchenka, 24 Aug. 1965, TAAM044050.—on *Pinus sibirica* Du Tour (Pinaceae): KZ: WKP, Belyagachskiy FY, 1 Sep. 1943 [22], Katon Karagay, Urylya VLG, 20 Jul. 1961 [22].—on *Salix caprea* L. (Salicaceae): KZ: WKP, Belyagachskiy FY, 1 Oct. 1943 [22].

***Note***: New record for Kyrgyzstan.

***H. fuscoviolaceus*** (Ehrenb.) Donk [≡ *Trichaptum fuscoviolaceum* (Ehrenb.) Ryvarden].—on *Abies sibirica* Ledeb. (Pinaceae): KZ: EKP, Lininogorskiy FY, 20 Jul. 1953 [22].—on *Pinus sibirica* Du Tour (Pinaceae): KZ: EKP, Katon Karagay, Urylya VLG, 20 Jul. 1961 [22].—on *Pinus sylvestris* L. (Pinaceae): KZ: KTP, Semozernoy FY, 12 Oct. 1944 [22].

***H. tianschanicus*** Y.C. Dai, Yuan & Meng Zhou—on *Larix* sp. (Pinaceae): KR: TSM, 15 Sep. 2016, KA16-1050 [61].—on *Picea* sp. (Pinaceae): KR: TSM, 15 Sep. 2016 [61].

***GENUS***: ***INOCUTIS.***

***I. dryophila*** (Berk.) Fiasson & Niemelä [≡ *Inonotus dryophilus* (Berk.) Murrill]—on *Acer negundo* L. (Sapindaceae): TR: Ashabad DT Ashgabat, Firjuza passage, 4 Apr. 1969, TAAM033003, *ibid*., 3 Apr. 1969, TAAM033006.

***Note***: New record for Turkmenistan.

***I. rheades*** (Pers.) Fiasson & Niemela Donk [≡ *Inonotus rheades* (Pers.) Bond. et Sing.]—on *Betula pubescens* Ehrh. (Betulaceae): KZ: WKP, Borodulikha DT, Krasnoaulskaya FY, 16 Sep. 1943 [22].—on *Betula pendula* Roth (Betulaceae): KZ: ALP, north part of DGA, 12 Jul. 1962 [22]; JTP, Sarkant DT, Mountain range, northern spurs of DGA, [19].—on *Populus euphratica* Olivier (Salicaceae): KZ: ALP, Ile DT, Ile RVR, [19].—on *Populus tremula* L. (Salicaceae): KZ: KTP, Pervomayskiy FY, 18 Jun. 1960 [22], Borovskiy FY, 28 Jun. 1960 [22], Amankaraganskiy FY, 28 Aug. 1960 [22]; AQP, Sandiktavskiy FY, 31 Jun. 1954 [22], PVP, Urlyutyubskiy FY, 23 Aug. 1962 [22], WKP, Borodulikha DT, Krasnoaulskaya FY, 20 Oct. 1943 [22].

***I. tamaricis*** (Pat.) Fiasson and Niemelä [≡*Inonotus tamaricis* (Pat.) Maire]—on *Tamarix* sp. (Tamaricaceae): KZ: ALP, Ile DT, RVR Ile, near Ile VLG, 3 Feb. 1945, TAAM132377, near Myn-Arach VLG, 30 May 1958, TAAM132380, Charyn forest cabin, 6 Oct. 1940, TAAM132378, Talgar DT, RVR Charyn, 7 Oct. 1941, TAAM132375; ZMP, Kokterek DT, part Borohudzir, 28 May 1943, TAAM132381; TR: Kara-Kala DT Co., KDM, 23 Apr. 1971, TAAM055060, *ibid*., 23 Apr. 1971, TAAM055061, Kara-Kala, experimental station, 9 Apr. 1969, TAAM033036, Ashgabat, 15 Apr. 1971, TAAM054914, Molla-Kara, 5 May 1961 TAAM207793; UZ: SRP, SSR, 06 May 1987 [15]; RKK, LBR, 24 Sep. 1986 [15].—on *Tamarix hispida* Willd. (Tamaricaceae): UZ: SMP, ZSR, Zarafshan VLY, PMts., 31 Sep. 1987 [15]; NVP, Kyzyl-kum Desert, 18 Mar. 1988 [15].—on *Tamarix pallasii* Desv. (Tamaricaceae): KZ: ALP, Uigurski DT, Yasenevskaya Forest, 12 Nov. 1941, TAAM205044.—on *Tamarix ramosissima* Ledeb. (Tamaricaceae): KZ: ALP, Qonayev, RVR Ile, [19], Charyn forest cabin, 12 Oct. 1941 [22], Ile VLG, 15 Mar. 1943 [22], *ibid*., 3 Feb. 1943, [22]; ZMP, RVR Chu, 6 Oct. 1940 [22], Min aral, 30 May 1958, [22]; UZ: NVP, Kyzyl-kum Desert, 27 Aug. 1989 [15], *ibid*., 1992–1993 [15].

***Note***: New record for Turkmenistan.

***GENUS***: ***INONOTUS***

***I. andersonii*** (Ellis and Everh.) Cerný.—on *Quercus* sp. (Fagaceae): UZ: TSP, Parkent DT, CBR, CMR, WTSM, 14 Aug. 1988 [15], *ibid*., 2 Sep. 1989 [15]; QDR, HSNR in northwestern of HMR, PAMS, 16 Sep. 1988 [15].

***I. cuticularis*** (Bull.) P. Karst.—on *Juglans regia* L. (Juglandaceae): UZ: QDR, HSNR in northwestern of HMR, PAMS, 10 Jul. 1990 [15].

***I. hispidus*** (Bull.) P. Karst.—on *Acer* sp (Sapindaceae): KR: JAP, walnut FY, Arslanbob, 2001 [77]; CYP, Bishkek city, Botanical Garden [18]—on *Asclepias syriaca* L. (Apocynaceae): TR: Bakharden DT, KDM, Nuchur, Kara-Suv, 20 Oct. 1971, TAAM055495.—on *Celtis caucasica* Willd. (Cannabaceae): KZ: ATP, Mangyshlak track, Sarybala, 23 Aug. 1960 [22].—on *Fraxinus sogdiana* Bunge (Oleaceae): KZ: ALP, Almaty city, Oct. 1935 [22].—on *Juglans regia* L. (Juglandaceae): KZ: TKP, UMR, WTSM, 10 Oct. 1948, [22], PMR, WTSM, 1 Aug. 1950 [22]; KR: JAP, Aksu DT, Arkyt VLG, SCNR, 1968 [78], North Kyrgyzstan 1968 [24], JAP, FRG, TSM, [18]; UZ: TSP, Bustonliq DT, Xumson VLG, Xumsonsoy, UMR, WTSM, 26 May 2011 [15], Oqtosh VLG, UMR, WTSM, 6 Jun. 2011 [15], *ibid*., 11 Jun. 2014 [15], Yubileyniy VLG, Chimyonsoy, Chimgan, CMR, WTSM, 22 Apr. 1982 [15]; SRP, Baysun DT, Baysun VLG, Omonkhona, Baysun Mountain, southwestern spurs of the HMR in the western part of PAMS, 11 Aug. 2015 [15], Darband VLG, Baysun Mountain, southwestern spurs of the HMR in the western part of PAMS, 15 May 2016 [15].—on *Malus* sp. (Rosaceae): KR: JAP, Arslanbob walnut FY, FRG, TSM [18], *ibid*., 2023 [18].—on *Malus domestica* (Suckow) Borkh. (Rosaceae): KZ: ALP, Lepsinka, DGA, 10 Jun. 1944 [22], Qara-alma, DGA, 29 Aug. 1962 [22], near to Turgen VLG, DGA, 2 Oct. 1940 [22], *ibid*., 6 Aug. 1946 [22], SAG, 13 Aug. 1948 [22], Yunnat lake, 10 Jul. 1950 [22]; JTP, Aksu DT, DGA Mountain range, Tirekli RVR, Kara-alma GRG, 2023, [19], *ibid*., 2023, [19], Sarkant DT, surroundings of Lepsin VLG, 2023, [19]; UZ: All territories of Uzbekistan except Kyzyl-kum desert [15].—on *Malus sieversii* (Ledeb.) M. Roem. (Rosaceae): UZ: TSP, Bustonliq DT, Xojikent VLG, UMR, WTSM, 14 Sep. 2014 [15].—on *Morus alba* L. (Moraceae): KZ: ALP, Almaty city, 20 Jul. 1949 [22], *ibid*., 4 Aug. 1962 [22]; TR: Ashgabat, Tshuli VLG, 3 Apr. 1969, TAAM033012, TAAM033011; UZ: Tashkent city, olimlar shaxarchasi, 17 Sep. 2015 [15]; NVP, Sarmysh VLY, 8 May 1976 [15].—on *Pinus* sp. (Pinaceae): UZ: TSP, Bustonliq DT, Onaulgansoy, Pskem RVR, PMR, WTSM, 19 Jun. 2014 [15], *ibit*., 19 Jun. 2014 [15].—on *Platanus orientalis* L. (Platanaceae): TR: Balkan, DT, KDM, Aydere, 10 Apr. 1969, TAAM033090.—on *Populus alba* L. (Salicaceae): KR: FRG, TSM [18].—on *Populus macrocarpa* (Schrenk) Pavlov & Lipsch. (Salicaceae): KZ: ALP, Almaty, Oct. 1935 [22].—on *Prunus avium* (L.) L. (Rosaceae): UZ: All territories of Uzbekistan except Kyzyl-kum desert [15].—on *Salix* sp. (Salicaceae): TR: Ashgabat, Firjuza passage, 3 Apr. 1969, TAAM033000, TAAM033008.—on *Tamarix* sp. (Tamaricaceae): KZ: TKP, station Arsy, Oct. 1935 [22].—on *Ulmus minor* subsp. *minor* (Ulmaceae): KZ: ALP, Almaty, 10 Sep. 1950 [22]; KRP, Balhash research station, 19 Aug. 1954 [22]. KR: JAP, Arslanbob walnut FY, FRG, TSM [18].—on trunk angiosperm wood, UZ: Tashkent city, olimlar shaxarchasi, 27 Sep. 2014 [15]; Tashkent Botanical Garden, 27 Sep. 2014 [15].

***Note***: New record for Turkmenistan.

***I. iliensis*** Kravtzev—on *Juglans regia* L. (Juglandaceae): KR: JAP, Arslanbob walnut FY, FRG, TSM, Jul. 1935 [79].—on *Morus alba* L. (Moraceae): KR: JAP, Arslanbob walnut FY, FRG, TSM, Jul 1938 [79].—on *Populus* sp. (Salicaceae): KZ: ALP, Qonayev city, Ile RVR, [19].—on *Populus euphratica* Olivier (Salicaceae): KZ: ALP, Uygur DT, Charyn ash tree forest, 10 Mar. 1943 [22].—on *Populus macrocarpa* (Schrenk) Pavlov & Lipsch. (Salicaceae): KZ: ALP, Uygur DT, Charyn ash tree forest, 15 Mar. 1943 [22].—on *Ulmus glabra* Huds. (Ulmaceae): KR: JAP, Arslanbob walnut FY, FRG, TSM, Jul 1938 [79].

***I. obliquus*** (Ach. ex Pers.) Pilat.—on *Alnus* sp. (Betulaceae): UZ: JZP, NSR, NRG, PAMS, 9 Jul. 1986 [15].—on *Betula* sp. (Betulaceae): UZ: TSP, Parkent DT, CBR, CMR, WTSM, 28 Aug. 1987 [15].—on *Betula pendula* Roth (Betulaceae): KZ: KTP, Semozernoy FY, 8 Oct. 1944 [22], Borovskiy FY, 2 Aug. 1946 [22]; WKP, Belyagachskiy FY, 16 Sep. 1943 [22], Katon, 2023 [19]. *Fraxinus* sp. (Oleaceae): UZ: QP, HSNR in northwestern of HMR, PAMS, 21 May. 1986 [15].—on *Malus domestica* (Suckow) Borkh. (Rosaceae): KZ: ALP, Karasay DT, TLA, BAG, 2023 [19], Medeo DT, ridge between the RVR Small Almatinka and Butakovka, 2023 [19]; JTP, Aksu DT, DGA, RVR Tirekti, 2023 [19], Kerbulak DT, DGA, 2023 [19], Sarkant DT, DGA, Black RVR, 2023 [19].—on *Salix* sp. (Salicaceae): UZ: ADP, Garden and Parks, 29 Jun. 1989 [15]; JZP, Zaamin DT, ZNP, ZFSP in the south and southeast of the TMR, 13 Jun. 1987 [15]; RKK, LBR, 20 Apr. 1988 [15].—on trunk of angiosperm wood: KR: North Kyrgyzstan, TSM [24].—on angiosperm fallen trunk: UZ: Tashkent city, Tashkent Botanical Garden, 14 Oct. 2011 [15].

***I. pseudohispidus*** Kravtzev.—on *Populus macrocarpa* (Schrenk) Pavlov & Lipsch. ex Pavlov (Salicaceae): KZ: ALP, Uygur DT, Charyn, 2023 [19].—on *Fraxinus sogdiana* Bunge. (Oleaceae): KZ: ALP, Uygur DT, Charyn, 2023 [19].—on *Populus* sp. (Salicaceae): UZ: SRP, SSR, 18 Jul. 1988 [15], *ibit*., 26 Aug. 1989 [15]; RKK, LBR, 9 Aug. 1988 [15].—on *Populus alba* L. (Salicaceae): UZ: SMP, ZSR, Zarafshan VLY, PMts., 20 Jul. 1989 [15].—on *Populus euphratica* Oliv. (Salicaceae): UZ: RKK, LBR, 1956–1960 [15].—on *Populus euphratica* Oliv. (Salicaceae): UZ: RKK, LBR, 1956–1960 [15].

***GENUS***: ***MENSULARIA***

***M. radiata*** (Sowerby) Lázaro Ibiza [≡*Inonotus radiatus* (Sowerby) P. Karst.]. *Alnus* sp. (Betulaceae): UZ: FRP, Fergana city, 3 Jul. 1986 [15].—on *Prunus vularis* L. (Rosaceae): KZ: ALP, Almaty, 20 Sep. 1943 [22].—on *Quercus* sp. (Fagaceae): UZ: TSP, Parkent DT, CBR, CMR, WTSM, 7 Jul. 1987 [15]; QDP, HSNR in northwestern of HMR, PAMS, 17 Jun. 1987 [15].—on *Ulmus* sp. (Ulmaceae): UZ: JZP, Zaamin DT, ZNP, ZFSP in the south and southeast of the TMR, 17 Jun. 1989 [15]; SMP, ZSR, Zarafshan VLY, PMts., 20 Aug. 1989 [15].—on angiosperm wood: UZ: JZP, NSR, NRG, PAMS, 19 May 1987 [15].

***GENUS***: ***ONNIA***

***O. tomentosa*** (Fr.) P. Karst. [≡ *Polystictus tomentosus* (Fr.) Cooke].—on *Fraxinus* sp. (Oleaceae): KZ: KRP, Janauvul, 9 Jul. 1954 [22],—on conifer tree (Pinaceae): KZ: KRP, Janauvul, 9 Jul. 1954 [22].

***GENUS***: ***PALLIDOHIRSCHIOPORUS***

***P. biformis*** (Fr.) Y.C. Dai, Yuan Yuan & Meng Zhou [≡*Trichaptum pergamenum* (Fr.) G. Cunn.].—on *Betula pendula* Roth. (Butulaceae): KZ: KTP, Semozernoy FY, Amankaragayskaya FY dacha, 8 Oct. 1944 [22], Lake Borovskiy, 20 Jul. 1946 [22], Uzunkulskiy FY, 2 Aug. 1960 [22]; AQP, Makinsk, Bulandskiy FY, 16 Jul. 1946, [22], northwest. resort of Borovoy, 5 May. 1945 [22], Big Tyukty FY, 12 Aug. 1954 [22]; EKP, Lininogorskiy FY, 4 Aug. 1944 [22], Zyryanovskiy DT, 24 Aug. 1961 [22]; NKP, Pesnovskiy FY, 6 Aug. 1960 [22], *ibid*., 7 Aug. 1960 [22], Poludinskiy FY, 17 Aug. 1960 [22].—on *Populus* sp. (Salicaceae): UZ: TSP, Parkent DT, CBR, CMR, WTSM, 18 Jul. 1988 [15], RKK, LBR, 7 Sep. 1987 [15].—on *Salix* sp. (Salicaceae): UZ: SMP, ZSR, Zarafshan VLY, PMts., 15 Sep. 1987 (Gafforov et al. 2020), SRP, SSR, 18 Jul. 1988 [15].—on trunk of angiosperms tree: UZ: TSP, UCNNP, WTSM, 1989 [15], *ibid*., 1993 [15].

***GENUS***: ***PHELLINIDIUM***

***Ph. ferrugineofuscum*** (P. Karst.) Fiasson and Niemelä [≡*Phellinus ferrugineofuscus* (P. Karst.) Bourdot and Galzin]—on *Larix gmelinii* var. *gmelinii* (Pinaceae): TR: Tachta Bazar, 11 Aug. 1972, TAAM056071.—on *Picea* sp. (Pinaceae): UZ: QDP, Yakkaobod VLG, Yakkabog FY, 6 Nov. 1987 [15], HSNR in northwestern of HMR, PAMS, 28 Oct. 1987 [15],—on *Pinus* sp. (Pinaceae): UZ: JZP, Zaamin DT, ZNP, ZFSP in the south and southeast of the TMR, 24 Oct. 1988 [15]; TSP, Parkent DT, CBR, CMR, WTSM, 15 Sep. 1988 [15].

***Note***: New record for Turkmenistan.

***GENUS***: ***PHELLINOPSIS***

***Ph. conchata*** (Pers.) Y.C. Dai [≡ *Phellinus conchatus* (Pers.) Quél.]—on *Alnus* sp. (Betulaceae): UZ: FRP, Fergana, 29 Aug. 1989 [15]; QDP, HSNR in northwestern of HMR, PAMS, 8 Sep. 1989 [15].—on *Juniperus polycarpos* var. *turcomanica* (B. Fedtsch.) R.P. Adams (Cupressaceae): TR: Bacharden DT, The KDM, Aron, 17 Oct. 1971, TAAM055401.—on *Populus* sp. (Salicaceae): UZ: JZP, NSR, NRG, PAMS, 5 Oct. 1988 [15], SRP, SSR, 21 Oct. 1988 [15].—on *Populus tremula* L. (Salicaceae): KZ: ALP, Karasay DT, TLA, BAG, 14 Aug. 1948 [19].—on *Picea schrenkiana* Fisch. & C.A. Mey. (Pinaceae): KZ: ALP, Karasay DT, TLA, BAG, 28 Jul. 1948 [19].—on *Rosa* sp. (Rosaceae): TR: KDM, Aydere, 16 May 1969, TAAM060049.—on *Salix bebbiana* Sarg. (Salicaceae): KZ: JTP, Sarkant DT, DGA, Sarkand RVR, 2023, [19].—on *Salix capusii* Franch. (Salicaceae): KZ: ALP, Karasay DT, TLA, SAG, 2 Aug. 1948 [19], TLA, lesopitomnik agroselxoz, 14 Aug. 1948 [22].—on *Salix lanata* subsp. *lanata* (Salicaceae): KZ: ALP, Sarkanskiy FY, Small Baskan VLG, TLA, BAG, 28 May 1944 [22], Sarkanskiy FY, Katon-karagay GRG, TLA, 30 May 1944 [22], Sarkand RVR, TLA, 3 Jun. 1944 [22], TLA, Butovskiy GRG, RVR Bitovke, 15 Aug. 1948 [22].—on *Salix tenuijulis* Ledeb. (Salicaceae): KZ: ALP, Sarkand RVR, TLA, Big Almaty RVR, 20 May 1942 [22], Karasay DT, TLA, BAG, 2023 [19].—on *Salix triandra* L. (Salicaceae): KZ: ALP, Karasay DT, TLA, BAG, RVR Big Almaty, 19 May 1942 [22], Karasay DT, TLA, BAG, resort Alma-Arasan, 20 May 1942 [22], TLA, SAG, 12 Aug. 1942 [22], TLA, SAG, RVR Batareyki, 9 Aug. 1948 [22].—on *Syringa* sp. (Oleaceae): UZ: TSP, Parkent DT, CBR, CMR, WTSM, 10 Sep. 1988 [15].—on *Syringa vulgaris* L. (Oleaceae): KZ: ALP, Almaty, 9 Nov. 1949 [22], *ibid*., 20 Nov. 1949 [22].—on *Ulmus* sp. (Ulmaceae): UZ: SMP, ZSR, Zarafshan VLY, PMts., 13 Nov. 1988 [15], ibit., 13 Oct. 1988 [15].—on trunk of angiosperm wood: KR: TSM [24]; JAP, Arslanbob, FRG, TSM [77].

***Note***: New record for Turkmenistan.

***GENUS***: ***PHELLINUS***

***Ph. betulinus*** (Murrill) Parmasto.—on *Betula tianschanica* Rupr. (Betulaceae): UZ: TSP, Yangikurgan VLG, Kurigansay RVR, WTSM, 24 Apr. 1982, TAAM104436 [15], *ibid*., 24 Apr. 1982, TAAM104285 [15].

***Ph. igniarius*** (L.) Quél.—on *Acer* sp. (Sapindaceae): UZ: RKK, LBR, 12 Apr. 1987 [15]; NMP, Mingbuloq DT, Qorasuv garden (13), National Parks and Gardens, 19 Aug. 1987 [15].—on *Alnus glutinosa* (L.) Gaertn. (Betulaceae): TJ: GBP, Pamir, Horog, 1 Jun. 1978, TAAM198491.—on *Betula* sp. (Betulaceae): KR: JAP, Arslanbob walnut FY, FRG, TSM, Jul. 1935 [18].—on *Betula pubescens* Ehrh. (Betulaceae): KZ: ALP, Cherniy klyuch, north part of DGA, 12 Aug. 1962 [22]; AQP, Sandiktavskiy FY, 12 Aug. 1954 [22]; KTP, Borovskiy FY, 31 Jul. 1953 [22], Semozernoy FY, 12 Jul. 1953 [22].—on *Betula pendula* Roth (Betulaceae): KZ: ALP, Cherniy klyuch, north part of DGA, 13 Aug. 1962 [22]; AQP, Sandiktavskiy FY, 12 Aug. 1952 [22], *ibid*., 12 Aug. 1954, [22]; KTP, Borovskiy FY, 31 Jul. 1953 [22], Semiozernoy FY, 12 Jul. 1953 [22]; PVP, Chalday VLG, 20 Aug. 1941 [22]; ABP, Oqsaroy kordon, 17 Oct. 1955 [22], Aul, 25 Aug. 1943 [22].—on *Betula tianschanica* Rupr. (Betulaceae): KR: JAP, Mirzaki VLG, Arslanbob walnut FY, FRG, TSM, 25 Aug. 1999 [77], *ibid*., 2023 [18].—on *Juglans regia* L. (Juglandaceae) UZ: ADP, Garden and Parks, Apr.-May 2000 [15]; TSP, Bustonliq DT, Yubileyniy VLG, Chimyonsoy, Chimgan, CMR, WTSM, 1 Jun. 1980 [15], *ibid*., Sep. 1984 [15]; KR: JAP, FRG, TSM, 2023 [18], IKR, IBR, 2012 [17].—on *Picea schrenkiana* Fisch. & C.A. Mey. (Pinaceae): KR: IKP, IBR, CTSM, 2021 [22].—on *Populus alba* L. (Salicaceae): KZ: WKP, south slope of Narim ridge, RVR Topolev, 2 Jul. 1958 [22].—on *Prunus* sp. (Rosaceae): UZ: SMP, ZSR, Zarafshan VLY, PMts., 9 Aug. 1988 [15].—on *Prunus amygdalus* Batsch (Rosaceae): KR: CYP, Bishkek city, Botanical Garden, 2023 [18].—on *Prunus padus* L. (Rosaceae): KZ: ALP, Karasay DT, TLA, BAG, 15 May. 1942 [22], *ibid*., Karasay DT, BAG, 2023 [19], Enbekshikazakh DT, Batan VLG, TLA, 2023 [19].—on *Prunus spinosissima* (Bunge) Franch. (Rosaceae): KR: JAP, Arslanbob walnut FY, FRG, TSM, 25 Aug. 1999 [77].—on *Prunus vulgaris* L. (Rosaceae): UZ: TSP, Parkent DT, CBR, CMR, WTSM, 10 Sep. 1988 [15].—on *Salix* sp. (Salicaceae): UZ: TSP, Yangikurgan VLG, Kurigansay RVR, WTSM, 24 Apr. 1982 [15]; JZP, Zaamin DT, ZNP, ZFSP in the south and southeast of the TMR, 6 Apr. 1988 [15], Nurata DT, NSR, NRG, PAMS, 16 Jul. 1989 [15]; QDP, HSNR in northwestern of HMR, PAMS, 24 Jul. 1988 [15]; ALP, Karasay DT, Bolshaya Almatinka, 1 Jul. 1975, TAAM058566, TAAM058567; TJ: BMR, Pamir, Horog, 1 Jun. 1978, TAAM193670, TAAM097306, TAAM193671, TAAM193672, TAAM193673, TAAM193674, TAAM193675, TAAM193676, TAAM193677, Ramit State Nature Reserve, Hissar Mountain range in PMts., 12 Apr. 1977, TAAM096855, DTs of Republican Subordination, Miyonadu, Sauzehan, 24 Jun. 1979, TAAM115091, Peter the Great Range in Pamir Mountain System, 15 Jun. 1979, TAAM115015.—on *Salix acutifolia* Willd. (Salicaceae): KZ: ABP, Semipalatinskiy FY, Oqsaroy kordon, 17 Oct. 1955 [22].—on *Salix alba* L. (Salicaceae): KZ: WKP, Borili DT, Burli VLG, 27 Jun. 1952 [22], *ibid*., 1 Aug. 1952 [22].—on *Salix caprea* L. (Salicaceae): KZ: ALP, RVR Sarkand, DGA, 24 May. 1944 [22].—on *Salix songarica* Andersson (Salicaceae): KZ: ALP, Cherniy klyuch, north part of DGA, 20 Apr. 1943 [22].—on *Salix starkeana* Willd. (Salicaceae): KZ: ALP, Karasay DT, BAG, 2023 [19], Uygur DT, Ketmen Mountain Ridge, 2023 [19]; JTP, Kerbulak DT, DGA, hunting farm “Kumbel”, floodplain forest, 2023 [19], DGA, Taldibulak VLG, 2023 [19], BAG, resort Alma-Arasan, 20 May 1940 [22], DGA, Terekli VLG, 10 Jun. 1944 [22].—on *Salix tenuijulis* Ledeb. (Salicaceae): KZ: ABP, Targabataya, west VLG of Podgorno, 17 Aug. 1953 [22], RVR Usik, city Panfilova, 10 Apr. 1943 [22].—on *Salix triandra* L. (Salicaceae): KZ: ABP, Belyagachskiy FY dacha, 1 Sep. 1943 [22].—on *Salix turanica* Nasarow (Salicaceae): KZ: ALP, Chilik, Bortagoy, 14 Jun. 1962 [22], Charinskiy FY, RVR Charin, 15 Mar. 1943 [22].—on *Salix wilhelmsiana* M. Bieb. (Salicaceae): KZ: ALP, Charinskiy FY, 15 Oct. 1941 [22], *ibid*., 15 Apr. 1943 [22], *ibit*., 8 Apr. 1943 [22].—on *Ulmus laevis* Pall. (Ulmaceae): KZ: ALP, WKP, Borili DT, Gureev forest, 11 Jul. 1952 [22].—on trunk of angiosperm: KR: TSM, [24].

***Note***: New record for Tajikistan.

***Ph. nigricans*** (Fr.) P. Karst—on trunk of woody tree: KR: TSM, 27 Aug. 2018 [80], *ibid*., 14 Sep. 2016 [80], *ibid*., 9 Sep. 2017 [80], *ibid*., 28 Aug. 2016 [80], *ibid*., 16 Sep. 2016 [80].

***Ph. pomaceus*** (Pers.) Maire [≡*Phellinus tuberculosus* Niemelä]—on *Berberis turcomanica* Kar. ex Ledeb. (Betulaceae): TR: Central Kopet Dag, Jun. 1988, TAAM203625.—on *Celtis australis* subsp. *caucasica* (Cannabaceae): UZ: TSP, Parkent DT, CBR, CMR, WTSM, 1 May 1988 [15].—on *Crataegus chlorocarpa* Lenné & K. Koch. (Rosaceae): UZ: TSP, Parkent DT, CBR, CMR, WTSM, 02 May 1988 [15].—on *Cydonia oblonga* Mill. (Rosaceae): UZ: NMP, Pop DT, Chodaksay basin, KMR, WTSM, 1 May 2003 [15], *ibid*., 1 May 2003 [15].—on *Juglans regia* (Juglandaceae): KR: JAP, Sary-Chelek Nature Reserve, CMR, WTSM, 12 Aug. 1967, TAAM044710; UZ: TSP, Parkent DT, CBR, CMR, WTSM, 29 Apr. 1988 [15].—on *Lonicera* sp. (Caprifoliaceae): KR: JAP, Sary-Chelek Biosphere Reserve, 5 Aug. 1967, TAAM044612; UZ: TSP, Tuyatashsoy, WTSM, Sep. 1982 [15].—on *Malus* sp. (Rosaceae): KR: JAP, JAP, Sary-Chelek Nature Reserve, CMR, WTSM, 6 Aug. 1967, TAAM044399; UZ: JZP, Zaamin DT, ZNP, ZFSP in the south and southeast of the TMR, 16 May 1987 [15].—on *Malus domestica* (Rosaceae): UZ: ADP, Andijan DT, Kutarma VLG, May–Aug. 2002 [15], *ibid*., May–Aug. 2002 [15]; TSP, Parkent DT, CBR, CMR, WTSM, 13 [15].—on *Prunus* sp. (Rosaceae): KR: JAP, Aksyeskiy DT, SCNR, TSM [81]; JAP, FRG, TSM, 2021, [18]; TJ: DTs of Republican Subordination, Kondara, 26 Apr. 1980, TAAM102823, Takob, 12 Apr. 1977, TAAM097386, GBP, Gudshevash near Vanj, 8 Jun. 1978, TAAM097380; UZ: TP, Bustonliq DT, Burchmulla VLG, Kulabsay, WTSM, 26 Apr. 1982 [15], Oqtosh VLG, UMR, WTSM, 11 Sep. 2011 [15], *ibid*., 12 Sep. 2014 [15], *ibid*., 12 Sep. 2014 [15], Beldersay, Greater Chimgan, CMR, WTSM, 13 Sep. 2014 [15], Onaulgansoy, Pskem RVR, PMR, WTSM, 19 Sep. 2014 [15], UCNNP, WTSM, 10 May 1987 [15], *ibid*., 10 May 1987, [15], near RVR Chatkal and Kulyab-Say, 26 Apr. 1982, TAAM127415, stream Kulyab-saj, near Charvak Reservoir, 23 Apr. 1982, TAAM127401, TAAM127395, TAAM127399; JZP, Zaamin DT, ZNP, ZFSP in the south and southeast of the TMR, 9 Sep. 2016 [15]; QDP, HSNR in northwestern of HMR, PAMS, 26 May 1987 [15].—on *Prunus bucharica* (Korsh.) Hand. -Mazz. (Rosaceae): TJ: DT of Republican Subordination, 12 Apr. 1977, TAAM096857.—on *Prunus cerasifera* Ehrh (Rosaceae): KZ: TKP, UMR, WTSM, 10 Jun. 1949 [22], Karjantau Mountain, WTSM, 8 Jun. 1956 [22]; KR: JAP, Sary-Chelek Biosphere Reserve, 6 Aug. 1967, TAAM044397, TAAM044627, *ibid*., 8 Aug. 1967, TAAM044622; TAAM044635; TJ: DT of Republican Subordination, HMR, Kondora, PAMS, 7 Apr. 1977, TAAM096785, *ibid*., 5 Apr. 1977, TAAM096735, DT of Republican Subordination, 9 Apr. 1977, TAAM096810, *ibid*., 11 Apr. 1977, TAAM096834, TAAM096835, *ibid*., 12 Apr. 1977, TAAM096865, TAAM096867, TAAM096868, TAAM096869, TAAM096870; TR: BKP, Balkan DT, KDM, Aydere, 10 Apr. 1971, TAAM033051, *ibid*., 11 Apr. 1971, TAAM033060; UZ: TSP, Bustonliq DT, Kayinarsay and Sarvasay, WTSM, 31 Aug. 1963 [15], Oqtosh VLG, UMR, WTSM, 1 Jun. 2011 [15], Xojikent VLG, UMR, WTSM, 2 Nov. 2011 [15], *ibid*., 2 Nov. 2011 [15].—on *Prunus domestica* L. (Rosaceae): KZ: TKP, Talas Alatau, Aksu-Zhabagly Nature Reserve, 7 Jul. 1948 [22]; UZ: ADP, Andijan DT, Kutarma VLG, May–Aug. 2002 [15], *ibid*., May–Aug. 2002 [15], *ibid*., May–Aug. 2003 [15].—on *Prunus dulcis* (Mill.) D.A. Webb (Rosaceae): UZ: TSP, Bustonliq DT, Xojikent VLG, UMR, WTSM, 2 Nov. 2011 [15].—on *Prunus erythrocarpa* (Nevski) Gilli (Rosaceae): UZ: TSP, Yangikurgan VLG, Kurigansay RVR, WTSM, 24 Apr. 1982 [15].—on *Prunus griffithii* var*. tianshanica* (Pojark.) Ingram (Rosaceae): UZ: TSP, Bustonliq DT, Xojikent VLG, UMR, WTSM, 2 Nov. 2011 [15], *ibid*., 20 Sep. 2014 [15].—on *Prunus mahaleb* (Rosaceae): KZ: TKP, Karatau, Kukbulak, WTSM, 3 Aug. 1949 [22], Karjantau Mountain, WTSM, 11 Jun. 1956 [22]; UZ: TSP, Yangikurgan VLG, Kurigansay RVR, WTSM, 24 Apr. 1982 [15], Beldersay, Greater Chimgan, CMR, WTSM, 15 May 2011 [15].—on *Prunus microcarpa* C.A. Mey. (Rosaceae): TR: BKP, Balkan DT, KDM, Aydere, 18 Apr. 1971, TAAM054917, TAAM054948, *ibid*., 19 Apr. 1971, TAAM054921, *ibid*., 20 Apr. 1971, TAAM054984, TAAM054976, TAAM054940, Chozly-Dere, 22 Apr. 1971, TAAM055020, Mezat-Li in former Kara-Kala DT, 22 Apr. 1971, TAAM0549991.—on *Prunus persica* (L.) Batsch (Rosaceae): UZ: ADP, Andijan DT, Kutarma VLG, Apr. 2004 [15], *ibid*., Apr. 2004 [15].—on *Prunus spinosa* L. (Rosaceae): KZ: WKP, RVR Ural, Burlinskiy FY, Sep. 1956 [22]; UZ: ADP,—on *Salix* sp. (Salicaceae): UZ: TSP, Yangikurgan VLG, Kurigansay RVR, WTSM, 24 Sep. 2014 [15].—on *Prunus turcomanica* (Lincz.) Kitam (Rosaceae): TR: AHP, Ahal DT, surroundings of Nokhur, Kara-Suv stream, 20 Oct. 1971, TAAM055500, *ibid*., 20 Oct. 1971, TAAM055467, *ibid*., 21 Oct. 1971, TAAM055517.—on dried stem of angiosperm: UZ: TSP. Parkent DT, UCNNP, E of Parkent, 2 May 1988, TAAM126274, Bustonliq DT, Yubileyniy VLG, Chimyonsoy, Chimgan, CMR, WTSM, 26 Apr. 1982 [15].

***Note***: New record for Tajikistan and Turkmenistan.

***Ph. tremulae*** (Bondartsev) Bondartsev and P.N. Borisov—on *Populus* sp. (Salicaceae): UZ: FRP, Fergana city, 8 Aug. 1988 [15], *ibid*., Aug. 1988 [15]; TSP, UCNNP, WTSM, 20 Jul. 1985 [15]; JZP, NSR, NRG, PAMS, 25 Aug. 1986 [15].—on *Populus tremula* L. (Salicaceae): KZ: AQP, Otradenskiy FY, 4 Aug. 1953 [22]; KTP, Arakaragayskiy FY, 17 Aug. 1944, [22], Amankaragayskiy bor, 4 Oct. 1944 [22], Nauryzumskiy bor, 4 Oct. 1944, [22]; WKP, Juravlyovskiy FY, 25 Jul. 1947, [22], Katon karagay, Urilya VLG, 28 Jul. 1968, [22], Zyrnovskiya rayon, Bobrovki VLG, 29 Aug. 1961, [22]; UZ: TSP, Parkent DT, CBR, CMR, WTSM, 20 Jul. 1985 [15]; QDP, HSNR in northwestern of HMR, PAMS, 16 Aug. 1985 [15], SMP, ZSR, Zarafshan VLY, PMts., 10 Aug. 1986 [15]; SRP, SSR, 19 Aug. 1987, [15]; SDR, Sirdaryo, [15].

***GENUS***: ***PHYLLOPORIA***

***Ph. ampelina*** (Bondartsev and Singer) Bondartseva [≡ *Phellinus ampelinus* Bondartsev and Singer]—on *Vitis vinifera* L. (Salicaceae): UZ: TSP, UCNNP, WTSM, 1986 [15].

***Ph. ephedrae*** (Woron.) Parmasto—on *Crataegus* sp. (Rosaceae): TJ: KLP, Vahdat DT, Ramit Nature Reserve, 11 Apr. 1977, TAAM096826.—on *Crataegus pseudoheterophylla* subsp. *turkestanica* (Pojarkova) K.I.Chr. (Rosaceae): TR: AHP, Ahal DT, surroundings of Nokhur, Kara-Suv, 21 Oct. 2019, TAAM055515.—on *Crataegus* × *zangezura* nothosubsp. *pseudoambigua* (Pojark.) K.I.Chr. (Rosaceae): TR: BKP, Balkan DT, KDM, Aydere, 23 Oct. 2019, TAAM055527.—on *Chrysojasminum fruticans* (L.) Banfi (Oleaceae): TR: Kara-Kala DT, KDM, Iol-Dere, 25 Oct. 2019, TAAM055622, 25 Oct. 2019, TAAM055625.—on *Ephedra* sp. (Ephedraceae): KZ: ALP, Almaty DT, RVR Turgen, 3 May 1984, TAAM105676; TR: AHP, Bakharden DT, KDM, Nukhur, Kara-Suv, 20 Oct. 2019, TAAM055504, Ahal DT, Kök-Tepe region, KDM, in surroundings of Mount Dushak, 28 Oct. 1971, TAAM055669, *ibid*., 29 Oct. 1971, TAAM055674, TAAM055681, Shor-Gaudan, Firyusa VLY, 27 Apr. 1972, TAAM055736, Ashabad DT, KDM, Shor-Gaudan, 27 Apr. 1972, TAAM055705, TAAM055707; BKP, Balkan DT, KDM, Aydere, 22 Oct. 2019, TAAM055560; AHP, Ahal DT, surroundings of Nokhur, Kara-Suv, 21 Oct. 2019, TAAM055519, *ibid*., 22 Oct. 2019, TAAM055522.—on *Ephedra equisetina* Bunge (Ephedraceae): KZ: TKP, Sayram villiga, UMR, 20 Jun. 1959 (Schwartzman [22] as *Phellinus ribis* f. *ephedrae*-*nebrodensis* (Bourdot & Galzin) Pilát); UZ: TSP, Parkent DT, CBR, CMR, WTSM, 1 May 1988 [15], *ibid*., 1 May 1988 [15], *ibid*., 2 May 1988 [15].—on *Ephedra intermedia* Schrenk & C.A. Mey. (Ephedraceae): KZ: ALP, Uygur DT, Charyn, 20 May 1943 (Schwartzman [22] as *Phellinus ribis* f. *ephedrae*-*nebrodensis* (Bourdot & Galzin) Pilát); TR: BKP, Balkan DT, KDM, Aydere, 24 Oct. 1971, TAAM055384, TAAM055386, TAAM055388, TAAM055390, TAAM184270; AHP, Bakharden DT, KDM, Arvaz, 17 Oct. 1971, TAAM055471, TAAM055032, Kök-Tepe region, KDM, Mount Dushak, 29 Oct. 1971, TAAM166183, TAAM166184, Kara-Kala DT, KDM, Iol-Dere, 24 Oct. 2019, TAAM055530, TAAM055385, *ibit*., 25 Oct. 1971, TAAM055633.—on *Prunus bucharica* (Korsh.) Hand. -Mazz. (Rosaceae): TJ: Varzob DT, Hissar Mts., Takob, 15 Jun. 1978, TAAM097392.—on *Rosa* sp. (Rosaceae): TJ: Kondara Field Station (40 km N of Dushanbe), HMR, Kondora, PAMS, 6 Apr. 1977, TAAM096767, TAAM096727, *ibid*., 7 Apr. 1977, TAAM096784, TAAM096794, TAAM096779; GBP, Badakhshan, Kalai Humb, PMts., 12 Jun. 1978, TAAM097383.—on *Rosa canina* L. (Rosaceae): TR: Kara-Kala DT, KDM, Chalalgös, 23 Apr. 1971, TAAM055025.—on *Rosa × karakalensis* Kult. (Rosaceae): TR: BKP, Balkan DT, KDM, Aydere, 19 Apr. 1971, TAAM054954.

***Note***: New record for Tajikistan and Turkmenistan.

***Ph. pulla*** (Mont. & Berk.) Decock & Yombiy—нa *Berberis oblonga* (Rgl.) C.K. Schn. (Berberidaceae): KR: JAP, Chatkal DT, FRG, Ak-terek, 14 Jun. 1999 [77].

***Ph. ribis*** (Schumach.) Ryvarden—on *Berberis integerrima* Bunge (Betulaceae): KZ: ALP, Uygur DT, Charyn, 7 Apr. 1943 (Schwartzman [22] as *Phellinus ribis* f. *berberidis* Bondartsev), *ibit*., 15 Apr. 1943 (Schwartzman [22] as *Phellinus ribis* f. *berberidis* Bondartsev),—on *Berberis heteropoda* Schrenk ex Fisch. & C.A. Mey. (Betulaceae): KZ: ALP, TLA, Karaturskoe usheli, Chilikskaya FY, 12 Jul. 1949 (Schwartzman [22] as *Phellinus ribis* f. *berberidis* Bondartsev).—on *Berberis oblonga* (Rgl) Schneid. KR: JAP, Chatkal DT, Ak-terek walnut farmer, 7 Jun. 1938 [79]; IKP, Jety—Oguz DT, Barskoun mountain GRG, 25 Apr. 1956, Jety—Oguz mountain GRG, 29 Sep. 1956 [82].—on *Cotoneaster hissaricus* Pojark. (Rosaceae): TJ: Kondara Field Station (40 km N of Dushanbe), 6 Apr. 1977, TAAM096768.—on *Crataegus turkestanica* Pojark. (Rosaceae): TR: Bakharden DT, KDM, Nukhur, Kara-Suv, 21 Oct. 1971, TAAM055515.—on *Ephedra intermedia* Schrenk & C.A. Mey. (Ephedraceae): TR: Kara-Kala DT, KDM, Aydere, 24 Oct. 1971, TAAM055386.—on *Euonymus semenovii* Regel & Herder. (Celastraceae): KZ: ALP, TLA, SAG, Krestovaya mountain, 28 Aug. 1957 (Schwartzman [22] as *Phellinus ribis* f*. euonymi* (Kalchbr.) Pilát), Karasay DT, TLA, BAG, [22].—on *Rosa spinosissima* L. (Rosaceae): KZ: ALP, Uygur DT, Charyn, 5 Apr. 1943 (Schwartzman [22] as *Phellinus ribis* f*. rosae* (Jacz.) Pilát), *ibid*., 5 Apr. 1943 (Schwartzman [22] as *Phellinus ribis* f*. rosae* (Jacz.) Pilát).

***Note***: New record for Tajikistan and Turkmenistan.

***Ph. yuchengii*** Gafforov, Tomšovský, Langer and L.W. Zhou—on *Crataegus* sp. (Rosaceae): UZ: TSP, Bustonliq DT, Xojikent VLG, UMR, WTSM, 9 Oct. 2016 [15].—on *Crataegus pseudoheterophylla* subsp*. turkestanica* (Rosaceae): UZ: JZP, NSR, NRG, PAMS, 11 Sep. 2015 [15].—on *Juglans regia* L. (Juglandaceae): UZ: TSP, Bustonliq DT, Oqtosh VLG, UMR, WTSM, 1 Jun. 2011 [15], Parkent DT, CBR, CMR, WTSM, 29 Apr. 1988 [15].—on *Morus alba* L. (Moraceae): UZ: QDP, Yakkaobod VLG, Yakkabog FY, 13 Jun. 2013 [15], *ibid*., 13 Jun. 2013 [15].—on *Populus* sp. (Salicaceae): UZ: QDP, Yakkaobod VLG, Yakkabog FY, 12 Jun. 2013 [15].—on *Prunus* sp. (Rosaceae): UZ: TSP, Bustonliq DT, Xumson VLG, Xumsonsoy, UMR, WTSM, 11 Sep. 2011 [15].—on angiosperm trunk and stem: UZ: TSP, Bustonliq DT, Oqtosh VLG, UMR, WTSM, 1 Jun. 2011 [15], Xojikent VLG, UMR, WTSM, 2 Nov. 2011 [15], JZP, Zaamin DT, ZNP, ZFSP in the south and southeast of the TMR, 9 Sep. 2016 [15].

***GENUS***: ***PORODAEDALEA***

***P. chrysoloma*** (Fr.) Fiasson & Niemelä [≡ *Phellinus chrysoloma* (Fr.) Donk]—on *Picea schrenkiana* Fisch & C.A. Mey. (Pinaceae): KZ: ALP, Karasay DT, TLA, BAG, 2023, [19], SAG, 2023, [19], Talgar DT, TLA, Talgar GRG, 2023, [19]; KR: IKP, IBR, CTSM, [79], *ibid*., [82], *ibid*., [24], *ibid*., [22].

***P. pini*** (Brot.) Murrill, Bull. [≡ *Phellinus pini* (Brot.) Pilát]—on *Abies sibirica* Ledeb. (Pinaceae): KZ: EKP, Juravlexskaya FY, 4 Aug. 1947 [22], Katon-Karasayskiy FY, Urilya VLG, 25 Jul. 1961 [22], *ibid*., 26 Jul. 1961 [22].—on *Picea schrenkiana* Fisch & C.A. Mey. (Pinaceae): KZ: ALP, Kegen DT, Kungey Alatau ridge, Saty RVR, 2023, [19], *ibid*., 26 Jul. 1961 [22], TLA, 20 Aug. 1950 [22], Kegen DT, Tauchilskaya FY, 16 Jul. 1952 [22], *ibid*., 15 Oct. 1941 [22], *ibid*., 20 Oct. 1941 [22], TLA, SAG, 1934 [22], RVR Small Almaty, 17 Aug. 1948 [22], *ibid*., 18 Aug. 1948 [22], BAG, 1947 [22], BAG, near to lake, 10 Sep. 1953 [22], Batareyka (Bedelbay) RVR, 28 Jul. 1951 [22], Ketmen ridge in Zailiisky Alatau, northern TSM, 20 May 1943 [22], Besik-Aktash ushyli, 16 Jul. 1961 [22], JTP, Sarkant DT, DGA, Sarkand RVR, 2023, [19], *ibid*., 3 Jun. 1941 [22]; ZMP, RVR Merke, 26 Jul. 1952 [22], Karatau, Akbulak usheli, 13 Jul. 1961 [22]; KR: IKR, Jety—Oguz DT, Barskoun GRG, 1931 [79], Djety-ogyz GRG, 1937, [79], small Djergalchal 1948, [79], Mountain of Terskej Alatoo, 26 May 1966, TAAM065175.—on *Pinus pallasiana* D. Don (Pinaceae): UZ: Tashkent city, Tashkent Botanical Garden, Oct. 1984 [15].—on dried stem of angiosperm: UZ: NMP, Mingbuloq DT, Qorasuv garden, May 2003 [15].—on *Pinus sylvestris* L. (Pinaceae): KZ: KTP, Semiozyorniy rayon, Kazanbasskaya FY, 11 Oct. 1944 [22], *ibid*., 16 Jun. 1956 [22], Borovskoye Lake in the Mendykarinsky DT, 20 Aug. 1946 [22], AQL, Sandiktavskiy FY, 6 Jul. 1953 [22], *ibid*., 12 Jul. 1954 [22], *ibid*., 7 Aug. 1954 [22], Aleeksevsky bor, 28 Jun. 1953 [22], Otradenskiy FY, 10 Jul. 1942 [22], *ibid*., 4 Jul. 1952 [22]; NKP, Bulandskiy FY, 3 Jul. 1942 [22], Burabay DT, resort Burabay, 10 May. 1945 [22], Burabay FY, 30 Jul. 1953 [22]; KRP, Karakalinska, 24 Jul. 1954 [22].—on *Larix sibirica* Ledeb. (Pinaceae): KZ: EKP, Katon-Karasayskiy FY, Urilya VLG, 24 Aug. 1961 [22].

***GENUS***: ***SANGHUANGPORUS***

***S. lonicerinus*** (Bondartsev) Sheng H. Wu, L.W. Zhou and Y.C. Dai [≡ *Phellinus lonicerinus* (Bondartsev) Bondartsev and Singer]—on *Acer* sp. (Sapindaceae): UZ: TSP, Bustonliq DT, Onaulgansoy, Pskem RVR, PMR, WTSM, 20 Jun. 2014 [15].—on *Acer tataricum* subsp. *semenovii* (Sapindaceae): UZ: TSP, Angren, Yangibod VLG, southeastern slope of CMR, WTSM, 5 May 2014 [15].—on *Lonicera* sp. (Caprifoliaceae): KZ: ALP, RVR Big Almaty, TLA, 20 May 1941 [22], *ibid*., 7 May 1942 [22], Charyn ash FY, RVR Charyn, 15 Oct. 1941 [22], Turgen VLY, Almaty, 2 May 1984, TAAM105677; JTP, Aksu DT, DGA Mountain range, Tirekli RVR, 2023 [19], Sarkand RVR, 2023 [19], Aksu DT, Lepsinskaya apple forest, 10 Apr. 1944 [22], Panfilov DT, city Panfilov, RVR Usik, 7 Apr. 1943 [22], RVR Terekty, apple forest, 9 Jun. 1944 [22], RVR Chelek, Chelek VLG, 5 Sep. 1941 [22], *ibid*., 8 Sep. 1941 [22]; KR: TSM, [24]; TJ: Varzob DT, HMR, Kondora VLY, Kvak, PAMS, 26 May 1978 TAAM097260, *ibit*., 27 May 1978 TAAM097282, TAAM097290, TAAM097284, TAAM097291, TAAM097218, TAAM097301, *ibid*., 5 Apr. 1977, TAAM096732, TAAM096750, TAAM096751, *ibit*., 6 Apr. 1977, TAAM096762, *ibid*., 7 Apr. 1977, TAAM096769, TAAM096789, Vahdat DT, Romit State Nature Reserve, 11 Apr. 1977, TAAM096830, TAAM096829, *ibit*., 12 Apr. 1977, TAAM096853; KLP, Norak, 25 Apr. 1980, TAAM102814, GBP: Sangvor DT, Tavildara, Shunan road, 22 Jun.1982, TAAM064949, Vanj, Gijovast, RVR Vanj, 8 Jun. 1978, TAAM097352; TR: AHP, Ahal DT, Arvaz, 17 Oct. 1971, TAAM055407, TAAM055395, TAAM055428, *ibit*., 18 Oct. 1971, TAAM055490, Chozly-Dere in Kara-Kala area, 21 Oct. 1971, TAAM055017, KDM, by stream Arvaz, 17 Oct. 1971, TAAM055428a, TAAM203661; BKP, Balkan DT, KDM, Aydere, 16 Apr. 1969, TAAM033119; UZ: TSP, Bustonliq DT, Kuksu RVR, PMR, WTSM, 8 Nov. 2016 [15], *ibid*., 25 Apr. 1982, TAAM104407 [15], *ibid*., 25 Apr. 1982, TAAM104439 [15], UCNNP, WTSM, 8 Nov. 2016 [15], Yubileyniy VLG, Chimyonsoy, Chimgan, 22 Apr. 1982, [15], Chimgan, by the RVR Bolshoy Kok-Saj, 25 Apr. 1982, TAAM104396, *ibit*., 25 Apr. 1982, TAAM203689, Yangikurgan VLG, Kurigansay RVR, WTSM, 24 Apr. 1982 [15], *ibid*., 24 Apr. 1982, TAAM127410 [15], Xojikent VLG, UMR, WTSM, 9 Oct. 2016 [15], Oqtosh VLG, UMR, WTSM, 9 Sep. 2016 [15], Onaulgansoy, Pskem RVR, PMR, WTSM, 19 Jun. 2014 [15], *ibit*., 20 Jun. 2014, [15], Yangikurgan, Kurgan-son stream, 24 Apr. 1982, TAAM127412; JZP, NSR, NRG, PAMS, 11 Sep. 2016, [15], Zaamin DT, ZNP, ZFSP in the south and southeast of the TMR, 26 May 2018 [15], *ibid*., 26 May 2018 [15].—on *Lonicera altmannii* Regel & Schmalh. (Caprifoliaceae): UZ: TSP. Bostonliq DT, Yubileiniy, 25 Apr. 1982, TAAM104277.—on *Lonicera caerulea* subsp. *altaica* (Pall.) Gladkova (Caprifoliaceae): KR: ALP, Charyn ash FY, RVR Charyn, 10 Oct. 1941 [22].—on *Lonicera nummulariifolia* Jaub. & Spach (Caprifoliaceae): KZ: TKP, Talas Alatau, Aksu-Jabagli reserve, 15 Aug. 1961 [22], Urilya VLG, 28 Aug. 1961 [22]; TJ: Varzob DT, Pamir-Alay mountains, HMR, Kondora, 28 May 1978 TAAM102824; TR: Kara-Kala DT Co., KDM, 21 Oct. 1971, TAAM055512, Nokhur, Kara-Suv, 20 Oct. 1971, TAAM055469, *ibit*., 22 Oct. 1971, TAAM055523, TAAM055002, Chozly-Dere in Kara-Kala area, 22 Oct. 1971, TAAM055022, TAAM055023, TAAM054985.—on *Lonicera xylosteum* L. (Caprifoliaceae): KZ: KTP, Central Kazakhstan, RVR Ashu-Tasty, 26 Aug. 1961 [22].—on *Lonicera webbiana* Wall. ex DC. (Caprifoliaceae): KZ: TKR, UMR, WTSM, 31 Jul. 1949 [22]; UZ: TSP, Bustonliq DT, Beldersay, Greater Chimgan, CMR, WTSM, 15 May 2011 [15], Yangikurgan VLG, Kurigansay RVR, WTSM, 9 Oct. 2016 [15].—on deciduous trunk angiosperm: UZ: TP, Parkent DT, Kumyshkan VLG, CMR, WTSM, 29 Apr. 1988 [15], Chatkal Range of Tian Shan, Bashkyzylsay area, 29 Apr. 1988, TAAM126250.

***Note***: New record for Tajikistan and Turkmenistan.

***GENUS***: ***TROPICOPORUS***

***T. linteus*** (Berk. and M.A. Curtis) L.W. Zhou and Y.C. Dai [≡ *Phellinus linteus* (Berk. and M.A. Curtis) Teng]—on *Acer* sp. (Sapindaceae): UZ: JZP, Zaamin DT, ZNP, ZFSP in the south and southeast of the TMR, 4 Sep. 1989 [15].—on *Lonicera* sp. (Caprifoliaceae): TR: AHP, Ahal DT, Kök-Tepe region, KDM, Dushak, 28 Oct. 1971, TAAM055647, TAAM166179, TAAM166180, TAAM166181, TAAM055696, TAAM166179, TAAM166180, TAAM166181; UZ: TSP, Parkent DT, CBR, CMR, WTSM, 9 Apr. 1985 [15].—on *Populus* sp. (Salicaceae): UZ: JZP, NSR, NRG, PAMS, 05 May 1987 [15].—on *Quercus* sp. (Fagaceae): UZ: SMP, ZSR, Zarafshan VLY, PMts., 12 Sep. 1986 [15].—on *Rosa fedtschenkoana* Regel (Rosaceae): UZ: TSP, Bustonliq DT, Yusufhona VLG, Mazarsay, Charvak Reservoir, WTSM, 21 Apr. 1982 [15].—on *Salix* sp. (Salicaceae): UZ: TSP, UCNNP, WTSM, 16 Aug. 1985 [15].—on *Salix wilhelmsiana* (Salicaceae): UZ: ADP, Garden and Parks, Apr. 2000 [15], *ibid*., May 2003 [15].—on *Ulmus* sp. (Ulmaceae): UZ: QDP, HSNR in northwestern of HMR, PAMS, 27 Aug. 1987 [15].—on trunk and stem of angiosperm woody plants: TSP, UCNNP, WTSM, Apr. 1989 [15]. KR: JAP, Aksyeskiy DT, SCNR [81].

***Note***: New record for Turkmenistan.

### 3.2. Diversity and Taxonomic Composition

**Figure 3:**
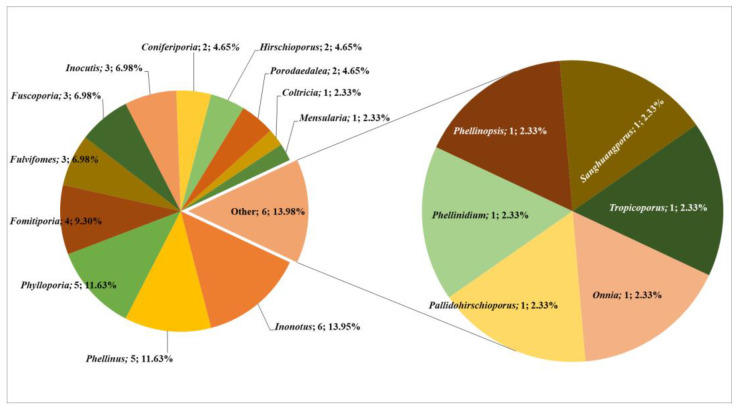
Taxonomic composition of poroid Hymenochaetoid fungi in Central Asia.

Through an exhaustive literature review, re-examination of herbarium specimens, and extensive field data collection, we identified a total of 43 distinct poroid Hymenochaetoid species, belonging to 18 genera. Among the documented genera, *Inonotus* is most species-rich, with six species accounting for 13,95% of the total Hymenochaetoid taxa identified in Central Asia (Figure 3).

This is followed by *Phellinus* and *Phylloporia*, each contributing five species (11.63%), and *Fomitiporia*, with four species (9.30%). The genera *Fulvifomes*, *Fuscoporia*, and *Inocutis* each encompass three species (7%), collectively representing a substantial 61.47% of the total mycobiota. Additional genera such as *Coniferiporia*, *Hirschioporus*, and *Porodaedalea* (each contributing two species, 4.65%) and *Coltricia*, *Mensularia*, *Onnia*, *Pallidohirschioporus*, *Phellinidium*, *Phellinopsis*, *Sanghuangporus*, and *Tropicoporus* (each contributing one species, 2.33%) together comprise the remaining 38.53% of the Hymenochaetoid taxa in Central Asia (Figure 3). Our research has made significant contributions to the understanding of the geographical distribution of these fungi. For instance, *Hirschioporus abietinus* was recorded for the first time in Kyrgyzstan. In Tajikistan, ten species were newly documented: *Coniferiporia weirii, Fomitiporia hippophaeicola, F. robusta, Fulvifomes robiniae, Fuscoporia ferruginosa, Phellinus igniarius, Ph. pomaceus, Phylloporia ephedrae, P. ribis*, and *Sanghuangporus lonicerinus*. Similarly, in Turkmenistan, eleven species were recorded for the first time: *Fulvifomes robiniae, Inocutis dryophila, I. tamaricis, Inonotus hispidus, Phellinidium ferrugineofuscum, Phellinopsis conchata, Phellinus pomaceus, Phylloporia ephedrae, Ph. ribis, Sanghuangporus lonicerinus*, and *Tropicoporus linteus*.

These findings underscore the fact that the inventory of wood-inhabiting mycota in the study area is far from complete. The continuous exploration and documentation of these macrofungi are crucial to fully understanding their diversity and ecological roles. Our study not only expands the taxonomic and geographical knowledge of poroid Hymenochaetoid fungi but also provides a critical baseline for future mycological research and conservation efforts in Central Asia.

The implications of our findings are multifaceted. Firstly, the identification of these species adds to the global database of fungal biodiversity, offering insights into the unique mycobiota of Central Asia. Secondly, the medicinal properties of these fungi, some of which have been used in traditional medicine, highlight their potential for pharmaceutical and therapeutic applications. Further research into their bioactive compounds could lead to the discovery of novel drugs and treatments. Lastly, the documentation of these fungi contributes to the preservation of local mycological knowledge, ensuring that this invaluable information is not lost to future generations.

This study provides a significant contribution to the understanding of fungal diversity and distribution in Central Asia. The discovery of numerous species previously unrecorded in the region emphasizes the prominence of continued mycological research and conservation. By building upon this foundational work, future studies can further elucidate the ecological roles and medicinal potential of these fascinating organisms, ultimately contributing to the broader field of mycology and biodiversity conservation.

### 3.3. Distribution of Poroid Hymenochaetoid Fungi in Central Asia

Poroid Hymenochaetoid macrofungi in Central Asia consist of 43 documented species, as shown in Table 1.

Kazakhstan has the highest diversity, with 30 species across 15 genera, representing a significant proportion of the total. Uzbekistan follows closely with 29 species across 16 genera, emphasizing its rich diversity. The high species count in Uzbekistan points to its potential as a significant area for future mycological studies and bioprospecting. Kyrgyzstan accounts for 19 species across 11 genera, reflecting moderate diversity compared to Kazakhstan and Uzbekistan, indicating a stable yet less explored environment for these fungi. Turkmenistan has eleven species across nine genera, suggesting a smaller but notable presence, likely influenced by specific ecological or climatic conditions. Tajikistan has the fewest species, with only ten across seven genera, highlighting a need for more focused conservation efforts and further research to uncover potentially undiscovered species.

Table 1 provides a detailed overview of species count and distribution across the five Central Asian countries. Kazakhstan and Uzbekistan together represent the majority of macrofungi, with 69.76% and 67.44% of the total species, respectively. Kyrgyzstan’s diversity accounts for 44.18%, showing its importance as a region for these fungi. Turkmenistan and Tajikistan, with 25.58% and 23.25% of the species, respectively, exhibit lower diversity, likely due to less favorable ecological conditions.

Fungal biodiversity is particularly rich in Kazakhstan and Uzbekistan, where species are predominantly found in deciduous and mixed forests within foothill and mountainous regions. These areas serve as vital ecological hotspots, highlighting their importance to conservation efforts. In contrast, fungal diversity in Kyrgyzstan, Turkmenistan, and Tajikistan is comparatively lower, reflecting variations in environmental conditions that influence the distribution and growth of fungal species across the region. Hymenochaetoid species are rare in the urban and mountain forests of Tajikistan and Turkmenistan, where only about 10 species are found. Hymenochaetoid taxa are most commonly found in foothills and mountainous areas, providing ideal habitats for their growth. In Central Asia, these fungi are predominantly distributed in the Central Tian Shan Mountains, including the Fergana range in Kyrgyzstan. The Northern Tian Shan Mountains, encompassing the Trans-Ili Alatau and Dzungarian Alatau ranges in Kazakhstan, also harbor a significant number of species. The Western Tian Shan Mountains, including the Chatkal, Ugam, Kurama, and Pskem ranges in Uzbekistan, are notable for Hymenochaetoid diversity. The Pamir-Alay Mountains, particularly the Hissar range in Tajikistan and the southeastern Turkestan and northwestern Hissar ranges in Uzbekistan, also serve as important habitats for these fungi. These areas constitute more than 70% of the total Hymenochaetoid studies in this region, underscoring their ecological significance. The combination of climatic and environmental conditions in these mountainous regions makes them hotspots for Hymenochaetoid fungi biodiversity.

**Figure 4:**
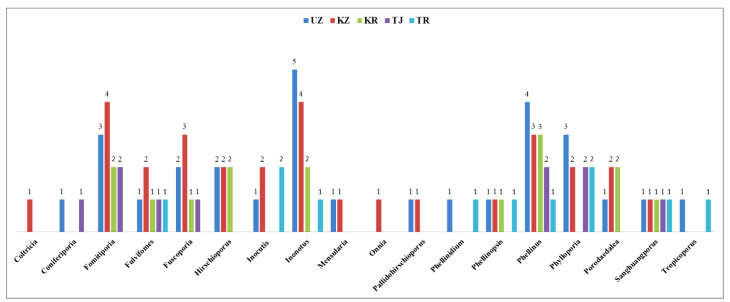
Distribution of poroid Hymenochaetoid genera in five Central Asian countries: Uzbekistan (UZ), Kazakhstan (KZ), Kyrgyzstan (KR), Tajikistan (TJ), and Turkmenistan (TR).

Figure 4 illustrates the distribution of poroid Hymenochaetoid fungal genera across the five Central Asian countries: Uzbekistan, Kazakhstan, Kyrgyzstan, Tajikistan, and Turkmenistan. Uzbekistan hosts sixteen genera (26.2%), while Kazakhstan has fifteen genera (24.6%). Kyrgyzstan has ten genera (16.4%), Turkmenistan has nine (14.8%), and Tajikistan has seven (11.5%).

The most diverse genera include *Fomitiporia, Fulvifomes, Fuscoporia, Hirschioporus*, and *Phellinus*, reflecting varied ecological conditions ranging from steppes and deserts to mountainous regions. The diversity observed in Uzbekistan and Kazakhstan reflects their varied ecological conditions and favorable environments for macrofungi. Kyrgyzstan also shows moderate diversity, influenced by its diverse topography. In contrast, Tajikistan and Turkmenistan have lower hymenochaetoid diversity, likely due to more challenging climatic conditions and less diverse habitats. These distribution patterns indicate that regional climatic factors, habitat diversity, and geographical features play crucial roles in shaping macrofungal diversity across Central Asia.

In Uzbekistan, notable genera include *Inonotus* (five species, 10.9%), *Phellinus* (four species, 8.7%), and *Phylloporia* (three species, 6.5%). Other genera, such as *Fomitiporia* (three species, 6.5%), *Fulvifomes* (one species, 2.2%), and others like *Coniferiporia*, *Phellinidium*, *Phellinopsis*, and *Sanghuangporus* (each with one species, 2.2%), also contribute to the diversity. The diversity in Uzbekistan reflects its varied habitats, from deserts to mountainous areas. In Kazakhstan, notable genera include *Fomitiporia* (four species, 8.9%), *Fuscoporia* (three species, 6.7%), and *Phellinus* (three species, 6.7%). Other genera, such as *Hirschioporus* and *Fulvifomes* (two species each, 4.4%), and several with one species, such as *Coltricia*, *Coniferiporia*, *Mensularia*, *Onnia*, and *Pallidohirschioporus* (2.2% each), contribute to the diversity, reflecting the varied ecological conditions, from steppes to mountains. Kyrgyzstan records ten genera, including *Phellinus* with three species (8.1%) and *Porodaedalea*, *Hirschioporus*, *Fomitiporia*, and *Phylloporia* with two species each (5.4%). Other genera, such as *Sanghuangporus*, are represented by one species (2.7%). In Turkmenistan, nine genera are recorded, with *Inocutis* and *Phylloporia* each having two species (6.5%). Other genera, such as *Fulvifomes*, *Inonotus*, *Phellinus*, *Phellinidium*, *Phellinopsis*, *Tropicoporus*, and *Sanghuangporus*, are represented by one species each (4.5%). The limited diversity in Turkmenistan is likely due to its predominantly arid climate and fewer forested areas. Tajikistan records seven genera, with key genera such as *Fomitiporia*, *Phellinus*, and *Phylloporia* each having two species (6.5%). Other genera represented by one species each include *Coniferiporia*, *Fulvifomes*, *Fuscoporia*, *Mensularia*, and *Sanghuangporus* (3.2% each). The lower diversity in Tajikistan may be attributed to its rugged terrain and less explored macrofungal habitats. In summary, the distribution of Hymenochaetoid fungi across Central Asia indicates that Kazakhstan and Uzbekistan are the most diverse regions, offering ample opportunities for research, conservation, and sustainable utilization of these fungi. The notable diversity of genera and species, particularly in Uzbekistan and Kazakhstan, underscores their ecological significance and potential for discovering new species.

### 3.4. Host Preference

The study area contains about 600 arborescent plant species, including 100–150 tree species and various shrubs [3]. Notable species include *Abies sibirica*, *Picea schrenkiana*, and desert shrubs such as *Haloxylon persicum* and *H. aphyllum*. High species diversity and endemism are evident in genera such as *Betula*, *Calligonum*, *Cotoneaster*, *Crataegus*, *Malus, Prunus*, *Pyrus*, *Rosa*, and *Tamarix*. The mountains of Central Asia, recognized as a global biodiversity hotspot [1], support over 300 wild fruit and nut species, including wild apple (*Malus* spp.); almond, apricot, cherry, and plum (*Prunus* spp.); walnut (*Juglans regia*); pear (*Pyrus* spp.); pistachio (*Pistacia vera*); pomegranate (*Punica granatum*); and seaberry (*Hippophae rhamnoides*). Most broadleaved fruit and nut forests are found in the foothills and slopes of the Tien Shan, Pamir-Alay, and Kopetdag Mountains between 800 and 2000 m. Key species include *Juniperus seravschanica*, *J. semiglobosa*, and *J. turkestanica*. The woody flora of the region is a mix of Siberian, Mediterranean, Indo-Himalayan, and Iranian elements, with host diversity being crucial for fungal groups in the study area.

Species of Hymenochaetoid fungi were found on 125 host plant species of 42 genera and 25 families in the countries of Central Asia (Table 2).

The host plant families with the greatest number of hymenochetoid species are Rosaceae (8 genera and 39 host plant species) and Salicaceae (2; 21) with 26 fungal species in each plant family; Pinaceae (4; 9) with 21; Betulaceae (2; 6) with 13; Oleaceae (3; 4), Fagaceae (2; 2), Ulmaceae (1; 5), and Juglandaceae (1;1) with 7 species in each family; and Sapindaceae (1; 3) with 5 species, representing 80.0% of all Hymenochaetoid fungi species present in the study area out of 24 genera and 90 (72%) out of 125 host species. Collectively, nine families of host plants account for approximately 80% of the fungal species present in the study area; the other families of plant hosts (17; 35) present one to three macrofungal species (Table 3).

**Figure 5:**
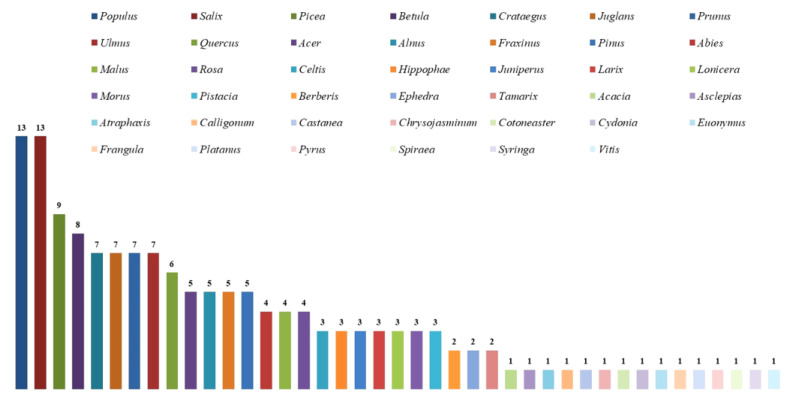
Occurrence numbers of poroid Hymenochaetoid fungi on most representative host genera.

The highest number of poroid Hymenochaetoid basidiomycete species is reported in the following host genera: *Populus* and *Salix* (26 species, 61.90% of the total fungal species number); *Picea* (9, 21.42.6%); *Betula* (8, 19.04%); *Crataegus*, *Juglans*, *Prunus*, and *Ulmus* (each 7, 16.66%); *Quercus* (6, 14.28%); *Acer*, *Alnus*, *Fraxinus*, and *Pinus* (each 5, 11.90%); *Abies*, *Malus*, and *Rosa* (each 4, 9.52%); *Celtis, Hippophae, Juniperus*, *Larix*, *Lonicera*, *Morus*, and *Pistacia* (each 3, 7.14%); and other plant genera (*Acacia*, *Asclepias*, *Atraphaxis*, *Berberis*, *Calligonum*, *Castanea*, *Chrysojasminum*, *Cotoneaster*, *Cydonia*, *Ephedra*, *Euonymus*, *Frangula*, *Tamarix*, *Platanus*, *Pyrus*, *Spiraea*, *Syringa*, and *Vitis*) host one to two fungal species (Figure 5).

Numerous Hymenochaetoid poroid were found on *Picea schrenkiana* (9 species of 7 genera); *Juglans regia* (7; 5); *Salix wilhelmsiana* (5; 4); *Betula pendula* and *Populus tremula* (each 4; 4); *Abies sibirica*, *Betula tianschanica*, and *Malus domestica* (each 4; 3); and other trees. In particular, macrofungal species as *Coniferiporia uzbekistanensis*, *Fomitiporia robusta*, *Fuscoporia torulosa*, *Hirschioporus abietinus*, *Inonotus hispidus*, *Phellinus pomaceus*, *Ph. igniarius*, *Phellinopsis conchata*, *Porodaedalea pini*, *Phylloporia ephedrae*, and *Sanghuangporus lonicerinus* are widespread and harmful to members of plant species belonging to Betulaceae, Caprifoliaceae, Cupressaceae, Ephedraceae, Juglandaceae, Oleaceae, Pinaceae, Rosaceae, Salicaceae, and Tamaricaceae families (Table 3, Figure 5).

Among the poroid Hymenochaetoid fungi, 20 genera and some species were associated with a wide range of plant hosts: *Phellinus* was found on 15 host genera (*Acer, Alnus*, *Berberis*, *Betula*, *Celtis*, *Crataegus*, *Cydonia*, *Juglans*, *Lonicera*, *Malus*, *Picea*, *Populus*, *Prunus*, *Salix*, and *Ulmus*) and 40 plant species, followed by *Inonotus* (27 species belonging to 16 host genera), *Phylloporia* (22; 12), *Fuscoporia* (22; 16), *Fomitiporia* (21; 15), *Phellinopsis* (14; 8), *Inocutis* (9; 4), *Sanghuangporus* and *Tropicoporus* (each 9; 2), *Hirschioporus* (6; 3), *Fulvifomes* (5; 5), *Porodaedalea* (5; 4), *Mensularia* (4; 4), *Pallidohirschioporus* and *Phellinidium* (each 3; 3), and other genera are found on 4 host genera and 4 plant species (Table 4).

Regarding host preferences, some Hymenochaetoid species exhibit a broad ecological range within the study area, as shown in Table 4. For example, *Phellinus igniarius* was recorded on twenty-one plant species of nine genera (*Acer* sp., *Alnus glutinosa*, *Betula pendula*, *B. pubescens*, *B. tianschanica*, *Juglans regia*, *Picea schrenkiana*, *Populus alba*, *Prunus* sp., *P. padus*, *P. vulgari, Salix* sp., *S. acutifolia*, *S. alba*, *S. caprea*, *S. songarica*, *S. starkeana*, *S. tenuijulis*, *S. turanica*, *S. wilhelmsiana*, and *Ulmus laevis*), followed by *Phellinus pomaceus* on twenty species of nine genera (*Berberis turcomanica*, *Celtis australis* subsp. *caucasica*, *Crataegus chlorocarpa*, *Cydonia oblonga*, *Juglans regia*, *Lonicera* sp., *Malus* sp., *M. domestica*, *Prunus* sp., *P. cerasifera*, *P. domestica*, *P. mahaleb*, *P. erythrocarpa*, *P. dulcis*, *P. griffithii* var. *tianshanica*, *P. persica*, *P. spinosa*, *P. microcarpa*, *P. turcomanica*, *Salix* sp.), *Inonotus hispidus* on nineteen species of fourteen genera (*Acer negundo*, *Asclepias syriaca*, *Celtis australis subsp. caucasica*, *Juglans regia*, *Fraxinus sogdiana*, *Malus* sp., *M. sieversii*, *M. domestica*, *Morus alba*, *Platanus orientalis*, *Pinus* sp., *Prunus avium*, *Populus alba*, *P. macrocarpa*, *Ulmus* sp., *U. minor* subsp. *minor*, *U. pumila*, *Salix* sp., *Tamarix* sp.), *Phellinopsis conchata* on fourteen species of eight genera (*Alnus* sp., *Juniperus polycarpos* var. *turcomanica*, *Picea schrenkiana*, *Populus* sp., *P. tremula*, *Rosa* sp., *Salix bebbiana*, *S. capusii*, *S. lanata* subsp. *lanata*, *S. tenuijulis*, *S. triandra*, *Syringa* sp., *S. vulgaris*, *Ulmus* sp.), *Fomitiporia robusta* recorded on twelve species of ten genera (*Atraphaxis pyrifolia*, *Castanea* sp., *Hippophae rhamnoides*, *Juglans regia*, *Morus alba*, *Picea schrenkiana*, *Pistacia* sp., *Quercus* sp., *Salix wilhelmsiana*, *Spiraea* sp., *S. crenata*, *S. hypericifolia*), and *Fuscoporia torulosa* and *Phylloporia ephedrae* are each on eleven species of nine/five genera (*Celtis australis* subsp. *caucasica*, *Crataegus* sp., *C. chlorocarpa*, *C. pseudoheterophylla* subsp. *turkestanica*, *C.* × *zangezura nothosubsp*. *pseudoambigua*, *Chrysojasminum fruticans*, *Ephedra* sp., *E. equisetina*, *E. intermedia*, *Fraxinus sogdiana*, *Malus domestica*, *M. sieversii*, *Prunus* sp., *Prunus bucharica*, *Pyrus communis*, *Quercus* sp., *Rosa* sp., *Rosa canina*, *R*.× *karakalensis*, *Salix babylonica*, *S. wilhelmsiana*). In addition, among Hymenochaetoid taxa, species such as *Sanghuangporus lonicerinus*, *Fomitiporia punctata*, *Tropicoporus linteus*, *Hirschioporus abietinus*, *Phylloporia ribis*, *Ph. yuchengii*, *Inonotus obliquus*, and *I. pseudohispidus* exhibit a moderate host range, being reported on fifty-seven host species. Twenty-seven other Hymenochaetoid genera were found on sixty-three host species, with a total of fifty-two genera, and each species colonized one to ten plant species (Table 4).

### 3.5. Ecological Roles: Decomposition and Pathogenic Impacts

Wood-inhabiting Hymenochaetoid fungi play vital ecological roles in forest ecosystems, primarily through their contributions to decomposition and pathogen interactions. These fungi play a key role in nutrient cycling and habitat formation and potentially influence forest health. Depending on the type of decay they cause, wood decay fungi are broadly categorized into white-rot fungi, brown-rot fungi, and soft-rot fungi [83]. The majority of wood-decaying fungal species belong to the Basidiomycetes, which are predominantly classified as either white-rot or brown-rot fungi [84]. Other fungal groups, although less common, also include species capable of degrading cellulose and hemicellulose. Poroid Hymenochaetoid fungal species exhibit a multi-hosted nature, colonizing trees and shrubs from diverse plant families in the study area. The distribution pattern of these wood-inhabiting basidiomycete macrofungi correlates with the high diversity of hardwood species in Central Asia [14,15,16,17,18,19,20]. These Hymenochaetoid species are associated with various diseases and decay processes affecting woody plants in the region [15,17,18,19,20,85,86,87,88,89]. To date, more than 150 wood-inhabiting basidiomycete macrofungi have been documented on more than 100 woody plant species in Uzbekistan alone [14,15,16,20,26,27,33].

#### 3.5.1. Decomposition and Nutrient Cycling

Hymenochaetoid fungi play a crucial role as decomposers in forest ecosystems, specializing in the breakdown of lignin and cellulose, which are the major structural components of wood. By decomposing deadwood, these fungi facilitate nutrient cycling, releasing essential nutrients such as carbon, nitrogen, and phosphorus back into the soil, where they are utilized by plants and other organisms. This process enhances soil fertility and supports the growth of understory vegetation, thus contributing to ecosystem health. Most poroid Hymenochaetoid fungi species reported in Central Asia are saprophytic wood decomposers, possessing powerful enzymes that can degrade lignocellulose effectively [15,33,84]. These species not only play a vital role in forest ecosystems but also have potential biotechnological applications. Several poroid taxa have been identified as having potential uses in biotechnology, as suggested by Badalyan and Gharibyan [50] and Gafforov et al. [16].

In Central Asia, most poroid Hymenochaetoid fungi are efficient decomposers of lignin and cellulose. Species such as *Fomitiporia robusta* and *F. punctata*, which have been recorded on a range of host trees, including *Betula* (birch), *Castanea* (chestnut), and *Juglans regia* (walnut), are particularly active in decomposing deadwood. These fungi break down complex organic materials into simpler forms, facilitating the recycling of nutrients throughout the ecosystem. This function is especially critical in mixed and deciduous forests found in montane regions such as the Tien Shan and Pamir-Alai Mountains, where the availability of diverse host trees like *Juglans*, *Betula*, and *Crataegus* fosters rich habitats for wood-decaying fungi.

In addition to nutrient cycling, the decomposition activities of fungi such as *Inonotus hispidus*, which is widespread across Uzbekistan, Kazakhstan, and Kyrgyzstan, help prevent the excessive accumulation of organic material, which can foster pests and diseases. Decomposing fungi also contribute to the formation of microhabitats within forests [83]. Decaying logs and hollow trees provide shelter and habitats for a variety of species, including insects, small mammals, and other fungi [83]. For instance, *Hirschioporus abietinus*, recorded on coniferous hosts such as *Pinus sylvestris* and *Abies sibirica*, is particularly significant in the mountainous regions of Kazakhstan and Uzbekistan, where it aids in creating microhabitats by hollowing out tree trunks.

#### 3.5.2. Pathogenic Impacts on Trees

Although resource recycling functions are generally beneficial to trees, forests, and humans, some wood-inhabiting fungi are forest pathogens that inhabit living trees, according to previous studies [14,15,16,17,18,19,20,21,22,23,24,25,26,27,28,85,86,87,88,89,90,91,92,93,94]. In this study, some of the recorded poroid Hymenochaetoid fungi are identified as forest pathogens [15,17,18,19,20,33]. These species deserve attention to prevent ecological and economic losses arising from their pathogenicity. For example, several poroid Hymenochaetoid fungi, such as the causal agents of some disease on apple trees, lead to reduced yields. Species of *Coniferiporia* (*C. uzbekistanensis, C. weirii*) and *Porodaedalea* (*P. chrysoloma, P. pini*) are significant pathogens infecting living trees in the genera *Juniperus*, *Picea*, and *Pinus*. Species of *Inonotus*, *Phylloporia*, and *Phellinus*, along with some species from related genera, can cause root rot disease in their hosts. Additionally, some species produce stem cankers on living deciduous trees in the Tien Shan and Pamir-Alai Mountains in Central Asia, affecting species such as *Acer*, *Crataegus*, *Celtis*, *Fraxinus*, *Juglans*, *Malus*, *Quercus*, and other woody plants. *Inonotus hispidus* is widespread in the walnut-fruit forests of Kyrgyzstan, Tajikistan, and Uzbekistan, damaging up to 4% of the trees of *Juglans regia* and *Malus sieversii* in the Pamir-Alai and Tien Shan Mountains. This species primarily infects living trees, causing canker and heart rot, which significantly impact the health and productivity of walnut and apple trees. *Phellinus igniarius* has been observed as both a pathogen and saprotroph of deciduous trees, including *Acer*, *Alnus*, *Betula*, *Juglans*, *Salix*, and *Ulmus*, in several state parks and reserves across Kazakhstan, Tajikistan, Kyrgyzstan, and Uzbekistan. This species is known to cause white rot in its hosts, often leading to substantial decay in standing trees. Species of *Phylloporia* are less common but also noteworthy pathogens. For instance, *Phylloporia ampelina* is reported on *Vitis vinifera* (grapevine), causing significant wood decay that can lead to economic losses in vineyards. *Phylloporia ephedrae* has been observed on various hosts, including *Crataegus* and *Ephedra* species, contributing to root and stem decay. *Phylloporia yuchengii* has been found on *Crataegus* sp., *Crataegus pseudoheterophylla* subsp. *turkestanica*, *Juglans regia*, *Morus alba*, *Prunus* sp., and *Populus* sp.; it exhibits parasitic behavior on these hosts. Some species of *Fomitiporia**,** Fuscoporia**,** Inonotus**,** Phellinus*, *Fulvifomes*, and *Fuscoporia* have been reported on both living and dead trees, exhibiting both saprotrophic and pathogenic decay on their host wood. The forest diseases caused by these wood-inhabiting poroid fungi and the corresponding economic losses should be considered by relevant management departments. Preventative measures are crucial to protect the affected forests and maintain ecosystem health.

### 3.6. Analyzing the Potential Distribution Patterns of Poroid Hymenochaetoid Fungi in Central Asia

This study employed MaxEnt modeling to predict the potential suitable habitats and analyze the distribution patterns of poroid Hymenochaetoid fungi across Central Asia. The optimal parameters for the model were determined as RM = 1.5 and FC = LQH. The average AUC value for the 10-fold repeated training set was 0.920 ± 0.021, and the average AUC value for the test set was 0.913 ± 0.018, indicating that the model’s predictive performance was outstanding and that the results were reliable (Figure S3).

Key environmental variables influencing the current potential distribution of poroid Hymenochaetoid fungi in Central Asia include host plant density, Bio7 (annual temperature range), Bio10 (mean temperature of the warmest quarter), Bio12 (annual precipitation), Bio19 (precipitation of the coldest quarter), Bio15 (precipitation seasonality), Bio3 (isothermality), Bio2 (mean diurnal temperature range), and Bio17 (precipitation of the driest quarter). Among these variables, host plant density, Bio7, and Bio10 are the most crucial, contributing 75.1%, 6.6%, and 5.5%, respectively, to the MaxEnt model (Table 5). This suggests that Hymenochaetoid fungi in Central Asia have strong relationships with their host plants, and their growth is particularly sensitive to temperature variations.

**Figure 6:**
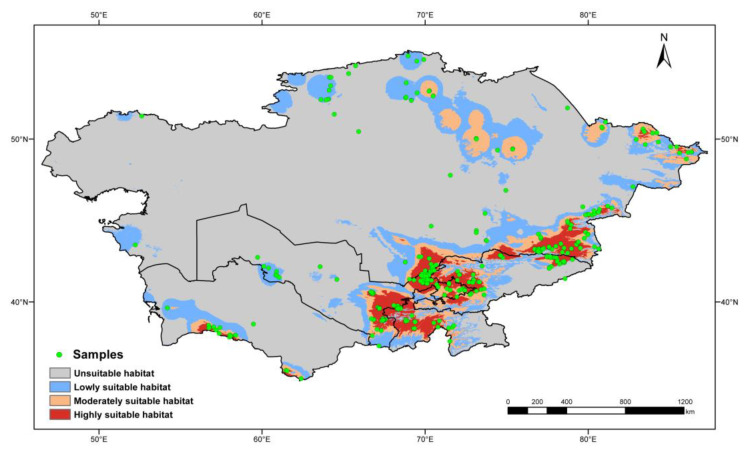
Current potential distribution patterns of Hymenochaetoid fungi in Central Asia predicted by MaxEnt modeling. Green points represent known occurrences of Hymenochaetoid fungi, and colored regions indicate varying habitat suitability levels.

The suitability of habitats for poroid Hymenochaetoid fungi in Central Asia was classified into four categories using Jenks’ natural breaks method: unsuitable (0–0.09), lowly suitable (0.09–0.30), moderately suitable (0.30–0.60), and highly suitable (0.60–1). The unsuitable habitat occupies 350.860 × 10^4^ km^2^ (75.26%), while the total suitable potential area covers approximately 115.08 × 10^4^ km^2^ (24.74%), with lowly, moderately, and highly suitable areas occupying 63.301 × 10^4^ km^2^ (13.55%), 30.643 × 10^4^ km^2^ (6.67%), and 21.134 × 10^4^ km^2^ (4.52%), respectively. Highly suitable habitats are primarily located in the border regions between Kazakhstan and Kyrgyzstan, as well as between Tajikistan and Uzbekistan, characterized mainly by plateaus and mountains (Figure 6).

The model identified several key regions in Central Asia that provide suitable habitats for these macrofungi, with the Tien Shan and Pamir-Alay Mountain ranges emerging as crucial areas due to the diversity of their microclimates, which support a wide variety of fungal species.

### 3.7. Conservation and Future Research

The conservation of Hymenochaetoid fungi in Central Asia is essential due to their ecological significance and potential medicinal value. These fungi play critical roles in nutrient cycling, decomposition, and sustaining forest health. However, their diversity is increasingly threatened by habitat destruction, climate change, and limited research initiatives. To protect these important species, it is crucial to prioritize habitat conservation, conduct regular monitoring, and establish fungal reserves in key biodiversity areas such as the Tien Shan and Pamir-Alay Mountains.

Future research should prioritize comprehensive surveys of underexplored regions, isolation and characterization of novel bioactive compounds, and exploration of their pharmaceutical potential. Collaboration among mycologists, ecologists, and pharmacologists is key to discovering new medicinal compounds and expanding our understanding of the ecological roles of these fungi. Additionally, molecular studies and genomic analyses are needed to elucidate genetic diversity and evolutionary relationships within Hymenochaetoid fungi, providing insights into their adaptation to diverse habitats and environmental changes.

## 4. Conclusions

In this comprehensive mycological and botanical study, we compiled the most detailed annotated checklist of poroid Hymenochaetoid fungi across five Central Asian countries, documenting macrofungal species along with their associated hosts. We analyzed taxonomic diversity, distribution, host preferences, ecological roles (including their functions as forest and saprophytic pathogens), and potential distribution patterns. The data provide crucial insights for the conservation and sustainable use of these fungal resources and their specific hosts. This study improves the understanding of the distribution dynamics of Hymenochetoid fungi in Central Asia. It also provides valuable scientific evidence and decision support for the management of forest diseases in the context of climate change in other regions worldwide.

## Figures and Tables

**Table 1:** Distribution of poroid Hymenochaetoid fungi by countries of Central Asia.

Genera	Total Species	Central Asian Countries				
		KZ	UZ	KR	TJ	TR
*Coltricia*	1	1				
*Coniferiporia*	2		1		1	
*Fomitiporia*	4	4	3	2	2	
*Fulvifomes*	3	2	1	1	1	1
*Fuscoporia*	3	3	2	1	1	
*Hirschioporus*	2	2	2	2		
*Inocutis*	3	2	1			2
*Inonotus*	6	4	5	3		1
*Mensularia*	1	1	1			
*Onnia*	1	1				
*Pallidohirschioporus*	1	1	1			
*Phellinidium*	1		1			1
*Phellinopsis*	1	1	1	1		1
*Phellinus*	5	3	4	3	2	1
*Phylloporia*	5	2	3	2	2	2
*Porodaedalea*	2	2	1	2		
*Sanghuangporus*	1	1	1	1	1	1
*Tropicoporus*	1		1	1		1
Total: 18	43	30	29	19	10	11
Percentage occurring in each country	100	69.76%	67.44%	44.18%	23.25%	25.58%

Abbreviation: Kazakhstan, KZ; Kyrgyzstan, KR; Tajikistan, TJ; Turkmenistan, TR; Uzbekistan, UZ.

**Table 2:** Taxonomic composition of host plants of poroid Hymenochaetoid fungi in Central Asia.

Plant Family	Number Host Genus	Number Host Species	Plant Family	Number Host Genus	Number Host Species
Pinaceae	4	9	Celastraceae	1	1
Fabaceae	1	1	Rhamnaceae	1	1
Sapindaceae	1	3	Juglandaceae	1	1
Betulaceae	2	6	Cupressaceae	1	2
Apocynaceae	1	1	Caprifoliaceae	1	7
Polygonaceae	2	2	Moraceae	1	2
Berberidaceae	1	4	Anacardiaceae	1	2
Fagaceae	2	2	Platanaceae	1	1
Cannabaceae	1	2	Salicaceae	2	21
Oleaceae	3	4	Tamaricaceae	1	4
Rosaceae	8	39	Ulmaceae	1	5
Elaeagnaceae	1	1	Vitaceae	1	1
Ephedraceae	1	3			
Total: 13	28	77	Total: 12	13	48

**Table 3:** Number of host family, genus, and species and number of poroid Hymenochaetoid basidiomycete fungi on host family and genus in Central Asia.

Host Family	Host Genera	Number of Host Species	Number of Fungal Species
Rosaceae	*Cotoneaster, Crataegus, Cydonia, Malus, Prunus, Pyrus, Rosa, Spiraea* (8)	39	26
Salicaceae	*Populus*, *Salix* (2)	21	26
Pinaceae	*Abies, Larix, Picea, Pinus* (4)	9	21
Betulaceae	*Alnus, Betula* (2)	6	13
Fagaceae	*Castanea*, *Quercus* (2)	2	7
Juglandaceae	*Juglans* (1)	1	7
Oleaceae	*Chrysojasminum, Fraxinus, Syringa* (3)	4	7
Ulmaceae	*Ulmus* (1)	5	7
Sapindaceae	*Acer* (1)	3	5
Subtotal	24	90	119
Other families (16)	*Acacia*, *Asclepias*, *Atraphaxis*, *Berberis*, *Calligonum*, *Celtis*, *Ephedra*, *Euonymus*, *Frangula*, *Hippophae*, *Juniperus*, *Lonicera*, *Morus*, *Platanus*, *Pistacia*, *Tamarix*, *Vitis* (18)	35	32
Total 25	41	125	n.a

**Table 4:** Host species numbers of 15 poroid Hymenochaetoid basidiomycete species with the widest host range.

Fungal Genera	Host Genus	Host Species	Fungal Species	Host Genus	Host spp.
*Phellinus*	15	43	*Phellinus igniarius*	9	24
*Inonotus*	16	29	*Phellinus pomaceus*	9	20
*Phylloporia*	12	23	*Inonotus hispidus*	14	19
*Fuscoporia*	16	22	*Phellinopsis conchata*	8	14
*Fomitiporia*	15	21	*Fomitiporia robusta*	10	12
*Phellinopsis*	8	14	*Fuscoporia torulosa*	9	12
*Inocutis*	4	9	*Phylloporia ephedrae*	5	11
*Sanghuangporus*	2	9	*Sanghuangporus lonicerinus*	2	9
*Tropicoporus*	2	9	*Tropicoporus linteus*	7	8
*Hirschioporus*	3	6	*Phylloporia ribis*	6	8
*Fulvifomes*	5	5	*Fomitiporia punctata*	5	8
*Porodaedalea*	4	5	*Hirschioporus abietinus*	5	7
*Mensularia*	4	4	*Inonotus obliquus*	5	6
*Pallidohirschioporus*	3	3	*Phylloporia yuchengii*	5	6
*Phellinidium*	3	3	*Inonotus iliensis*	4	6
Other genus (3)	4	4	Other species (27)	52	63

**Table 5:** Environmental variables and contributions to MaxEnt modeling for the potential distribution of Hymenochaetoid fungi in Central Asia.

Variable	Description	Unit	Contribution (%)
Bio1	Annual mean temperature	°C	–
Bio2	Mean diurnal temperature range	°C	0.2
Bio3	Isothermality (Bio2/Bio7) (×100)	%	0.5
Bio4	Temperature seasonality (standard deviation ×100)	°C	–
Bio5	Max temperature of the warmest month	°C	–
Bio6	Min temperature of the coldest month	°C	–
Bio7	Annual temperature range (Bio5–Bio6)	°C	6.6
Bio8	Mean temperature of the wettest quarter	°C	–
Bio9	Mean temperature of the driest quarter	°C	–
Bio10	Mean temperature of the warmest quarter	°C	5.5
Bio11	Mean temperature of the coldest quarter	°C	–
Bio12	Annual precipitation	mm	4.6
Bio13	Precipitation of the wettest month	mm	–
Bio14	Precipitation of the driest month	mm	–
Bio15	Precipitation seasonality (coefficient of variation)	%	2.9
Bio16	Precipitation of the wettest quarter	mm	–
Bio17	Precipitation of the driest quarter	mm	0.1
Bio18	Precipitation of the warmest quarter	mm	–
Bio19	Precipitation of the coldest quarter	mm	4.6
Altitude	Altitude	m	–
Host plant	Host plant density	tree/km^2^	75.1

Note: variables excluded from modeling due to autocorrelation are indicated by a dash (–).

## Data Availability

The original contributions presented in this study are included in the article/Supplementary Material. Further inquiries can be directed to the corresponding authors.

## Supplementary Materials

The following supporting information can be downloaded at: https://www.mdpi.com/article/10.3390/jof11010037/s1. Figure S1: Density of the host plants of Hymenochaetoid fungi in Central Asia; Figure S2: Correlation of the 21 environmental variables used for predicting the current potential distribution of Hymenochaetoid fungi in Central Asia; Figure S3: The value of the area under receiver operator characteristic curve (AUC) generated by the MaxEnt modeling (10 replications); Table S1: Spatial coordinates and host plant/substrate associations of poroid Hymenochaetoid fungi in Central Asia.
