# Supplementary material for: Annotated Checklist of Poroid Hymenochaetoid Fungi in Central Asia: Taxonomic Diversity, Ecological Roles, and Potential Distribution Patterns
Source: J Fungi (Basel). 2025 Jan 5;11(1):37. doi: 10.3390/jof11010037 (PMC11767013; doi:10.3390/jof11010037)
Supplement: Supplementary file 1 [file jof-11-00037-s001.zip › jof-3345577-supplementary.pdf]

Journal of Fungi

MDPI

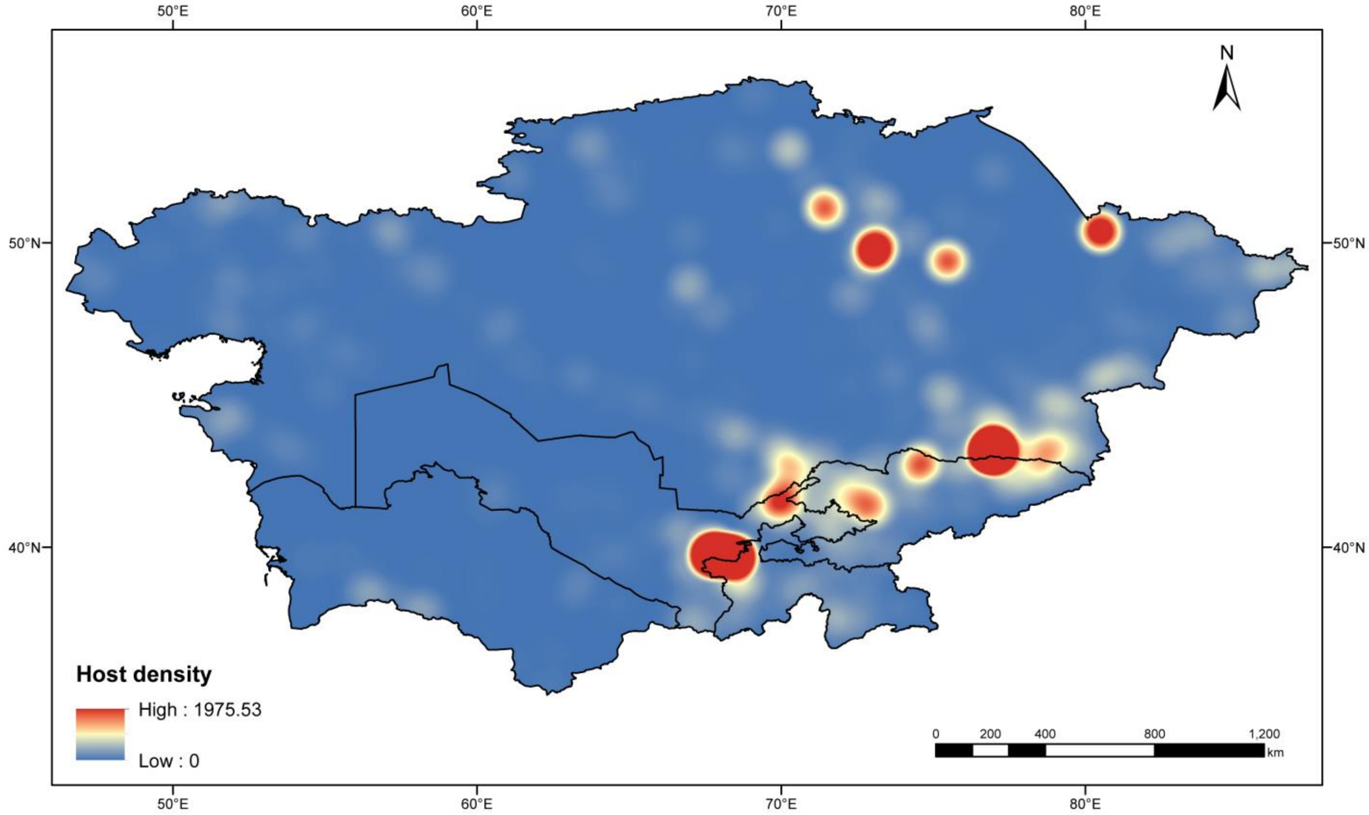

Figure S1. Density of the host plants of Hymenochaetoid fungi in Central Asia.

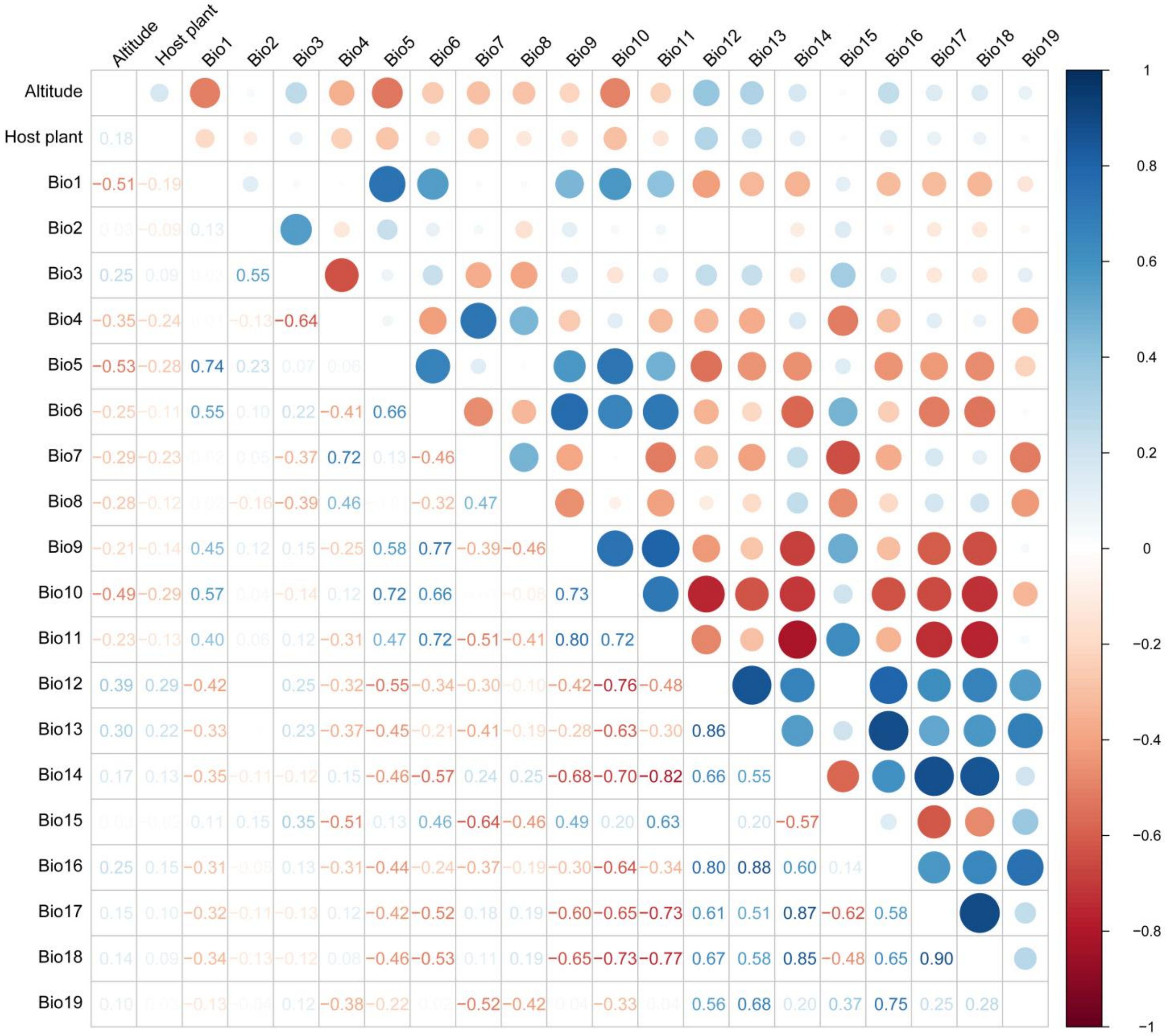

Figure S2. Correlation of the 21 environmental variables used for predicting the current potential distribution of Hymenochaetoid fungi in Central Asia.

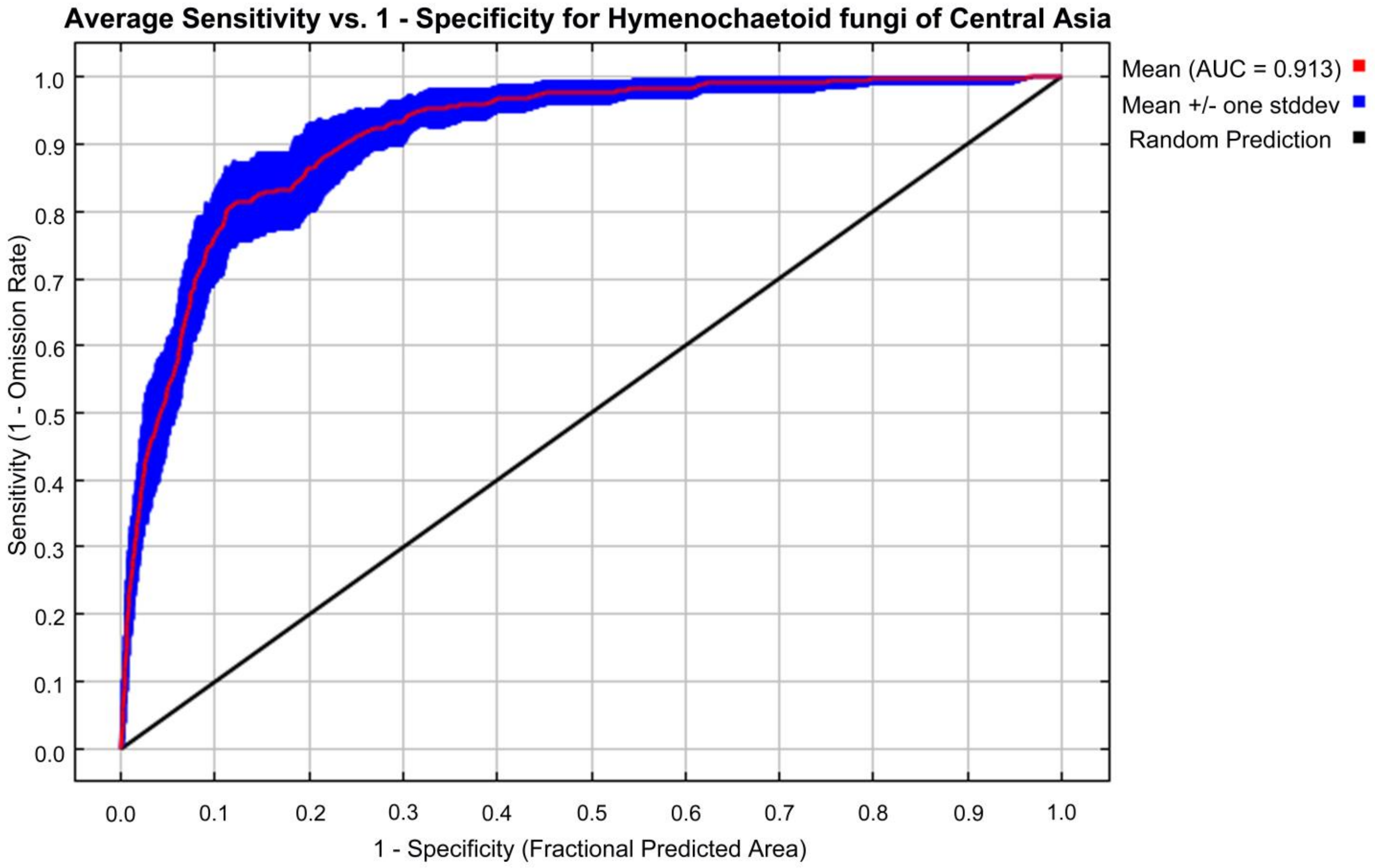

Figure S3. The value of the area under receiver operator characteristic curve (AUC) generated by the MaxEnt modeling (10 replications).

Table S1. Spatial coordinates and host plant/substrate associations of poroid Hymenochaetoid fungi in Central Asia.

Species	Substrate	Latitude	Longitude
Coltricia perennis (L.) Murrill.	Conifers	53.795394444	64.138147222
Coltricia perennis (L.) Murrill.	Conifers	53.790572222	64.129466667
Coltricia perennis (L.) Murrill.	Conifers	53.279008333	64.206913889
Coltricia perennis (L.) Murrill.	Conifers	52.406055556	63.912513889
Coltricia perennis (L.) Murrill.	Conifers	52.451425000	63.921041667
Coltricia perennis (L.) Murrill.	Conifers	52.449372222	64.070527778
Coltricia perennis (L.) Murrill.	Conifers	52.523052778	68.798002778
Coltricia perennis (L.) Murrill.	Conifers	49.405111111	75.370100000
Coltricia perennis (L.) Murrill.	aspen	50.043722222	73.126552778
Coltricia perennis (L.) Murrill.	birch	54.500222222	65.738586111
Coltricia perennis (L.) Murrill.	sagebrush	54.515411111	65.757113889
Coltricia perennis (L.) Murrill.	fescue	49.324111111	74.442527778
Coltricia perennis (L.) Murrill.	Conifers	50.368511111	83.954577778
Coltricia perennis (L.) Murrill.	Conifers	50.366752778	83.965452778
Coniferiporia uzbekistanensis L.W. Zhou,	Juniperus polycarpos var. seravschanica	38.90468	68.81088
Coniferiporia uzbekistanensis L.W. Zhou,	Juniperus polycarpos var. seravschanica	38.90468	68.81088
Coniferiporia weirii (Murrill) L.W. Zhou &	Juniperus polycarpos var. seravschanica	38.90468	68.81088
Fulvifomes kravtzevii (Schwarzman) Y.C.	Calligonum aphyllum (Pall.) Gürke	44.385558333	73.139269444
Fulvifomes kravtzevii (Schwarzman) Y.C.	Calligonum aphyllum (Pall.) Gürke	44.257188889	73.136400000
Fulvifomes kravtzevii (Schwarzman) Y.C.	Calligonum aphyllum (Pall.) Gürke	43.768863889	73.743586111
Fulvifomes rimosus (Berk.) Fiasson and N	Pistacia vera L.	42.771386111	69.656188889
Fulvifomes rimosus (Berk.) Fiasson and N	Pistacia vera L.	41.20454444	72.69364167
Fulvifomes rimosus (Berk.) Fiasson and N	Pistacia vera L.	41.34035000	72.94411111
Fulvifomes rimosus (Berk.) Fiasson and N	Pistacia vera L.	40.43271111	73.60348056
Fulvifomes rimosus (Berk.) Fiasson and N	Pistacia vera L.	41.578306	69.979688
Fulvifomes rimosus (Berk.) Fiasson and N	Quercus sp.	41.411662	70.035229
Fulvifomes rimosus (Berk.) Fiasson and N	Quercus trunk	42.171528°	60.105504°
Fulvifomes rimosus (Berk.) Fiasson and N	Quercus sp`.	39.576918	68.357755
Fulvifomes rimosus (Berk.) Fiasson and N	Salix sp.	41.340537	70.015082
Fulvifomes rimosus (Berk.) Fiasson and N	Salix sp.	40.552768	66.840521
Fulvifomes rimosus (Berk.) Fiasson and N	Populus sp.	40.597486	66.728903

Fulvifomes rimosus (Berk.) Fiasson and N	Populus sp.	38.885956	67.449038
Fulvifomes rimosus (Berk.) Fiasson and N	Quercus sp.	38.835752	67.407229
Fulvifomes robiniae (Murrill) Murrill.	Pistacia vera L.	38.8106	68.82403
Fulvifomes robiniae (Murrill) Murrill.	Pistacia vera L.	38.37733	69.29878
Fulvifomes robiniae (Murrill) Murrill.	Pistacia vera L.	38.37733	69.29878
Fulvifomes robiniae (Murrill) Murrill.	Pistacia vera L.	35.79807	61.45961
Fulvifomes robiniae (Murrill) Murrill.	Pistacia vera L.	37.83333	58
Fulvifomes robiniae (Murrill) Murrill.	Pistacia vera L.	35.29534	62.39485
Fulvifomes robiniae (Murrill) Murrill.	Pistacia vera L.	35.79807	61.45961
Fulvifomes robiniae (Murrill) Murrill.	Pistacia vera L.	35.79807	61.45961
Fulvifomes robiniae (Murrill) Murrill.	Pistacia vera L.	35.79807	61.45961
Fulvifomes robiniae (Murrill) Murrill.	Pistacia vera L.	35.79807	61.45961
Fulvifomes robiniae (Murrill) Murrill.	Pistacia vera L.	35.79807	61.45961
Fulvifomes robiniae (Murrill) Murrill.	Pistacia vera L.	35.80167	61.4675
Fulvifomes robiniae (Murrill) Murrill.	Pistacia vera L.	35.80167	61.4675
Fulvifomes robiniae (Murrill) Murrill.	Pistacia vera L.	35.80167	61.4675
Fulvifomes robiniae (Murrill) Murrill.	Pistacia vera L.	35.80167	61.4675
Fulvifomes robiniae (Murrill) Murrill.	Pistacia vera L.	35.80167	61.4675
Fulvifomes robiniae (Murrill) Murrill.	Pistacia vera L.	35.80167	61.4675
Fulvifomes robiniae (Murrill) Murrill.	Pistacia vera L.	35.26667	62.33333
Fulvifomes robiniae (Murrill) Murrill.	Pistacia vera L.	35.79807	61.45961
Fulvifomes robiniae (Murrill) Murrill.	Pistacia vera L.	35.77148	61.54134
Fulvifomes robiniae (Murrill) Murrill.	Pistacia vera L.	35.26667	62.3333
Fulvifomes robiniae (Murrill) Murrill.	Pistacia vera L.	35.79807	61.45961
Fulvifomes robiniae (Murrill) Murrill.	Pistacia vera L.	35.79807	61.45961
Fulvifomes robiniae (Murrill) Murrill.	Pistacia vera L.	35.79807	61.45961
Fulvifomes robiniae (Murrill) Murrill.	Pistacia vera L.	35.79807	61.45961
Fulvifomes robiniae (Murrill) Murrill.	Pistacia vera L.	35.79807	61.45961
Fulvifomes robiniae (Murrill) Murrill.	Pistacia vera L.	35.26667	62.33333
Fulvifomes robiniae (Murrill) Murrill.	Pistacia vera L.	35.79807	61.45961
Fulvifomes robiniae (Murrill) Murrill.	Pistacia vera L.	35.79807	61.45961
Fulvifomes robiniae (Murrill) Murrill.	Pistacia vera L.	35.79807	61.45961

Fulvifomes robiniae (Murrill) Murrill.	Pistacia vera L.		35.79807		61.45961
Fulvifomes robiniae (Murrill) Murrill.	Pistacia vera L.		35.26667		62.33333
Fulvifomes robiniae (Murrill) Murrill.	Pistacia vera L.		35.26667		62.33333
Fulvifomes robiniae (Murrill) Murrill.	Pistacia vera L.		35.79807		61.45961
Fomitiporia hartigii (Allesch. & Schnabl) F	Abies sibirica Ledeb.	50.616597222		83.360338889	
Fomitiporia hartigii (Allesch. & Schnabl) F	Abies sibirica Ledeb.	50.620452778		83.376422222	
Fomitiporia hartigii (Allesch. & Schnabl) F	Abies sibirica Ledeb.	49.975566667		82.953916667	
Fomitiporia hippophaeicola (H. Jahn) Fias	Hippophae rhamnoides L.	43.907041667		79.755897222	
Fomitiporia hippophaeicola (H. Jahn) Fias	Hippophae rhamnoides L.	43.114819444		76.913866667	
Fomitiporia hippophaeicola (H. Jahn) Fias	Hippophae rhamnoides L.	43.136388889		76.799166667	
Fomitiporia hippophaeicola (H. Jahn) Fias	Hippophae rhamnoides L.	43.158972222		77.033805556	
Fomitiporia hippophaeicola (H. Jahn) Fias	Betula tianschanica Rupr.	42.72936111		77.90425556	
Fomitiporia hippophaeicola (H. Jahn) Fias	Hippophae rhamnoides	42.72745833		77.89537222	
Fomitiporia hippophaeicola (H. Jahn) Fias	Hippophae rhamnoides	42.72867778		77.84062778	
Fomitiporia hippophaeicola (H. Jahn) Fias	Hippophae rhamnoides L.		38.43785		71.66734
Fomitiporia hippophaeicola (H. Jahn) Fias	Hippophae rhamnoides L.				
Fomitiporia hippophaeicola (H. Jahn) Fias	Hippophae rhamnoides L.		38.55075		71.75683
Fomitiporia hippophaeicola (H. Jahn) Fias	Hippophae rhamnoides L.	41.604614		69.907369	
Fomitiporia hippophaeicola (H. Jahn) Fias	Hippophae rhamnoides L.	41.360504		70.144653	
Fomitiporia hippophaeicola (H. Jahn) Fias	Hippophae rhamnoides L.	39.581746		67.168256	
Fomitiporia punctata (P. Karst.) Murrill	Crataegus songarica K.Koch	45.515869444		80.647775000	
Fomitiporia punctata (P. Karst.) Murrill	Salix wilhelmsiana M. Bieb.	43.573813889		79.314061111	
Fomitiporia punctata (P. Karst.) Murrill	Salix sp.	50.737825000		80.836041667	
Fomitiporia punctata (P. Karst.) Murrill	Salix sp.	54.785963889		69.485380556	
Fomitiporia punctata (P. Karst.) Murrill	Crataegus sanguinea Pall.	51.048566667		81.062180556	
Fomitiporia punctata (P. Karst.) Murrill	Crataegus songarica K.Koch	45.500622222		80.631738889	
Fomitiporia punctata (P. Karst.) Murrill	Crataegus songarica K.Koch	45.497777778		80.632477778	
Fomitiporia punctata (P. Karst.) Murrill	Ulmus sp.	41.216002		69.853039	
Fomitiporia punctata (P. Karst.) Murrill	Crataegus sp.	41.237125		69.925876	
Fomitiporia punctata (P. Karst.) Murrill	Crataegus azarolus var. pontica (K.Koch)	41.221857		69.963018	
Fomitiporia punctata (P. Karst.) Murrill	Populus sp.	39.665826		67.099841	
Fomitiporia punctata (P. Karst.) Murrill	Populus sp.	39.705381		68.333529	
Fomitiporia punctata (P. Karst.) Murrill	Betula sp.	40.518743		66.787254	
Fomitiporia punctata (P. Karst.) Murrill	Ulmus sp.	38.862482		67.432195	
Fomitiporia punctata (P. Karst.) Murrill	Populus sp.	41.863842°		60.886385°	
Fomitiporia robusta (P. Karst) Fiasson &	Hippophae rhamnoides L.	43.111011111		76.915530556	
Fomitiporia robusta (P. Karst) Fiasson &	Salix wilhelmsiana M. Bieb.	43.267147222		78.969966667	
Fomitiporia robusta (P. Karst) Fiasson	Spiraea crenata L.	52.406055556		63.912513889	

&			
Fomitiporia robusta (P. Karst) Fiasson &	Spiraea crenata L.	49.663333333	83.501044444
Fomitiporia robusta (P. Karst) Fiasson &	Spiraea hypericifolia L.	47.781266667	71.546866667
Fomitiporia robusta (P. Karst) Fiasson &	Spiraea hypericifolia L.	53.450050000	68.840955556
Fomitiporia robusta (P. Karst) Fiasson &	Spiraea hypericifolia L.	49.187250000	86.120186111
Fomitiporia robusta (P. Karst.) Fiasson an	Picea schrenkiana Fisch. & C.A.Mey.	42.71037222	77.69317222
Fomitiporia robusta (P. Karst.) Fiasson an	Hippophae rhamnoides L	42.80633333	77.45810000
Fomitiporia robusta (P. Karst.) Fiasson an	Hippophae rhamnoides L	42.59599167	78.10855556
Fomitiporia robusta (P. Karst.) Fiasson an	Hippophae rhamnoides L	41.34728889	72.94030278
Fomitiporia robusta (P. Karst.) Fiasson an	Hippophae rhamnoides L	40.75778056	73.43252222
Fomitiporia robusta (P. Karst.) Fiasson an	Hippophae rhamnoides L	42.85957778	74.58990556
Fomitiporia robusta (P. Karst.) Fiasson an	Hippophae rhamnoides L	42.71983889	74.75160000
Fomitiporia robusta (P. Karst.) Fiasson an	Hippophae rhamnoides L	42.69650556	77.66619444
Fomitiporia robusta (P. Karst) Fiasson &	Atraphaxis pyrifolia Bunge		
Fomitiporia robusta (P. Karst.) Fiasson an	Spiraea sp.	41.711582	69.987039
Fomitiporia robusta (P. Karst.) Fiasson an	Pistacia sp.	41.819611	70.317049
Fomitiporia robusta (P. Karst.) Fiasson an	Quercus sp.	41.393682	70.200427
Fomitiporia robusta (P. Karst.) Fiasson an	Morus alba L.	41.086842	71.47503
Fomitiporia robusta (P. Karst.) Fiasson an	Pistacia sp.	38.933682	66.865363
Fomitiporia robusta (P. Karst.) Fiasson an	Castanea sp.	38.924095	67.568576
Fomitiporia robusta (P. Karst.) Fiasson an	Quercus sp.	39.586722	68.276458
Fomitiporia robusta (P. Karst.) Fiasson an	Populus sp.	39.626491	68.493197
Fomitiporia robusta (P. Karst.) Fiasson an	Castanea sp.	40.490321	66.806928
Fomitiporia robusta (P. Karst.) Fiasson an	Juglans regia	41.086842	71.47503
Fuscoporia contigua (Pers.) G. Cunn.	Populus tremula L.	43.187861111	77.004072222
Fuscoporia contigua (Pers.) G. Cunn.	Hippophae rhamnoides L.	39.555741	68.368934
Fuscoporia contigua (Pers.) G. Cunn.	Acacia trunk	41.340537	70.015082
Fuscoporia contigua (Pers.) G. Cunn.	Acacia trunk	41.340538	70.015083

Fuscoporia contigua (Pers.) G. Cunn.	Alnus sp.	38.971221		67.414011	
Fuscoporia contigua (Pers.) G. Cunn.	Alnus sp.	40.552768		66.840521	
Fuscoporia contigua (Pers.) G. Cunn.	Ulmus sp.	39.652114		68.268427	
Fuscoporia contigua (Pers.) G. Cunn.	Ulmus sp.	42.076481°		60.395858°	
Fuscoporia ferruginosa (Schrad.) Murrill	Frangula alnus Mill.	50.676708333		80.864347222	
Fuscoporia ferruginosa (Schrad.) Murrill	Salix caprea L.	50.634219444		80.925247222	
Fuscoporia ferruginosa (Schrad.) Murrill	Salix turczaninowii Laksch.	49.187250000		86.120186111	
Fuscoporia ferruginosa (Schrad.) Murrill	Crataegus sanguinea Pall.	50.626275000		80.822366667	
Fuscoporia ferruginosa (Schrad.) Murrill	Juglans regia		38.80941		68.81765
Fuscoporia torulosa (Pers.) T. Wagner an	Fraxinus sogdiana Bunge	43.266422222		77.212138889	
Fuscoporia torulosa (Pers.) T. Wagner an	Malus domestica (Suckow) Borkh.	44.854280556		78.765152778	
Fuscoporia torulosa (Pers.) T. Wagner an	Malus sieversii (Ledeb.) M. Roem.	44.850238889		78.814669444	
Fuscoporia torulosa (Pers.) T. Wagner an	Salix wilhelmsiana M. Bieb.	43.576586111		79.324597222	
Fuscoporia torulosa (Pers.) T. Wagner an	Celtis caucasica Willd.	42.665133333		70.253366667	
Fuscoporia torulosa (Pers.) T. Wagner an	Crataegus chlorocarpa Lenné & K.Koch	49.518555556		85.048911111	
Fuscoporia torulosa (Pers.) T. Wagner an	Malus domestica (Suckow) Borkh.	44.866741667		78.766555556	
Fuscoporia torulosa (Pers.) T. Wagner an	Fraxinus sogdiana Bunge.	43.253755556		77.213886111	
Fuscoporia torulosa (Pers.) T. Wagner an	Fraxinus sogdiana Bunge.	43.529455556		79.274977778	
Fuscoporia torulosa (Pers.) T. Wagner an	Fraxinus sogdiana Bunge.	43.565186111		79.309013889	
Fuscoporia torulosa (Pers.) T. Wagner an	Fraxinus sogdiana Bunge.	43.597608333		79.336400000	
Fuscoporia torulosa (Pers.) T. Wagner an	Fraxinus sogdiana Bunge.	43.650433333		79.380500000	
Fuscoporia torulosa (Pers.) T. Wagner an	Malus sp.	42.71421944		78.04444722	
Fuscoporia torulosa (Pers.) T. Wagner an	Malus domestica (Suckow) Borkh	41.31900556		72.97307222	
Fuscoporia torulosa (Pers.) T. Wagner an	Prunus sp.	41.34512778		72.91889444	
Fuscoporia torulosa (Pers.) T. Wagner an	Prunus cerasifera	41.31847222		72.97677222	
Fuscoporia torulosa (Pers.) T. Wagner an	Quercus sp.	41.344954		69.316265	
Fuscoporia torulosa (Pers.) T. Wagner an	Quercus sp.	41.341868		69.311224	
Fuscoporia torulosa (Pers.) T. Wagner an	Betula tianschanica Rupr.	40.696952		72.075852	
Fuscoporia torulosa (Pers.) T. Wagner an	Salix babylonica L.	40.827958		72.31576	
Fuscoporia torulosa (Pers.) T. Wagner an	Pyrus communis L.	40.768512		72.394723	
Fuscoporia torulosa (Pers.) T. Wagner an	Morus nigra L.	40.766694		72.211579	
Hirschioporus abietinus (Pers. ex J.F. Gm	Salix caprea L.	50.737825000		80.836041667	
Hirschioporus abietinus (Pers. ex J.F. Gm	Picea schrenkiana Fisch. & Mey.	43.161038889		77.048450000	
Hirschioporus abietinus (Pers. ex J.F. Gm	Picea schrenkiana Fisch. & Mey.	43.123913889		77.079638889	
Hirschioporus abietinus (Pers. ex J.F. Gm	Abies sibirica Ledeb.	50.616597222		83.360338889	
Hirschioporus abietinus (Pers. ex J.F. Gm	Abies sibirica Ledeb.	50.620452778		83.376422222	
Hirschioporus abietinus (Pers. ex J.F. Gm	Abies sibirica Ledeb.	50.451458333		83.447233333	
Hirschioporus abietinus (Pers. ex J.F. Gm	Abies sibirica Ledeb.	50.403980556		83.402472222	
Hirschioporus abietinus (Pers. ex J.F. Gm	Picea schrenkiana Fisch & C.A.	43.120033333		77.078727778	

	Mey.				
Hirschioporus abietinus (Pers. ex J.F. Gm	Picea schrenkiana Fisch & C.A. Mey.	43.112694444		77.079361111	
Hirschioporus abietinus (Pers. ex J.F. Gm	Picea schrenkiana Fisch & C.A. Mey.	43.107347222		77.074375000	
Hirschioporus abietinus (Pers. ex J.F. Gm	Picea schrenkiana Fisch & C.A. Mey.	43.102416667		77.075580556	
Hirschioporus abietinus (Pers. ex J.F. Gm	Picea schrenkiana Fisch & C.A. Mey.	45.329583333		80.237802778	
Hirschioporus abietinus (Pers. ex J.F. Gm	Pinus sylvestris L.	52.649672222		70.494180556	
Hirschioporus abietinus (Pers. ex J.F. Gm	Pinus sylvestris L.	52.523052778		68.798002778	
Hirschioporus abietinus (Pers. ex J.F. Gm	Pinus sylvestris L.	50.737825000		80.836041667	
Hirschioporus abietinus (Pers. ex J.F. Gm	Abies sibirica Ledeb.	50.368511111		83.954577778	
Hirschioporus abietinus (Pers. ex J.F. Gm	Picea schrenkiana Fisch. & Mey.	42.45463611		78.52848056	
Hirschioporus abietinus (Pers. ex J.F. Gm	Picea schrenkiana Fisch. & Mey.	42.42336389		78.55023333	
Hirschioporus abietinus (Pers. ex J.F. Gm	Picea schrenkiana Fisch. & Mey.	42.47650556		78.55911389	
Hirschioporus abietinus (Pers. ex J.F. Gm	Picea schrenkiana Fisch. & Mey.	42.44605833		78.56400278	
Hirschioporus abietinus (Pers. ex J.F. Gm	Picea schrenkiana Fisch. & Mey.	42.43548611		78.54508056	
Hirschioporus abietinus (Pers. ex J.F. Gm	Picea schrenkiana Fisch. & Mey.	41.42640833		78.57923333	
Hirschioporus abietinus (Pers. ex J.F. Gm	Picea schrenkiana Fisch. & Mey.	42.46467778		78.56001389	
Hirschioporus abietinus (Pers. ex J.F. Gm	Pinus sylvestris L.	42.47385000		78.48830278	
Hirschioporus abietinus (Pers. ex J.F. Gm	Pinus sp.	41.156327		69.844872	
Hirschioporus abietinus (Pers. ex J.F. Gm	Pinus sp.	40.526749		66.700184	
Hirschioporus abietinus (Pers. ex J.F. Gm	Pinus sp.	38.88881		67.458935	
Hirschioporus fuscoviolaceus (Ehrenb.) D	Abies sibirica Ledeb.	50.451458333		83.447233333	
Hirschioporus fuscoviolaceus (Ehrenb.) D	Pinus sibirica Du Tour	49.208261111		86.347266667	
Hirschioporus fuscoviolaceus (Ehrenb.) D	Pinus sylvestris L.	52.436263889		64.031066667	
Hirschioporus fuscoviolaceus (Ehrenb.) D	Abies sibirica Ledeb	41.255551		69.8442	
Hirschioporus fuscoviolaceus (Ehrenb.) D	Pinus sp.	41.156327		69.844872	
Hirschioporus tianschanicus Y.C. Dai, Yua	Larix sp.	41.36055278		72.97123333	
Hirschioporus tianschanicus Y.C. Dai, Yua	Picea sp.	41.22578056		73.27512778	
Inocutis dryophila (Berk.) Fiasson & Niem	Acer negundo L.		37.915		58.08987
Inocutis dryophila (Berk.) Fiasson & Niem	Acer negundo L.		37.915		58.08987
Inocutis rheades (Pers.) Fiasson & Nieme	Populus euphratica Olivier	44.171694444		76.951666667	
Inocutis rheades (Pers.) Fiasson & Nieme	Betula pendula Roth.	45.411150000		80.386786111	
Inocutis rheades (Pers.) Fiasson & Nieme	Populus tremula L.	52.997619444		64.114108333	
Inocutis rheades (Pers.) Fiasson & Nieme	Populus tremula L.	53.790563889		64.129466667	
Inocutis rheades (Pers.) Fiasson & Nieme	Populus tremula L.	52.406055556		63.912513889	
Inocutis rheades (Pers.) Fiasson & Nieme	Populus tremula L.	52.379180556		69.155638889	
Inocutis rheades (Pers.) Fiasson & Nieme	Populus tremula L.	50.676708333		80.860041667	
Inocutis rheades (Pers.) Fiasson & Nieme	Populus tremula L.	50.676708333		80.864347222	
Inocutis rheades (Pers.) Fiasson & Nieme	Betula pendula Roth	45.411150000		80.386786111	
Inocutis rheades (Pers.) Fiasson & Nieme	Betula pubescens Ehrh.	50.634219444		80.925247222	
Inocutis tamaricis (Pat.) Fiasson and Niem	Tamarix sp. T. pallasii, on a trunk	43.267147222		78.966998056	
Inocutis tamaricis (Pat.) Fiasson and Niem	Tamarix sp.	43.891444444		77.099561111	
Inocutis tamaricis (Pat.) Fiasson and Niem	Tamarix sp.	44.284358333		79.833611111	
Inocutis tamaricis (Pat.) Fiasson and Niem	Tamarix sp.	73.675075000		73.676666667	
Inocutis tamaricis (Pat.) Fiasson and Niem	Tamarix sp.	41.383013889		69.041372222	

Inocutis tamaricis (Pat.) Fiasson and Niem	Tamarix sp.	43.546722222		79.297516667	
Inocutis tamaricis (Pat.) Fiasson and Niem	Tamarix sp.	43.584511111		79.331394444	
Inocutis tamaricis (Pat.) Fiasson and Niem	Tamarix ramosissima Ledeb.	43.920980556		77.089302778	
Inocutis tamaricis (Pat.) Fiasson and Niem	Tamarix ramosissima Ledeb.	43.604122222		79.357105556	
Inocutis tamaricis (Pat.) Fiasson and Niem	Tamarix ramosissima Ledeb.	43.918830556		77.096322222	
Inocutis tamaricis (Pat.) Fiasson and Niem	Tamarix ramosissima Ledeb.	43.915672222		77.098430556	
Inocutis tamaricis (Pat.) Fiasson and Niem	Tamarix ramosissima Ledeb.	43.763819444		73.760494444	
Inocutis tamaricis (Pat.) Fiasson and Niem	Tamarix ramosissima Ledeb.	45.431797222		73.671766667	
Inocutis tamaricis (Pat.) Fiasson and Niem	Tamarix sp.		38.566667		56.733333
Inocutis tamaricis (Pat.) Fiasson and Niem	Tamarix sp.		37.95194444		58.34694444
Inocutis tamaricis (Pat.) Fiasson and Niem	Tamarix sp.		39.6275		54.179722
Inocutis tamaricis (Pat.) Fiasson and Niem	Tamarix sp.		38.566667		56.733333
Inocutis tamaricis (Pat.) Fiasson and Niem	Tamarix sp.		38.566667		56.733333
Inocutis tamaricis (Pat.) Fiasson and Niem	Tamarix sp.	37.297119		67.16691	
Inocutis tamaricis (Pat.) Fiasson and Niem	Tamarix hispida Willd.	42.163482		63.557148	
Inocutis tamaricis (Pat.) Fiasson and Niem	Tamarix ramosissima Ledeb.	42.162431		63.526068	
Inocutis tamaricis (Pat.) Fiasson and Niem	Tamarix sp.		42.734882		59.724134
Inocutis tamaricis (Pat.) Fiasson and Niem	Tamarix hispida	39.665554		67.100094	
Inocutis tamaricis (Pat.) Fiasson and Niem	Tamarix hispida	42.20295		63.549253	
Inonotus andersonii (Ellis and Everh.) ˇC	Quercus sp.	41.379431		70.187456	
Inonotus andersonii (Ellis and Everh.) ˇC	Quercus sp.	41.440175		70.119916	
Inonotus andersonii (Ellis and Everh.) ˇC	Quercus sp.	38.893808		67.451334	
Inonotus cuticularis (Bull.) P. Karst.	Juglans regia	38.898646		67.447494	
Inonotus hispidus (Bull.) P. Karst.	Populus macrocarpa (Schrenk) Pavlov &	43.259558333		76.966802778	
Inonotus hispidus (Bull.) P. Karst.	Juglans regia L.	42.151808333		70.418925000	
Inonotus hispidus (Bull.) P. Karst.	Ulmus minor subsp. minor	43.226925000		76.925980556	
Inonotus hispidus (Bull.) P. Karst.	Ulmus pumila L.	46.854963889		74.946347222	
Inonotus hispidus (Bull.) P. Karst.	Celtis caucasica Willd.	42.771797222		69.622966667	
Inonotus hispidus (Bull.) P. Karst.	Morus alba L.	43.497469444		52.224991667	
Inonotus hispidus (Bull.) P. Karst.	Morus alba L.	43.259602778		76.950402778	
Inonotus hispidus (Bull.) P. Karst.	Morus alba L.	43.257647222		76.956013889	
Inonotus hispidus (Bull.) P. Karst.	Malus domestica (Suckow) Borkh.	45.506847222		80.600811111	
Inonotus hispidus (Bull.) P. Karst.	Malus domestica (Suckow) Borkh.	45.508988889		80.628508333	
Inonotus hispidus (Bull.) P. Karst.	Malus domestica (Suckow) Borkh.	43.328536111		77.611622222	
Inonotus hispidus (Bull.) P. Karst.	Malus domestica (Suckow) Borkh.	43.341250000		77.608786111	
Inonotus hispidus (Bull.) P. Karst.	Malus domestica (Suckow) Borkh.	43.112694444		77.075261111	
Inonotus hispidus (Bull.) P. Karst.	Malus domestica (Suckow) Borkh.	43.207361111		77.013866667	
Inonotus hispidus (Bull.) P. Karst.	Malus domestica (Suckow) Borkh.	43.298669444		77.266277778	
Inonotus hispidus (Bull.) P. Karst.	Tamarix sp.	42.440972222		68.780752778	
Inonotus hispidus (Bull.) P. Karst.	Fraxinus sogdiana Bunge.	43.212713889		76.882127778	
Inonotus hispidus (Bull.) P. Karst.	Malus domestica (Suckow) Borkh.	45.508988889		80.628508333	
Inonotus hispidus (Bull.) P. Karst.	Malus domestica (Suckow) Borkh.	45.506847222		80.600811111	
Inonotus hispidus (Bull.) P. Karst.	Malus sp.	41.31675833		72.96231389	
Inonotus hispidus (Bull.) P. Karst.	Malus sp.	41.67348333		72.94678056	
Inonotus hispidus (Bull.) P. Karst.	Juglans regia L.	41.34330000		72.90564722	
Inonotus hispidus (Bull.) P. Karst.	Juglans regia L.	42.45718889		78.52377778	

Inonotus hispidus (Bull.) P. Karst.	Juglans regia L.	41.87979167	71.98325556
Inonotus hispidus (Bull.) P. Karst.	Ulmus sp.	40.51620278	72.82292500
Inonotus hispidus (Bull.) P. Karst.	Ulmus sp.	41.88630000	71.97250000
Inonotus hispidus (Bull.) P. Karst.	Populis alba	41.34303611	72.91351667
Inonotus hispidus (Bull.) P. Karst.	Junglas regia	42.81050278	74.66097500
Inonotus hispidus (Bull.) P. Karst.	Acer sp	41.33395556	72.92203611
Inonotus hispidus (Bull.) P. Karst.	Acer sp	42.83229722	74.63515556
Inonotus hispidus (Bull.) P. Karst.	Morus alba L.	37.95	58.38333
Inonotus hispidus (Bull.) P. Karst.	Salix	37.91972	58.09357
Inonotus hispidus (Bull.) P. Karst.	Morus alba L.	37.97246	58.01817
Inonotus hispidus (Bull.) P. Karst.	Salix	37.915	58.08987
Inonotus hispidus (Bull.) P. Karst.	Morus alba L.	37.95	58.33333
Inonotus hispidus (Bull.) P. Karst.	Platanus L.	38.403	56.757
Inonotus hispidus (Bull.) P. Karst.	Asclepias syriaca L.	38.06667	57.36667
Inonotus hispidus (Bull.) P. Karst.	Acer negundo L.	37.915	58.08987
Inonotus hispidus (Bull.) P. Karst.	Acer negundo L.	37.915	58.08987
Inonotus hispidus (Bull.) P. Karst.	Juglans regia	41.658092	69.956647
Inonotus hispidus (Bull.) P. Karst.	Juglans regia	41.658331	69.768431
Inonotus hispidus (Bull.) P. Karst.	Juglans regia	41.670924	69.751136
Inonotus hispidus (Bull.) P. Karst.	Pinus sp.	41.912415	70.388667
Inonotus hispidus (Bull.) P. Karst.	Pinus sp.	41.971423	70.491884
Inonotus hispidus (Bull.) P. Karst.	Malus sieversii	41.611246	69.914891
Inonotus hispidus (Bull.) P. Karst.	on dried trunk angiosperm wood	41.342867	69.31547
Inonotus hispidus (Bull.) P. Karst.	trunk angiosperm wood	41.33084	69.350979
Inonotus hispidus (Bull.) P. Karst.	Juglans regia	41.952365	70.437388
Inonotus hispidus (Bull.) P. Karst.	Morus alba	41.154266	70.103151
Inonotus hispidus (Bull.) P. Karst.	Juglans regia	38.240265	67.311687
Inonotus hispidus (Bull.) P. Karst.	Morus alba	41.33084	69.350979
Inonotus hispidus (Bull.) P. Karst.	Juglans regia	38.317435	67.049691
Inonotus hispidus (Bull.) P. Karst.	Prunus avium (L.) L.	41.530085	69.9481
Inonotus hispidus (Bull.) P. Karst.	Malus domestica Borkh.	41.3775	64.5853
Inonotus iliensis Kravtzev.	Populus macrocarpa (Schrenk) Pavlov &	43.546030556	79.287444444
Inonotus iliensis Kravtzev.	Populus euphratica Olivier	43.586694444	79.320027778
Inonotus iliensis Kravtzev	Populus sp.	43.918425000	77.097283333
Inonotus iliensis Kravtzev	Juglans regia L.	41.34134167	72.93988333
Inonotus iliensis Kravtzev	Morus alba L.	41.34039444	72.93986667
Inonotus iliensis Kravtzev	Ulmus campestris L.	41.34040000	72.94011111
Inonotus obliquus (Ach. ex Pers.) Pilat	Malus domestica (Suckow) Borkh.	43.175527778	77.033388889
Inonotus obliquus (Ach. ex Pers.) Pilat	Malus domestica (Suckow) Borkh.	45.878333333	81.195083333
Inonotus obliquus (Ach. ex Pers.) Pilat	Malus domestica (Suckow) Borkh.	45.681916667	80.693527778
Inonotus obliquus (Ach. ex Pers.) Pilat	Malus domestica (Suckow) Borkh.	43.095244444	76.906747222
Inonotus obliquus (Ach. ex Pers.) Pilat	Betula pendula Roth	52.436263889	64.031066667

Inonotus obliquus (Ach. ex Pers.) Pilat	Betula pendula Roth	53.790572222	64.129466667
Inonotus obliquus (Ach. ex Pers.) Pilat	Betula pendula Roth	50.737825000	80.836041667
Inonotus obliquus (Ach. ex Pers.) Pilat	Betula pendula Roth	49.149275000	85.602138889
Inonotus obliquus (Fr.) Pilát	on trunk of angiosperm wood	42.43607222	78.41279167
Inonotus obliquus (Fr.) Pilát	unknown angiosperm fallen trunk	41.345009	69.313287
Inonotus obliquus (Fr.) Pilát	Betula sp.	41.453992	70.206016
Inonotus obliquus (Fr.) Pilát	Fraxinus sp.	38.875354	67.433195
Inonotus obliquus (Fr.) Pilát	Alnus sp.	40.600013	66.666441
Inonotus obliquus (Fr.) Pilát	Salix sp.	39.650255	68.378643
Inonotus obliquus (Fr.) Pilát	Salix sp.	41.645437°	60.838674°
Inonotus obliquus (Fr.) Pilát	Salix sp.	40.720028	72.438431
Inonotus pseudohispidus Kravtzev	Populus macrocarpa (Schrenk) Pavlov &	43.546030556	79.287444444
Inonotus pseudohispidus Kravtzev	Populus pruinosa Schrenk	41.600140°	60.914193°
Inonotus pseudohispidus Kravtzev	Populus euphratica Oliv.	41.600140°	60.914193°
Inonotus pseudohispidus Kravtzev	Populus sp.	39.623794	67.18006
Inonotus pseudohispidus Kravtzev	Populus sp.	41.600140°	60.914193°
Inonotus pseudohispidus Kravtzev	Populus sp.	39.623794	67.18006
Inonotus pseudohispidus Kravtzev	Populus alba	39.623794	67.18006
Inonotus pseudohispidus Kravtzev	Fraxinus sogdiana Bunge.	43.586694444	79.321180556
Mensularia radiata (Sowerby) Lázaro Ibiz	Prunus cerasus L.	43.244472222	76.713663889
Mensularia radiata (Sowerby) Lázaro Ibiz	Quercus sp.	41.381794	70.161811
Mensularia radiata (Sowerby) Lázaro Ibiz	Alnus tree	40.721689	71.537197
Mensularia radiata (Sowerby) Lázaro Ibiz	angiosperm woody plants	40.528413	66.778917
Mensularia radiata (Sowerby) Lázaro Ibiz	Ulmus sp.	39.749233	68.332821
Mensularia radiata (Sowerby) Lázaro Ibiz	Quercus sp.	38.876436	67.430939
Mensularia radiata (Sowerby) Lázaro Ibiz	Ulmus sp.	39.637033	67.157363
Onnia tomentosa (Fr.) P. Karst.	ash and conifer tree	50.001797222	73.139358333
Pallidohirschioporus biformis (Fr.) Y.C. D	Betula pendula Roth.	52.449372222	64.070527778
Pallidohirschioporus biformis (Fr.) Y.C. D	Betula pendula Roth.	53.795394444	64.138147222
Pallidohirschioporus biformis (Fr.) Y.C. D	Betula pendula Roth.	54.025227778	65.304847222
Pallidohirschioporus biformis (Fr.) Y.C. D	Betula pendula Roth.	52.653075000	70.492105556
Pallidohirschioporus biformis (Fr.) Y.C. D	Betula pendula Roth.	52.968575000	70.266394444
Pallidohirschioporus biformis (Fr.) Y.C. D	Betula pendula Roth.	54.881133333	69.915894444
Pallidohirschioporus biformis (Fr.)	Betula pendula Roth.	55.107197222	68.956222222

Y.C. D					
Pallidohirschioporus biformis (Fr.) Y.C. D	Betula pendula Roth.	55.157175000		69.039494444	
Pallidohirschioporus biformis (Fr.) Y.C. D	Betula pendula Roth.	52.836186111		69.512188889	
Pallidohirschioporus biformis (Fr.) Y.C. D	Betula pendula Roth.	49.975566667		82.953916667	
Pallidohirschioporus biformis (Fr.) Y.C. D	Betula pendula Roth.	49.817211111		84.299947222	
Pallidohirschioporus biformis (Fr.) Y.C. D	dried woody plants	41.629161		69.748752	
Pallidohirschioporus biformis (Fr.) Y.C. D	trunk of angiosperms tree	41.545635		70.02759	
Pallidohirschioporus biformis (Fr.) Y.C. D	Populus sp.	41.335779		70.231662	
Pallidohirschioporus biformis (Fr.) Y.C. D	Salix sp.	38.894023		67.477363	
Pallidohirschioporus biformis (Fr.) Y.C. D	Populus sp.	41.600140°		60.914193°	
Pallidohirschioporus biformis (Fr.) Y.C. D	Salix sp.	39.630542		67.147106	
Phellinidium ferrugineofuscum (P. Karst.)	Larix gmelinii var. gmelinii		38.393		56.724
Phellinidium ferrugineofuscum (P. Karst.)	Pinus sp.	41.364597		70.171236	
Phellinidium ferrugineofuscum (P. Karst.)	Pinus sp.	39.607335		68.289323	
Phellinidium ferrugineofuscum (P. Karst.)	Picea sp.	38.90576		66.832587	
Phellinidium ferrugineofuscum (P. Karst.)	Picea sp.	38.892345		67.464724	
Phellinopsis conchata (Pers.) Y.C. Dai	Picea schrenkiana Fisch. & C.A. Mey.	43.150133333		77.048511111	
Phellinopsis conchata (Pers.) Y.C. Dai	Populus tremula L.	43.169633333		77.034252778	
Phellinopsis conchata (Pers.) Y.C. Dai	Salix capusii Franch.	43.061016667		77.051283333	
Phellinopsis conchata (Pers.) Y.C. Dai	Salix capusii Franch.	43.169633333		77.034252778	
Phellinopsis conchata (Pers.) Y.C. Dai	Salix lanata subsp. lanata	45.837363889		79.661419444	
Phellinopsis conchata (Pers.) Y.C. Dai	Salix lanata subsp. lanata	45.359425000		79.929747222	
Phellinopsis conchata (Pers.) Y.C. Dai	Salix tenuijulis Ledeb.	43.176738889		77.095902778	
Phellinopsis conchata (Pers.) Y.C. Dai	Salix lanata subsp. lanata	43.171677778		77.111705556	
Phellinopsis conchata (Pers.) Y.C. Dai	Syringa vulgaris L.	43.225269444		76.899786111	
Phellinopsis conchata (Pers.) Y.C. Dai	Syringa vulgaris L.	43.225075000		76.914219444	
Phellinopsis conchata (Pers.) Y.C. Dai	Salix tenuijulis Ledeb.	43.171677778		77.111705556	
Phellinopsis conchata (Pers.) Y.C. Dai	Salix triandra L.	43.121700000		76.911961111	
Phellinopsis conchata (Pers.) Y.C. Dai	Salix triandra L.	43.095230556		76.907119444	
Phellinopsis conchata (Pers.) Y.C. Dai	Salix triandra L.	43.157627778		77.054505556	
Phellinopsis conchata (Pers.) Y.C. Dai	Salix triandra L.	43.157422222		77.032297222	
Phellinopsis conchata (Pers.) Y.C. Dai	Salix bebbiana Sarg.	45.359425000		79.929747222	
Phellinopsis conchata (Pers.) Y.C. Dai	on trunk of angiosperm wood	42.45770000		78.54108333	
Phellinopsis conchata (Pers.) Y.C. Dai	on trunk of angiosperm wood	40.79925556		73.61593889	
Phellinopsis conchata (Pers.) Y.C.Dai	Rosa sp.		38.403		56.757

Phellinopsis conchata (Pers.) Y.C.Dai	Juniperus polycarpos var. turcomanica (B	38.63994	59.47209
Phellinopsis conchata (Pers.) Y.C. Dai	Syringa sp.	41.331678	70.382258
Phellinopsis conchata (Pers.) Y.C. Dai	Populus sp.	40.502945	66.775281
Phellinopsis conchata (Pers.) Y.C. Dai	Alnus sp.	40.311885	71.932708
Phellinopsis conchata (Pers.) Y.C. Dai	Ulmus sp.	39.640836	67.154658
Phellinopsis conchata (Pers.) Y.C. Dai	Populus sp.	37.92666	66.755786
Phellinopsis conchata (Pers.) Y.C. Dai	Alnus sp.	38.872433	67.422288
Phellinopsis conchata (Pers.) Y.C. Dai	Ulmus sp.	39.585089	67.166918
Phellinus betulinus (Murrill) Parmasto	Betula tianschanica	41.567041	70.049129
Phellinus betulinus (Murrill) Parmasto	Betula tianschanica	41.56704101	70.04912853
Phellinus igniarius (L.) Quél.	Salix sp.	43.110213889	76.916952778
Phellinus igniarius (L.) Quél.	Salix sp.	43.112944444	76.915130556
Phellinus igniarius (L.) Quél.	Prunus padus L.	43.240247222	77.764883333
Phellinus igniarius (L.) Quél.	Prunus padus L.	43.101913889	76.907341667
Phellinus igniarius (L.) Quél.	Salix starkeana Willd.	43.105475000	76.920855556
Phellinus igniarius (L.) Quél.	Salix starkeana Willd.	44.724644444	78.959780556
Phellinus igniarius (L.) Quél.	Salix starkeana Willd.	44.497988889	78.889827778
Phellinus igniarius (L.) Quél.	Salix starkeana Willd.	43.304388889	79.512333333
Phellinus igniarius (L.) Quél.	Populus alba L.	49.521850000	85.418994444
Phellinus igniarius (L.) Quél.	Ulmus laevis Pall.	51.416911111	52.618600000
Phellinus igniarius (L.) Quél.	Prunus padus L.	43.093413889	76.906611111
Phellinus igniarius (L.) Quél.	Betula pendula Roth	52.436263889	64.031066667
Phellinus igniarius (L.) Quél.	Betula pendula Roth	53.790572222	64.129466667
Phellinus igniarius (L.) Quél.	Betula pendula Roth	52.523052778	68.798002778
Phellinus igniarius (L.) Quél.	Betula pendula Roth	51.912497222	78.728958333
Phellinus igniarius (L.) Quél.	Betula pendula Roth	51.048566667	81.062180556
Phellinus igniarius (L.) Quél.	Betula pendula Roth	51.036886111	81.047402778
Phellinus igniarius (L.) Quél.	Betula pendula Roth	50.394500000	84.215194444
Phellinus igniarius (L.) Quél.	Betula pendula Roth	49.187250000	86.120186111
Phellinus igniarius (L.) Quél.	Betula pubescens Ehrh.	52.436263889	64.031066667
Phellinus igniarius (L.) Quél.	Betula pubescens Ehrh.	53.790572222	EO64°07'46.08"
Phellinus igniarius (L.) Quél.	Betula pubescens Ehrh.	52.523052778	68.798002778
Phellinus igniarius (L.) Quél.	Betula pubescens Ehrh.	45.769286111	81.452188889
Phellinus igniarius (L.) Quél.	Salix acutifolia Willd.	51.036886111	81.047402778
Phellinus igniarius (L.) Quél.	Salix alba L.	51.403636111	52.562094444
Phellinus igniarius (L.) Quél.	Salix alba L.	51.433763889	52.562094444
Phellinus igniarius (L.) Quél.	Salix caprea L.	45.359425000	79.929747222
Phellinus igniarius (L.) Quél.	Salix starkeana Willd.	43.086550000	76.908380556
Phellinus igniarius (L.) Quél.	Salix starkeana Willd.	44.943711111	78.722730556
Phellinus igniarius (L.) Quél.	Salix songarica Andersson	45.769286111	81.452188889
Phellinus igniarius (L.) Quél.	Salix tenuijulis Ledeb.	47.070686111	82.745736111
Phellinus igniarius (L.) Quél.	Salix tenuijulis Ledeb.	44.162869444	79.981213889
Phellinus igniarius (L.) Quél.	Salix turanica Nasarow	43.315877778	78.515825000
Phellinus igniarius (L.) Quél.	Salix turanica Nasarow	43.529455556	79.274977778
Phellinus igniarius (L.) Quél.	Salix wilhelmsiana M.Bieb.	43.565186111	79.309013889

Phellinus igniarius (L.) Quél.	Salix wilhelmsiana M.Bieb.	43.597608333	79.336400000
Phellinus igniarius (L.) Quél.	Salix wilhelmsiana M.Bieb.	43.650433333	79.380500000
Phellinus igniarius (L.) Quél.	Salix triandra L.	50.737825000	80.836041667
Phellinus igniarius (L.) Quél.	Betula pendula Roth	52.366958333	69.155638889
Phellinus igniarius (L.) Quél.	Picea schrenkiana Fisch. & C.A.Mey.	42.79884167	77.52559722
Phellinus igniarius (L.) Quél.	Juglans regia L.	41.33441111	72.98244167
Phellinus igniarius (L.) Quél.	Juglans regia L.	43.97500000	77.08146389
Phellinus igniarius (L.) Quél.	Betula tianschanica Rupr.	41.34091944	72.94514722
Phellinus igniarius (L.) Quél.	Betula tianschanica Rupr.	40.66660833	73.13592500
Phellinus igniarius (L.) Quél.	Betula sp.	41.31558611	72.91150556
Phellinus igniarius (L.) Quél.	Prunus amygdalus Batsch	42.84487778	74.63584167
Phellinus igniarius (L.) Quél.	Prunus spinosissima (Bunge) Franch.	40.58327778	73.13589167
Phellinus igniarius (L.) Quél.	trunk of angiosperm	42.40667500	78.52435556
Phellinus igniarius (L.) Quél.	Salix sp.	41.567041	70.049129
Phellinus igniarius (L.) Quél.	Juglans regia	41.595669	70.028679
Phellinus igniarius (L.) Quél.	Juglans regia	41.599430	70.015599
Phellinus igniarius (L.) Quél.	Prunus vulgaris	41.546298	70.018742
Phellinus igniarius (L.) Quél.	Juglans regia	40.719433	72.434532
Phellinus igniarius (L.) Quél.	Prunus sp.	39.616822	67.189748
Phellinus igniarius (L.) Quél.	Acer sp.	41.502320°	61.055617°
Phellinus igniarius (L.) Quél.	Acer sp.	41.138986	71.46405
Phellinus igniarius (L.) Quél.	Salix sp.	39.75124	68.321108
Phellinus igniarius (L.) Quél.	Salix sp.	40.592993	66.730594
Phellinus igniarius (L.) Quél.	Salix sp.	38.898849	67.448065
Phellinus igniarius (L.) Quél.	Juglans regia	40.722115	72.437248
Phellinus nigricans (Fr.) P. Karst		41.33752222	72.99156944
Phellinus nigricans (Fr.) P. Karst		41.30636667	72.92321667
Phellinus nigricans (Fr.) P. Karst		41.32186389	73.01587778
Phellinus nigricans (Fr.) P. Karst		41.21280000	73.32592222
Phellinus nigricans (Fr.) P. Karst		42.20146667	73.49258889
Phellinus igniarius (L.) Quél.	Salix sp.	38.83877	70.86195
Phellinus igniarius (L.) Quél.	Salix sp.	38.83877	70.86195
Phellinus igniarius (L.) Quél.	Salix sp.	38.79	69.36
Phellinus igniarius (L.) Quél.	Salix sp.	37.58177	71.5139
Phellinus igniarius (L.) Quél.	Salix sp.	37.58177	71.5139
Phellinus igniarius (L.) Quél.	Salix sp.	37.58177	71.5139
Phellinus igniarius (L.) Quél.	Salix sp.	37.58177	71.5139
Phellinus igniarius (L.) Quél.	Salix sp.	37.58177	71.5139
Phellinus igniarius (L.) Quél.	Salix sp.	37.58177	71.5139
Phellinus igniarius (L.) Quél.	Salix sp.	37.58177	71.5139
Phellinus igniarius (L.) Quél.	Salix sp.	37.58177	71.5139
Phellinus igniarius (L.) Quél.	Salix sp.	37.58177	71.5139
Phellinus igniarius (L.) Quél.	Alnus glutinosa (L.) Gaertn.	37.58177	71.5139
Phellinus pomaceus (Pers.) Maire	Prunus cerasifera Ehrh.	42.105202778	70.409272222
Phellinus pomaceus (Pers.) Maire	Prunus cerasifera Ehrh.	41.720986111	69.815358333

Phellinus pomaceus (Pers.) Maire	Prunus spinosa L.	51.405286111	52.561816667
Phellinus pomaceus (Pers.) Maire	Malus domestica	42.319808333	70.364716667
Phellinus pomaceus (Pers.) Maire	Prunus mahaleb L.	44.652422222	70.367516667
Phellinus pomaceus (Pers.) Maire	Prunus mahaleb L.	41.720986111	69.815358333
Phellinus pomaceus (Pers.) Maire	Prunus cerasifera Ehrh.	41.87751944	71.98300000
Phellinus pomaceus (Pers.) Maire	Prunus cerasifera Ehrh.	41.33296111	71.97963333
Phellinus pomaceus (Pers.) Maire	Prunus cerasifera Ehrh.	41.90590556	71.96932222
Phellinus pomaceus (Pers.) Maire	Prunus cerasifera Ehrh.	41.87684444	71.93761111
Phellinus pomaceus (Pers.) Maire	Lonicera sp.	41.90577500	71.95348056
Phellinus pomaceus (Pers.) Maire	Juglans regia L.	41.91055000	71.95840556
Phellinus pomaceus (Pers.) Maire	Malus sp.	41.90108333	71.96396944
Phellinus pomaceus (Pers.) Maire	Prunus sp.	41.66604444	72.11451944
Phellinus pomaceus (Pers.) Maire	Prunus sp.	41.67778611	72.08240556
Phellinus pomaceus (Pers.) Maire	Lonicera sp.	41.18676111	72.91715000
Phellinus pomaceus (Pers.) Maire	Prunus sp.	38.8106	68.82403
Phellinus pomaceus (Pers.) Maire	Prunus sp.	38.39078254	71.43919945
Phellinus pomaceus (Pers.) Maire	Prunus cerasifera Ehrh.	38.8042	68.8075
Phellinus pomaceus (Pers.) Maire	Prunus cerasifera Ehrh.	38.8042	68.8075
Phellinus pomaceus (Pers.) Maire	Prunus cerasifera Ehrh.	38.79076	69.35901
Phellinus pomaceus (Pers.) Maire	Prunus cerasifera Ehrh.	38.79076	69.35901
Phellinus pomaceus (Pers.) Maire	Prunus cerasifera Ehrh.	38.79076	69.35901
Phellinus pomaceus (Pers.) Maire	Prunus bucharica (Korsh.) Hand.-Mazz.	38.79076	69.35901
Phellinus pomaceus (Pers.) Maire	Prunus cerasifera Ehrh.	38.79076	69.35901
Phellinus pomaceus (Pers.) Maire	Prunus cerasifera Ehrh.	38.79076	69.35901
Phellinus pomaceus (Pers.) Maire	Prunus cerasifera Ehrh.	38.79076	69.35901
Phellinus pomaceus (Pers.) Maire	Prunus cerasifera Ehrh.	38.79076	69.35901
Phellinus pomaceus (Pers.) Maire	Prunus cerasifera Ehrh.	38.79076	69.35901
Phellinus pomaceus (Pers.) Maire	Prunus sp.	39.15112	69.19204
Phellinus pomaceus (Pers.) Maire	Prunus microcarpa C.A.Mey.	38.403	56.757
Phellinus pomaceus (Pers.) Maire	Prunus microcarpa C.A.Mey.	38.403	56.757
Phellinus pomaceus (Pers.) Maire	Prunus microcarpa C.A.Mey.	38.403	56.757
Phellinus pomaceus (Pers.) Maire	Prunus microcarpa C.A.Mey.	38.403	56.757
Phellinus pomaceus (Pers.) Maire	Prunus cerasifera Ehrh.	38.403	56.757
Phellinus pomaceus (Pers.) Maire	Prunus cerasifera Ehrh.	38.403	56.757
Phellinus pomaceus (Pers.) Maire	Prunus microcarpa C.A. Mey.	38.403	56.757
Phellinus pomaceus (Pers.) Maire	Prunus microcarpa C.A. Mey.	38.403	56.757
Phellinus pomaceus (Pers.) Maire	Prunus microcarpa C.A. Mey.	38.403	56.757
Phellinus pomaceus (Pers.) Maire	Prunus microcarpa C.A. Mey.	38.56667	56.73333
Phellinus pomaceus (Pers.) Maire	Prunus turcomanica (Lincz.) Kitam.	38.47056	57.03854
Phellinus pomaceus (Pers.) Maire	Prunus turcomanica (Lincz.) Kitam.	38.47056	57.03854
Phellinus pomaceus (Pers.) Maire	Prunus turcomanica (Lincz.) Kitam.	38.47056	57.03854
Phellinus pomaceus (Pers.) Maire	Berberis turcomanica Kar. ex Ledeb.	38.41578	57.41281
Phellinus pomaceus (Pers.) Maire	Prunus sp.	41.602968	70.118626
Phellinus pomaceus (Pers.) Maire	Prunus cerasifera Ehrh	41.599751	70.030688
Phellinus pomaceus (Pers.) Maire	living fruit trees	41.596708	70.024966
Phellinus pomaceus (Pers.) Maire	Prunus mahaleb	41.570393	70.095594

Phellinus pomaceus (Pers.) Maire	Prunus erythrocarpa (Nevski) Gilli	41.570393	70.095594
Phellinus pomaceus (Pers.) Maire	Salix sp.	41.570393	70.095594
Phellinus pomaceus (Pers.) Maire	Prunus cerasifera	41.666887	69.745518
Phellinus pomaceus (Pers.) Maire	Prunus sp.	41.646619	69.767938
Phellinus pomaceus (Pers.) Maire	Prunus tree	41.659014	69.753197
Phellinus pomaceus (Pers.) Maire	Prunus sp.	41.670301	69.775172
Phellinus pomaceus (Pers.) Maire	Prunus dulcis (Mill.) D.A.Webb	41.614381	69.913579
Phellinus pomaceus (Pers.) Maire	Prunus griffithii var. tianshanica (Pojark.)	41.610149	69.916995
Phellinus pomaceus (Pers.) Maire	Prunus griffithii var. tianshanica (Pojark.)	41.615322	69.915674
Phellinus pomaceus (Pers.) Maire	Prunus cerasifera	41.608992	69.91789
Phellinus pomaceus (Pers.) Maire	Prunus cerasifera	41.601383	69.922781
Phellinus pomaceus (Pers.) Maire	Prunus mahaleb	41.488485	69.882738
Phellinus pomaceus (Pers.) Maire	Prunus sp.	41.486922	69.93755
Phellinus pomaceus (Pers.) Maire	Prunus sp.	41.875512	70.317761
Phellinus pomaceus (Pers.) Maire	Crataegus chlorocarpa Lenné & K.Koch	41.384301	70.152864
Phellinus pomaceus (Pers.) Maire	Lonicera sp.	41.577649	69.987999
Phellinus pomaceus (Pers.) Maire	Juglans regia	41.188089	69.930881
Phellinus pomaceus (Pers.) Maire	Celtis australis subsp. caucasica	41.284277	70.197661
Phellinus pomaceus (Pers.) Maire	Prunus spp.	41.350244	69.95722
Phellinus pomaceus (Pers.) Maire	Malus domestica	41.127633	70.136652
Phellinus pomaceus (Pers.) Maire	Prunus persica (L.) Batsch	40.719433	72.434532
Phellinus pomaceus (Pers.) Maire	Cydonia oblonga Mill.	41.072487	70.70488
Phellinus pomaceus (Pers.) Maire	Prunus domestica L.	40.720202	72.438935
Phellinus pomaceus (Pers.) Maire	Malus domestica	40.709966	72.432466
Phellinus pomaceus (Pers.) Maire	on a dry branch	41.27	69.92
Phellinus pomaceus (Pers.) Maire	Prunus sp.	41.55618746	70.14362812
Phellinus pomaceus (Pers.) Maire	Prunus sp.	41.6069715	70.11766434
Phellinus pomaceus (Pers.) Maire	Prunus sp.	41.6069715	70.11766434
Phellinus pomaceus (Pers.) Maire	Prunus sp.	41.6069715	70.11766434
Phellinus pomaceus (Pers.) Maire	Prunus sp.	39.653785	68.38169
Phellinus pomaceus (Pers.) Maire	Prunus sp.	41.720816	70.064571
Phellinus pomaceus (Pers.) Maire	Malus sp.	39.653785	68.38169
Phellinus pomaceus (Pers.) Maire	Prunus sp.	38.884236	67.448324
Phellinus pomaceus (Pers.) Maire	Prunus persica (L.) Batsch	40.720202	72.438935
Phellinus pomaceus (Pers.) Maire	Cydonia oblonga Mill.	40.905696	70.791573
Phellinus pomaceus (Pers.) Maire	Prunus domestica L.	40.720202	72.438935
Phellinus pomaceus (Pers.) Maire	Malus domestica	40.709966	72.432466
Phellinus tremulae (Bondartsev) Bondar	Populus tremula L.	53.279008333	64.206913889
Phellinus tremulae (Bondartsev) Bondar	Populus tremula L.	52.406055556	63.912513889
Phellinus tremulae (Bondartsev) Bondar	Populus tremula L.	51.526061111	64.430533333
Phellinus tremulae (Bondartsev) Bondar	Populus tremula L.	50.451458333	83.447233333
Phellinus tremulae (Bondartsev)	Populus tremula L.	49.208261111	86.347266667

Bondar			
Phellinus tremulae (Bondartsev) Bondar	Populus tremula L.	48.780963889	86.018394444
Phellinus tremulae (Bondartsev) Bondar	Populus tremula L.	49.208261111	86.347266667
Phellinus tremulae (Bondartsev) Bondart	Populus tremula	41.20107	69.917982
Phellinus tremulae (Bondartsev) Bondart	Populus sp.	40.424711	71.187137
Phellinus tremulae (Bondartsev) Bondart	Populus sp.	41.925186	70.5668
Phellinus tremulae (Bondartsev) Bondart	Populus tremula	38.838632	67.333681
Phellinus tremulae (Bondartsev) Bondart	Populus tremula	39.631762	67.176535
Phellinus tremulae (Bondartsev) Bondart	Populus sp.	40.611462	66.673066
Phellinus tremulae (Bondartsev) Bondart	Populus tremula	39.582945	67.170006
Phellinus tremulae (Bondartsev) Bondart	Populus sp.	40.424711	71.187137
Phylloporia ampelina (Bondartsev and Si	Vitis vinifera L.	41.550301	70.02407
Phylloporia ephedrae (Woron.) Parmasto	Ephedra sp.	43.342297222	77.610194444
Phylloporia ephedrae (Woron.) Parmasto	Ephedra equisetina Bunge	41.913552778	70.004925000
Phylloporia ephedrae (Woron.) Parmasto	Ephedra intermedia Schrenk & C.A.Mey.	43.266436111	78.965275000
Phylloporia ephedrae (Woron.) Parmasto	Rosa sp.	38.8042	68.8075
Phylloporia ephedrae (Woron.) Parmasto	Rosa sp.	38.8042	68.8075
Phylloporia ephedrae (Woron.) Parmasto	Rosa sp.	38.8042	68.8075
Phylloporia ephedrae (Woron.) Parmasto	Rosa sp.	38.8042	68.8075
Phylloporia ephedrae (Woron.) Parmasto	Prunus bucharica (Korsh.) Hand.-Mazz.	38.83827	68.89847
Phylloporia ephedrae (Woron.) Parmasto	Rosa sp.	38.8042	68.8075
Phylloporia ephedrae (Woron.) Parmasto	Crataegus sp.	38.79	69.36
Phylloporia ephedrae (Woron.) Parmasto	Rosa sp.	38.4539	70.7866
Phylloporia ephedrae (Woron.) Parmasto	Rosa × karakalensis Kult.	38.403	56.757
Phylloporia ephedrae (Woron.) Parmasto	Rosa canina L.	38.403	56.757
Phylloporia ephedrae (Woron.) Parmasto	Ephedra intermedia Schrenk & C.A.Mey.	38.41578	57.41281
Phylloporia ephedrae (Woron.) Parmasto	Ephedra intermedia Schrenk & C.A.Mey.	38.403	56.757
Phylloporia ephedrae (Woron.) Parmasto	Ephedra intermedia Schrenk & C.A.Mey.	38.403	56.757
Phylloporia ephedrae (Woron.) Parmasto	Ephedra intermedia Schrenk & C.A.Mey.	38.403	56.757
Phylloporia ephedrae (Woron.) Parmasto	Ephedra intermedia Schrenk & C.A.Mey.	38.403	56.757
Phylloporia ephedrae (Woron.) Parmasto	Ephedra intermedia Schrenk & C.A.Mey.	38.403	56.757
Phylloporia ephedrae (Woron.) Parmasto	Ephedra sp.	38.47056	57.03854

Phylloporia ephedrae (Woron.) Parmasto	Crataegus pseudoheterophylla subsp. tur	38.47056	57.03854
Phylloporia ephedrae (Woron.) Parmasto	Ephedra sp.	38.47056	57.03854
Phylloporia ephedrae (Woron.) Parmasto	Ephedra sp.	38.47056	57.03854
Phylloporia ephedrae (Woron.) Parmasto	Crataegus × zangezura nothosubsp. pseu	38.403	56.757
Phylloporia ephedrae (Woron.) Parmasto	Ephedra sp.	38.403	56.757
Phylloporia ephedrae (Woron.) Parmasto	Chrysojasminum fruticans (L.) Banfi	38.63994	59.47209
Phylloporia ephedrae (Woron.) Parmasto	Chrysojasminum fruticans (L.) Banfi	38.63994	59.47209
Phylloporia ephedrae (Woron.) Parmasto	Ephedra sp.	38.63994	59.47209
Phylloporia ephedrae (Woron.) Parmasto	Ephedra sp.	38.63994	59.47209
Phylloporia ephedrae (Woron.) Parmasto	Ephedra sp.	38.63994	59.47209
Phylloporia ephedrae (Woron.) Parmasto	Ephedra sp.	37.90583684	58.0689893
Phylloporia ephedrae (Woron.) Parmasto	Ephedra sp.	37.90583684	58.0689893
Phylloporia ephedrae (Woron.) Parmasto	Ephedra sp.	37.90583684	58.0689893
Phylloporia ephedrae (Woron.) Parmasto	Ephedra intermedia Schrenk & C.A.Mey.	38.63994	59.47209
Phylloporia ephedrae (Woron.) Parmasto	Ephedra intermedia Schrenk & C.A.Mey.	38.63994	59.47209
Phylloporia ephedrae (Woron.) Parmasto	Ephedra intermedia Schrenk & C.A.Mey.	38.63994	59.47209
Phylloporia ephedrae (Woron.) Parmasto	Ephedra intermedia Schrenk & C.A.Mey.	38.403	56.757
Phylloporia ephedrae (Woron.) Parmasto	Ephedra intermedia Schrenk & C.A.Mey.	38.393	56.724
Phylloporia ephedrae (Woron.) Parmasto	Ephedra intermedia Schrenk & C.A.Mey.	35.29534	62.39485
Phylloporia ephedrae (Woron.) Parmasto	Ephedra equisetina Bunge	41.338978	69.985872
Phylloporia ephedrae (Woron.) Parmasto	Ephedra equisetina Bunge	41.309915	70.094306
Phylloporia ephedrae (Woron.) Parmasto	Ephedra equisetina Bunge	41.375683	70.160285
Phylloporia pulla (Mont. & Berk.) Decock	Berberis oblonga (Rgl.) C.K. Schn	40.44676111	72.74754722
Phylloporia ribis (Schumach.) Ryvarden	Berberis integerrima Bunge	43.267177778	78.969677778
Phylloporia ribis (Schumach.) Ryvarden	Berberis integerrima Bunge	43.266436111	78.965275000
Phylloporia ribis (Schumach.) Ryvarden	Berberis heteropoda Schrenk ex Fisch. &	43.411277778	78.014102778
Phylloporia ribis (Schumach.) Ryvarden	Rosa spinosissima L.	43.267177778	78.969677778
Phylloporia ribis (Schumach.) Ryvarden	Rosa spinosissima L.	43.266436111	78.965275000
Phylloporia ribis (Schumach.) Ryvarden	Euonymus semenovii Regel & Herder	43.234816667	76.976277778
Phylloporia ribis (Schumach.) Ryvarden	Euonymus semenovii Regel & Herder	43.107083333	76.912991667
Phylloporia ribis (Schumach.) Ryvarden	Berberis oblonga (Rgl.) C.K. Schn	42.10674722	77.62025833
Phylloporia ribis (Schumach.) Ryvarden	Berberis oblonga (Rgl.) C.K. Schn	42.10064167	77.61454722
Phylloporia ribis (Schumach.) Ryvarden	Berberis oblonga (Rgl.) C.K. Schn	42.32520833	78.23817778
Phylloporia ribis (Schumach.) Ryvarden	Cotoneaster hissaricus Pojark.	38.8042	68.8075
Phylloporia ribis (Schumach.) Ryvarden	Crataegus turkestanica Pojark.	38.47056	57.03854

Phylloporia ribis (Schumach.) Ryvarden	Ephedra intermedia Schrenk & C.A.Mey.	38.403	56.757
Phylloporia yuchengii Gafforov, Tomšovs	Juglans regia	42.047723	70.428716
Phylloporia yuchengii Gafforov, Tomšovs	Prunus sp.	41.688477	69.937439
Phylloporia yuchengii Gafforov, Tomšovs	Juglans regia	42.047723	70.428716
Phylloporia yuchengii Gafforov, Tomšovs	Crataegus sp.	41.615244	69.928928
Phylloporia yuchengii Gafforov, Tomšovs	angiosperm trunk and stem	41.624353	69.770446
Phylloporia yuchengii Gafforov, Tomšovs	angiosperm trunk and stem	41.608992	69.91789
Phylloporia yuchengii Gafforov, Tomšovs	Crataegus pseudoheterophylla subsp. tur	40.62201	66.67779
Phylloporia yuchengii Gafforov, Tomšovs	unknown woody branches	39.655088	68.372883
Phylloporia yuchengii Gafforov, Tomšovs	Populus sp.	38.971261	66.697895
Phylloporia yuchengii Gafforov, Tomšovs	Morus alba	38.955723	66.697365
Phylloporia yuchengii Gafforov, Tomšovs	Morus alba	38.955755	66.697365
Porodaedalea chrysoloma (Fr.) Fiasson &	Picea schrenkiana Fisch & C.A. Mey.	43.138405556	76.906508333
Porodaedalea chrysoloma (Fr.) Fiasson &	Picea schrenkiana Fisch & C.A. Mey.	43.114208333	77.078522222
Porodaedalea chrysoloma (Fr.) Fiasson &	Picea schrenkiana Fisch & C.A. Mey.	43.248022222	77.225608333
Porodaedalea chrysoloma (Fr.) Fiasson &	Picea schrenkiana Fisch. & C.A.Mey.	42.76636944	77.47373333
Porodaedalea chrysoloma (Fr.) Fiasson &	Picea schrenkiana Fisch. & C.A.Mey.	42.17189167	77.73572500
Porodaedalea chrysoloma (Fr.) Fiasson &	Picea schrenkiana Fisch. & C.A.Mey.	42.32539722	78.23938333
Porodaedalea chrysoloma (Fr.) Fiasson &	Picea schrenkiana Fisch. & C.A.Mey.	42.45245000	78.52227500
Porodaedalea chrysoloma (Fr.) Fiasson &	Picea schrenkiana Fisch. & C.A.Mey.	42.80111389	77.65728333
Porodaedalea pini (Brot.) Murrill.	Picea schrenkiana Fisch & C.A. Mey.	43.055647222	78.406952778
Porodaedalea pini (Brot.) Murrill.	Picea schrenkiana Fisch & C.A. Mey.	45.356747222	79.932069444
Porodaedalea pini (Brot.) Murrill.	Pinus sylvestris L.	52.425602778	63.611113889
Porodaedalea pini (Brot.) Murrill.	Pinus sylvestris L.	53.792700000	64.221975000
Porodaedalea pini (Brot.) Murrill.	Pinus sylvestris L.	52.428422222	63.660091667
Porodaedalea pini (Brot.) Murrill.	Pinus sylvestris L.	52.573444444	68.828575000
Porodaedalea pini (Brot.) Murrill.	Pinus sylvestris L.	52.605794444	68.871847222
Porodaedalea pini (Brot.) Murrill.	Pinus sylvestris L.	52.628847222	68.861863889
Porodaedalea pini (Brot.) Murrill.	Pinus sylvestris L.	52.652730556	68.839980556
Porodaedalea pini (Brot.) Murrill.	Pinus sylvestris L.	52.635869444	70.479102778
Porodaedalea pini (Brot.) Murrill.	Pinus sylvestris L.	52.937094444	70.239563889
Porodaedalea pini (Brot.) Murrill.	Pinus sylvestris L.	52.949438889	70.256361111
Porodaedalea pini (Brot.) Murrill.	Pinus sylvestris L.	49.379947222	75.364150000
Porodaedalea pini (Brot.) Murrill.	Abies sibirica Ledeb.	50.451458333	83.447233333
Porodaedalea pini (Brot.) Murrill.	Abies sibirica Ledeb.	49.216425000	86.327058333

Porodaedalea pini (Brot.) Murrill.	Picea schrenkiana Fisch & C.A. Mey.	43.155969444	77.035452778
Porodaedalea pini (Brot.) Murrill.	Picea schrenkiana Fisch & C.A. Mey.	43.057322222	76.987330556
Porodaedalea pini (Brot.) Murrill.	Picea schrenkiana Fisch & C.A. Mey.	45.335852778	79.831844444
Porodaedalea pini (Brot.) Murrill.	Picea schrenkiana Fisch & C.A. Mey.	43.380813889	80.385722222
Porodaedalea pini (Brot.) Murrill.	Picea schrenkiana Fisch & C.A. Mey.	43.379972222	80.348666667
Porodaedalea pini (Brot.) Murrill.	Picea schrenkiana Fisch & C.A. Mey.	43.258425000	80.644713889
Porodaedalea pini (Brot.) Murrill.	Picea schrenkiana Fisch. & C.A.Mey.	42.32682778	78.23534444
Porodaedalea pini (Brot.) Murrill.	Picea schrenkiana Fisch. & C.A.Mey.	42.20108611	77.69113056
Porodaedalea pini (Brot.) Murrill.	Picea schrenkiana Fisch. & C.A.Mey.	42.05399722	77.59780833
Porodaedalea pini (Brot.) Murrill.	Picea schrenkiana Fisch. & C.A.Mey.	42.49298333	78.09822500
Porodaedalea pini (Brot.) Murrill.	Picea schrenkiana Fisch. & C.A.Mey.	42.60358611	79.02620278
Porodaedalea pini (Brot.) Murrill, Bull.	Pinus pallasiana D. Don	41.342852	69.313558
Porodaedalea pini (Brot.) Murrill, Bull.	dried stem of angiosperm	40.817232	71.334759
Sanghuangporus lonicerinus (Bondartsev	Lonicera sp.	45.598294444	80.347111111
Sanghuangporus lonicerinus (Bondartsev	Lonicera sp.	45.378338889	80.036611111
Sanghuangporus lonicerinus (Bondartsev	Lonicera caerulea subsp. altaica (Pall.) Gl	43.684950000	79.400947222
Sanghuangporus lonicerinus (Bondartsev	Lonicera webbiana Wall. ex DC.	42.148113889	70.477988889
Sanghuangporus lonicerinus (Bondartsev	Lonicera nummulariifolia Jaub. & Spach	42.273622222	70.668616667
Sanghuangporus lonicerinus (Bondartsev	Lonicera nummulariifolia Jaub. & Spach	42.775069444	69.746069444
Sanghuangporus lonicerinus (Bondartsev	Lonicera xylosteum L.	50.463769444	65.920880556
Sanghuangporus lonicerinus (Bondartsev	Lonicera sp.	45.510191667	80.633930556
Sanghuangporus lonicerinus (Bondartsev	Lonicera sp.	44.119708333	79.967069444
Sanghuangporus lonicerinus (Bondartsev	Lonicera sp.	45.598294444	80.347111111
Sanghuangporus lonicerinus (Bondartsev	Lonicera sp.	43.570058333	78.319444444
Sanghuangporus lonicerinus (Bondartsev	Lonicera sp.	43.666263889	79.385169444
Sanghuangporus lonicerinus (Bondartsev	Lonicera sp.	43.620716667	78.287141667
Sanghuangporus lonicerinus (Bondartsev	Lonicera sp.	43.099577778	76.926086111
Sanghuangporus lonicerinus (Bondartsev	Lonicera sp.	43.096111111	76.948919444
Sanghuangporus lonicerinus (Bondartsev	Lonicera sp.	43.378380556	77.594727778
Sanghuangporus lonicerinus (Bondartsev	Lonicera sp.	42.49095000	78.50570556
Sanghuangporus lonicerinus (Bondartsev	Lonicera	38.71766179	70.5331707

Sanghuangporus lonicerinus (Bondartsev	Lonicera	38.90468	68.81088
Sanghuangporus lonicerinus (Bondartsev	Lonicera	38.90468	68.81088
Sanghuangporus lonicerinus (Bondartsev	Lonicera	38.90468	68.81088
Sanghuangporus lonicerinus (Bondartsev	Lonicera	38.90468	68.81088
Sanghuangporus lonicerinus (Bondartsev	Lonicera	38.76998465	69.38261032
Sanghuangporus lonicerinus (Bondartsev	Lonicera	38.80941	68.81765
Sanghuangporus lonicerinus (Bondartsev	Lonicera	38.8042	68.8075
Sanghuangporus lonicerinus (Bondartsev	Lonicera nummulariifolia Jaub. & Spach	38.8042	68.8075
Sanghuangporus lonicerinus (Bondartsev	Lonicera	38.76998465	69.38261032
Sanghuangporus lonicerinus (Bondartsev	Lonicera	38.76998465	69.38261032
Sanghuangporus lonicerinus (Bondartsev	Lonicera	38.8106	68.82403
Sanghuangporus lonicerinus (Bondartsev	Lonicera	38.8106	68.82403
Sanghuangporus lonicerinus (Bondartsev	Lonicera	38.8106	68.82403
Sanghuangporus lonicerinus (Bondartsev	Lonicera	38.8042	68.8075
Sanghuangporus lonicerinus (Bondartsev	Lonicera	38.8042	68.8075
Sanghuangporus lonicerinus (Bondartsev	Lonicera	38.8042	68.8075
Sanghuangporus lonicerinus (Bondartsev	Lonicera	38.5507	71.7568
Sanghuangporus lonicerinus (Bondartsev	Lonicera	38.90468	68.81088
Sanghuangporus lonicerinus (Bondartsev	Lonicera	38.37833584	69.34431412
Sanghuangporus lonicerinus (Bondartsev	Lonicera sp.	38.27316	57.1789
Sanghuangporus lonicerinus (Bondartsev Spach	Lonicera nummulariifolia Jaub. &	38.56667	56.73333
Sanghuangporus lonicerinus (Bondartsev Spach	Lonicera nummulariifolia Jaub. &	38.56667	56.73333
Sanghuangporus lonicerinus (Bondartsev	Lonicera	38.56667	56.73333
Sanghuangporus lonicerinus (Bondartsev Spach	Lonicera nummulariifolia Jaub. &	38.27316	57.1789
Sanghuangporus lonicerinus (Bondartsev Spach	Lonicera nummulariifolia Jaub. &	38.47056	57.03854
Sanghuangporus lonicerinus (Bondartsev	Lonicera	38.41578	57.41281
Sanghuangporus lonicerinus (Bondartsev Spach	Lonicera nummulariifolia Jaub. &	38.47056	57.03854
Sanghuangporus lonicerinus (Bondartsev	Lonicera	38.403	56.757
Sanghuangporus lonicerinus (Bondartsev Spach	Lonicera nummulariifolia Jaub. &	38.56667	56.73333
Sanghuangporus lonicerinus (Bondartsev	Lonicera nummulariifolia Jaub. &	38.56667	56.73333

	Spach				
Sanghuangporus lonicerinus (Bondartsev	Lonicera nummulariifolia Jaub. & Spach		38.56667		56.73333
Sanghuangporus lonicerinus (Bondartsev	Lonicera		38.41578		57.41281
Sanghuangporus lonicerinus (Bondartsev	Lonicera		38.41578		57.41281
Sanghuangporus lonicerinus (Bondartsev	Lonicera nummulariifolia Jaub. & Spach		38.47056		57.03854
Sanghuangporus lonicerinus (Bondartsev	Lonicera sp.	41.917896		70.452465	
Sanghuangporus lonicerinus (Bondartsev	Lonicera sp.	41.950939		70.441038	
Sanghuangporus lonicerinus (Bondartsev	Lonicera nummulariifolia Jaub. and Spac	41.494422		69.973985	
Sanghuangporus lonicerinus (Bondartsev	Lonicera sp.	41.509234		70.0466	
Sanghuangporus lonicerinus (Bondartsev	Lonicera sp.	41.638929		70.217179	
Sanghuangporus lonicerinus (Bondartsev	Lonicera sp.	41.825651		70.50329	
Sanghuangporus lonicerinus (Bondartsev	Lonicera sp.	41.584048		70.089723	
Sanghuangporus lonicerinus (Bondartsev	Lonicera sp.	41.570393		70.095594	
Sanghuangporus lonicerinus (Bondartsev	Lonicera sp.	41.601383		69.922781	
Sanghuangporus lonicerinus (Bondartsev	Lonicera nummulariifolia	41.571334		70.082208	
Sanghuangporus lonicerinus (Bondartsev	Lonicera sp.	41.624458		69.770557	
Sanghuangporus lonicerinus (Bondartsev	Lonicera sp.	41.938872		70.542957	
Sanghuangporus lonicerinus (Bondartsev	Lonicera sp.	41.843467		70.448682	
Sanghuangporus lonicerinus (Bondartsev	Acer sp.	41.925905		70.428203	
Sanghuangporus lonicerinus (Bondartsev	Acer tataricum subsp. semenovii	41.116208		70.096456	
Sanghuangporus lonicerinus (Bondartsev	deciduous trunk	41.316018		69.829899	
Sanghuangporus lonicerinus (Bondartsev	deciduous trunk	41.174519		69.841118	
Sanghuangporus lonicerinus (Bondartsev	Lonicera altmannii Regel & Schmalh.	41.509234		70.0466	
Sanghuangporus lonicerinus (Bondartsev	Lonicera sp.		41.56428047		70.05453535
Sanghuangporus lonicerinus (Bondartsev	Lonicera sp.		41.52443819		70.02179146
Sanghuangporus lonicerinus (Bondartsev	Lonicera sp.		41.52443819		70.02179146
Sanghuangporus lonicerinus (Bondartsev	Lonicera sp.	40.559126		66.744285	
Sanghuangporus lonicerinus (Bondartsev	Lonicera sp.	39.583406		68.481319	
Sanghuangporus lonicerinus (Bondartsev	Lonicera microphylla Willd. ex Schult.	39.791276°		68.076034°	
Tropicoporus linteus (Berk. and M.A. Cur	angiosperm woody plants	41.66507500		72.09509167	
Tropicoporus linteus (Berk. and M.A. Cur	Lonicera sp.		38.63994		59.47209
Tropicoporus linteus (Berk. and M.A. Cur	Lonicera sp.		38.63994		59.47209
Tropicoporus linteus (Berk. and M.A. Cur	Lonicera sp.		38.63994		59.47209
Tropicoporus linteus (Berk. and M.A. Cur	Lonicera sp.		38.63994		59.47209

Tropicoporus linteus (Berk. and M.A. Cur Lonicera sp.		38.63994		59.47209
Tropicoporus linteus (Berk. and M.A. Cur Rosa fedtschenkoana Regel	41.121723		70.11301	
Tropicoporus linteus (Berk. and M.A. Cur living trunk and stem of angiosperm woo	41.295324		69.708483	
Tropicoporus linteus (Berk. and M.A. Cur Salix sp.	41.361643		70.176321	
Tropicoporus linteus (Berk. and M.A. Cur Lonicera sp.	41.309915		70.094306	
Tropicoporus linteus (Berk. and M.A. Cur Salix wilhelmsiana	40.711843		72.433273	
Tropicoporus linteus (Berk. and M.A. Cur Salix wilhelmsiana	40.720956		72.436264	
Tropicoporus linteus (Berk. and M.A. Cur Quercus sp.	39.599541		67.197633	
Tropicoporus linteus (Berk. and M.A. Cur Populus sp.	40.5926		66.729802	
Tropicoporus linteus (Berk. and M.A. Cur Acer sp.	39.600223		68.350032	
Tropicoporus linteus (Berk. and M.A. Cur Ulmus sp.	38.864904		67.415373	
